# Chemoselective cysteine or disulfide modification *via* single atom substitution in chloromethyl acryl reagents†

**DOI:** 10.1039/d1sc03250j

**Published:** 2021-09-09

**Authors:** Lujuan Xu, Maria J. S. A. Silva, Pedro M. P. Gois, Seah Ling Kuan, Tanja Weil

**Affiliations:** a Max Planck Institute for Polymer Research Ackermannweg 10 55128 Mainz Germany weil@mpip-mainz.mpg.de; b Institute of Inorganic Chemistry I, Ulm University Albert-Einstein-Allee 11 89081 Ulm Germany; c Research Institute for Medicines (iMed.ULisboa), Faculty of Pharmacy, Universidade de Lisboa 1649-003 Lisbon Portugal

## Abstract

The development of bioconjugation chemistry has enabled the combination of various synthetic functionalities to proteins, giving rise to new classes of protein conjugates with functions well beyond what Nature can provide. Despite the progress in bioconjugation chemistry, there are no reagents developed to date where the reactivity can be tuned in a user-defined fashion to address different amino acid residues in proteins. Here, we report that 2-chloromethyl acryl reagents can serve as a simple yet versatile platform for selective protein modification at cysteine or disulfide sites by tuning their inherent electronic properties through the amide or ester linkage. Specifically, the 2-chloromethyl derivatives (acrylamide or acrylate) can be obtained *via* a simple and easily implemented one-pot reaction based on the coupling reaction between commercially available starting materials with different end-group functionalities (amino group or hydroxyl group). 2-Chloromethyl acrylamide reagents with an amide linkage favor selective modification at the cysteine site with fast reaction kinetics and near quantitative conversations. In contrast, 2-chloromethyl acrylate reagents bearing an ester linkage can undergo two successive Michael reactions, allowing the selective modification of disulfides bonds with high labeling efficiency and good conjugate stability.

## Abstract 2

2-Chloromethyl acryl derivatives (acrylamides and acrylates) can serve as simple and versatile bioconjugation reagents to achieve site-selective cysteine and disulfide modification on demand and with high efficiency.

## Graphical abstract

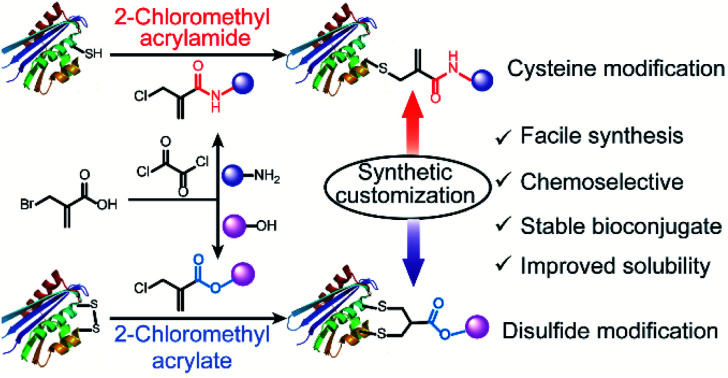

## 1. Introduction

Proteins are an emerging class of biotherapeutics with high target affinity and specificity.^1–3^ Site-selective modification of proteins enables the incorporation of desired synthetic functionalities into proteins at distinct sites, which combine the advantages from both the synthetic world and Nature for the construction of protein bioconjugates with novel functional characteristics.^4–10^ Chemical approaches for protein modification allow the straightforward attachment of the desired functionalities at natural amino acid residues on the protein surface, thereby eliminating the need for tedious genetic engineering.^11^ Among these, unpaired cysteines are considered the most sought-after targets owing to the high nucleophilicity and versatile chemistry landscapes of thiol groups.^12–15^ In addition, disulfide bonds have also emerged as attractive modification sites to incorporate tailored functionalities, as a lot of therapeutic relevant proteins or peptides, *e.g.* antibodies or their antigen-binding fragments, contain at least one solvent-accessible disulfide bond.^16,17^

Maleimides constitute a group of widely-used cysteine bioconjugation reagents due to their fast and efficient reactions with thiols.^12^ Besides that, a variety of structurally diverse reagents have also been reported for cysteine modification in order to improve the stability of the resultant bioconjugates as well as retaining similar reaction kinetics.^18,19^ However, the strategies for disulfide modification are much less explored and the current toolset is limited to five to six conjugation methods available in the literature.^20–26^ Moreover, the reagents developed to date mainly target a single amino acid residue, for example a cysteine residue or a disulfide bond. Besides the (bromo)maleimides,^23^ 3-bromo-5-methylene pyrrolones^27^ and diethynyl phosphinates,^28^ there are only a few bioconjugation reagents that can provide a broad spectrum scaffold to address both cysteines and disulfides with high labeling efficiency. Such a strategy is more advantageous compared to reinventing a novel scaffold for every single purpose. Therefore, the development of such a bioconjugation approach, which enables the selective modification at target amino acid residues in a user-defined fashion with great ease, would be highly advantageous to enrich the existing toolbox and also to enable non-experts to conduct such protein labeling reactions.

This prompts us to rethink the strategies and enormous possibilities offered by synthetic chemistry. In fact, modern synthetic technologies provide immense flexibility and potential to access structurally diverse reagents, which allows for the customization of their reactivities at the atomic level. From this perspective, we envisioned that multifunctional bioconjugation reagents, which are capable of targeting the specific residues on demand, can be designed by finely tuning their chemoselectivities with the aid of synthetic chemistry. Inspired by the inherent features of the electron-deficient systems serving as good Michael acceptors for the reactions with nucleophiles on the protein surface,^12^ we proposed 2-halomethyl acryl derivatives (acrylamide or acrylate) as an appropriate option for reactions with thiol groups to accomplish the chemoselective modification of cysteine residues. In addition, considering the different electron-withdrawing properties of the ester and amide bond, we further speculated that a single atom substitution in the acryl position of chloromethyl acryl reagents would influence their reactivity profiles as electrophiles for the second Michael reaction. This, in turn, will allow the customization of their properties to achieve selective modification at either cysteine or disulfide sites.

**Fig. 1:**
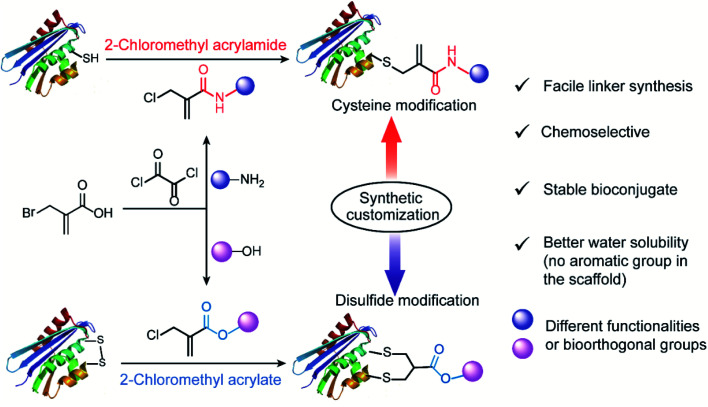
General scheme for 2-chloromethyl acrylamide and acrylate compounds for site-selective protein modification at cysteine or disulfide sites *via* synthetic customization.

Herein, we reported the convenient, one-pot synthesis of 2-chloromethyl derivatives (acrylamide and acrylate) *via* coupling reactions between commercially available 2-(bromomethyl)acrylic acid with different end-group functionalities (amino group or hydroxyl group) (Fig. 1). The inherent chemoselectivity of 2-chloromethyl acrylamide and acrylate are influenced by the different electron-withdrawing properties of the amide and ester linkage, which render them suitable for protein modification at either cysteine or disulfide site. Specifically, we showed that 2-chloromethyl acrylamide compounds containing an amide bond in the scaffold can react with proteins containing a free thiol group *via* a single Michael reaction with near quantitative conversions. By replacing the amide with an ester linkage yielding the respective 2-chloromethyl acrylate reagents, site-selective disulfide modification can be achieved as exemplified by successful modification of three disulfide-containing substrates. In addition, the bioconjugation reagents reported herein are characterized by facile linker synthesis, high water solubility as well as good labeling efficiency.

## 2. Results and discussion

### Synthesis of 2-chloromethyl acrylamide and acrylate compounds

**Scheme 1:**
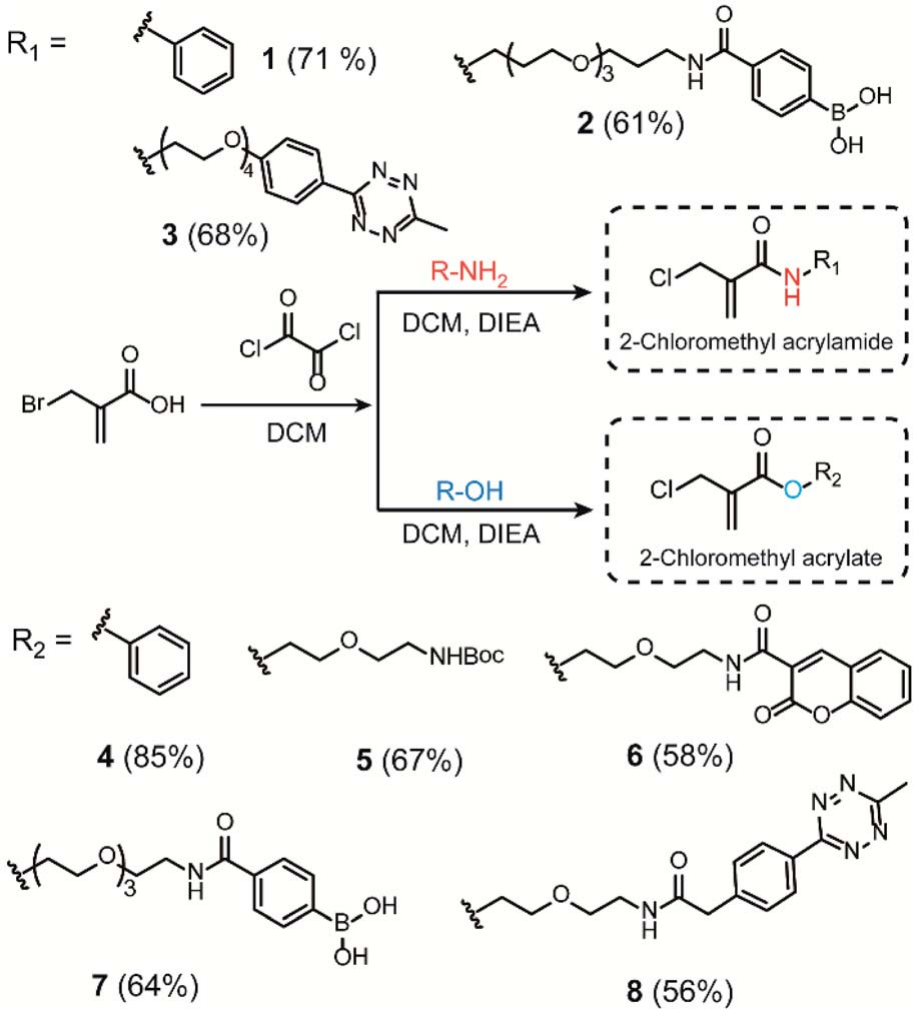
Synthesis route of 2-chloromethyl acrylamide and acrylate derivatives containing different functionalities.

We initiated our study by using the commercially available compound, 2-(bromomethyl)acrylic acid, as starting material to synthesize both 2-halomethyl acrylamide and acrylate bioconjugation reagents (Scheme 1 and S1†). First, 2-(bromomethyl)acrylic acid reacted with oxalyl chloride to convert the carboxylic acid group to acid chloride *in situ*. Thereafter, different end-group nucleophiles, amino or alcohol groups (usually 1.5 to 2 equiv.) were added under basic conditions for further reactions. The respective 2-chloromethyl acrylamide and acrylate were subsequently purified and isolated in moderate yields (Scheme 1). Mass spectrometry (MS) data demonstrated that the bromine atom is completely replaced by the chlorine atom affording the 2-chloromethyl acryl compounds (Fig. S69–S74†). A toolbox containing different functionalities, *e.g.* dye or bioorthogonal groups, was obtained as demonstrated in Scheme 1 underlining the broad applicability of this method. Compared to other disulfide- and cysteine-modification reagents, which require multiple-step synthesis (*e.g.*, allyl sulfones require four-step synthesis^20^), the 2-chloromethyl acryl derivatives are readily available through a straightforward one-pot synthesis from commercially available 2-(bromomethyl)acrylic acid precursors. The simplicity of the synthesis provides fast and efficient access to a broad spectrum of functionalities that are of great interest for bioconjugation.

In addition, compared to the reported cysteine and disulfide modification reagents, *e.g.* carbonylacrylic reagent^29^ or allyl sulfone reagents^20^ that contain a hydrophobic phenyl group, the 2-chloromethyl acryl derivatives do not contain any aromatic group in the scaffold, where they are estimated to have lower partition coefficients (*n*-octanol to water, log *P*_o/w_) (Fig. S2 and S3†) indicating improved water solubility. Furthermore, the stability of the bioconjugation reagents in different aqueous environment represents an important consideration for their subsequent usage. The stability of the 2-chloromethyl acrylamide and acrylate compounds was evaluated by incubating compound 3 and compound 4 at three different pH (pH 6, 7 and 8), and the HPLC data indicated that they remained stable over a time course of 36 hours without any degradation (Fig. S4–S9†). In contrast, maleimides reagents, which are the most commonly used bioconjugation reagents for cysteine functionalization, easily hydrolyze to nonreactive maleic amides, especially at basic pH (*t*_1/2_ < two hours) (Fig. S10–S12†).

### Chemoselectivity of 2-chloromethyl acrylamide and acrylate towards thiol groups

**Fig. 2:**
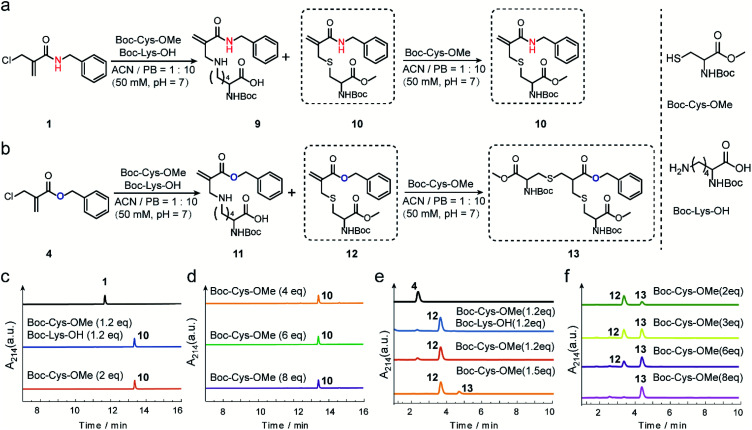
(a) Reaction scheme between 2-chloromethyl acrylamide and Boc-Cys-OMe (and Boc-Lys-OH). (b) Reaction scheme between 2-chloromethyl acrylate and Boc-Cys-OMe (and Boc-Lys-OH). (c) LC trace of the reaction between 2-chloromethyl acrylamide and Boc-Cys-OMe (and Boc-Ly-OH). (d) LC trace of 2-chloromethyl acrylamide with Boc-Cys-OMe (from 4 equiv. to 8 equiv.). (e) LC trace of the reaction between 2-chloromethyl acrylate and Boc-Cys-OMe (and Boc-Lys-OH) (f) LC trace of 2-chloromethyl acrylate and Boc-Cys-OMe (from 2 equiv. to 8 equiv.).

The reactivity and selectivity of 2-chloromethyl acrylamides and acrylates towards two model amino acids: Boc-Cys-OMe and Boc-Lys-OH (Fig. 2) were evaluated. For 2-chloromethyl acrylamide, compound 1 was incubated with both Boc-Cys-OMe and Boc-Lys-OH in acetonitrile (ACN)/phosphate buffer (PB, pH 7) for four hours (Fig. 2a). Liquid chromatography (LC) data indicated quantitative conversion to the cysteine-modified compound 10 while lysine-modified compound 9 was not observed, which clearly demonstrated its excellent chemoselectivity towards cysteine over lysine residues (Fig. 2c). In addition, the absence of the side reaction with benzylamine during the synthesis of compound 1 also indicated there were no cross-reactions with amino groups (page 5 in ESI†). With increasing Boc-Cys-OMe to 8 equiv., only compound 10 was found in the LC without the observation of the further addition products (Fig. 2d). Further studies demonstrated that compound 10 does not react with other nucleophiles, such as hydroxyl or amino groups, even when used in 30 equiv. excess at 37 °C (Scheme S3 in ESI†). However, if a second thiol functionality was given in very large excess (for example 30 equiv. of compound 18, Scheme S3 in ESI†), the first thiol functionality was eliminated affording compound 15, presumably due to an addition–elimination reaction (Fig. S14 in ESI†).

For 2-chloromethyl acrylate, compound 4 was incubated with both Boc-Cys-OMe and Boc-Lys-OH under the same conditions used for compound 1 (Fig. 2b). The LC trace also revealed the excellent chemoselectivity towards thiol groups as lysine-modified compound 11 was also not observed in the mixture (Fig. 2e). However, in contrast to the reaction with acrylamides, the peak for compound 12 decreased while the signal for compound 13 increased (Fig. 2f), with increasing amounts of Boc-Cys-OMe used. After adding eight equivalents of Boc-Cys-OMe, compound 4 was fully converted to compound 13 with negligible side product formation (Fig. 2f).

These model reactions clearly indicated a pronounced difference in the reactivity of the 2-chloromethyl acrylamide *versus* the acrylate reagents, presumably originating from the amide or ester linkage. The observed reactivity of 2-chloromethyl acrylamide is consistent with literature where it was reported that catalysts and high temperature are required for thiol addition with α,β-unsaturated amides as Michael acceptors.^30,31^ Therefore, we speculate that the second Michael reaction of the 2-chloromethyl acrylamide did not proceed due to the relatively weak electron-withdrawing property of the amide bond, which rendered the α,β-unsaturated amide a poor Michael acceptor.^27^ Taken together, these results demonstrated that 2-chloromethyl acrylamides allow straightforward modification of free cysteines with high efficiency and excellent chemoselectivity. On the other hand, 2-chloromethyl acrylates can undergo two Michael reactions in a successive manner, thereby making them suitable candidates to achieve protein modification at the disulfide sites.

### 2-Chloromethyl acrylamide reagents for cysteine modification

**Fig. 3:**
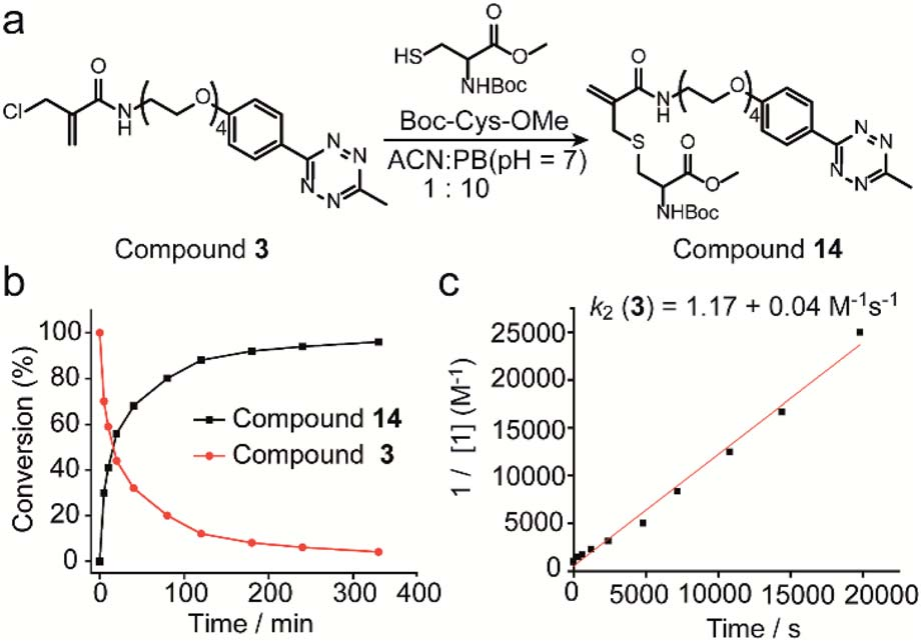
(a) Reaction scheme of compound 3 reacting with Boc-Cys-OMe to form compound 14. (b) Percentage of compound 3 and 14 as determined by the integration of the HPLC peak in comparison to the internal standard at different time points. (c) Experimental determination of the second-order rate constant of the model reaction between compound 3 (1 mM) and Boc-Cys-OMe (1 mM). Details about the calculation and kinetics data were demonstrated in Section 4 in ESI.†

Next, the reaction kinetics were first studied using a model reaction between compound 3 and Boc-Cys-OMe (Fig. 3a). Compound 3 (1 mM) and Boc-Cys-OMe (1 mM) were incubated in ACN/PB (pH 7) mixture (volume ratio: 1 : 10) using Fmoc-Phe-OH (Scheme S4†) as internal standard. At different time intervals, the reaction was monitored by high-performance liquid chromatography (HPLC) (Fig. S17†), and quantification of compounds 3 and 14 overtime was plotted with reference to the internal standard (Fig. 3b). HPLC analysis indicated that 80% of compound 3 was converted to the cysteine-modified compound 14 in less than two hours and near quantitative conversion was achieved in less than six hours (Fig. S17†). The second-order rate constant was determined to be 1.17 M^−1^ s^−1^ with the concentration of compound 3 at 1 mM (Fig. 3c). Although this reaction is slower than the maleimide conjugation (10–1000 M^−1^ s^−1^),^32^ it is still comparable to or even faster than some of the recently reported bioconjugation reagents, *e.g.* ethynylphosphonamidates (0.62 M^−1^ s^−1^),^33^ diethynyl phosphinate (0.47 M^−1^ s^−1^) (Table S1 in ESI†), and some other conventional bioconjugation methods such as oxime ligation (0.001 M^−1^ s^−1^),^34,35^ Pictet–Spengler ligation (0.015 M^−1^ s^−1^)^32^ and strain-promoted azide–alkyne reaction (0.9 M^−1^ s^−1^).^36^

**Fig. 4:**
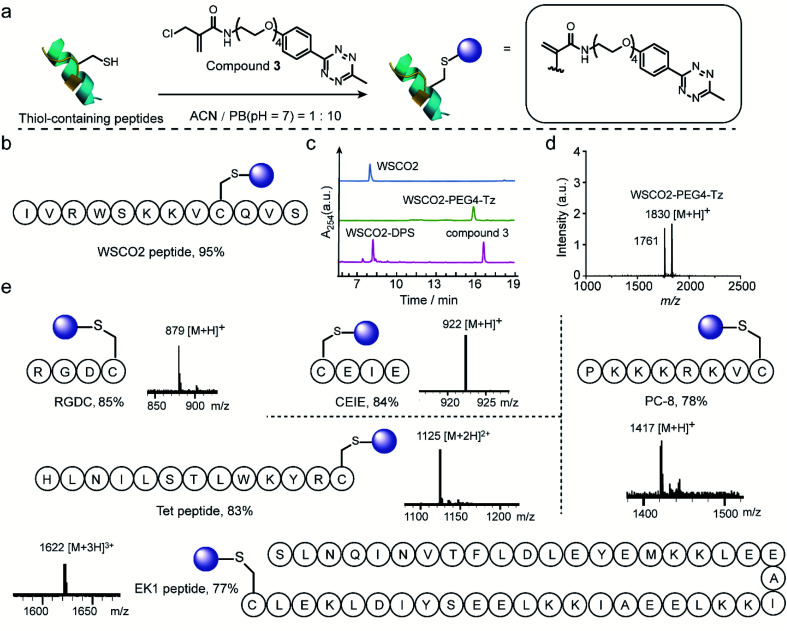
(a) General scheme for the chemoselective modification of thiol-containing peptides with compound 3 in ACN/PB (pH 7) mixture. (b) WSCO2 peptide was selected as model substrate for modification at cysteine site with compound 3. (c) HPLC trace of WSCO2 peptide, crude reaction mixture between WSCO2 and compound 3, WSCO2-DPS and compound 3 (from top to down) which demonstrated the efficient modification efficiency and good chemoselectivity. (d) MALDI-Tof-MS of modified WSCO2 peptide showing a signal at 1830 which is attributed to the WSCO2-PEG4-Tz (calculated: 1830 [M + H]^+^, found: 1830 [M + H]^+^). The signal at 1761 corresponded to the fragmentation product with the double bond breaking at tetrazine moiety, the chemical structure is shown in ESI.† (e) Site-selective modification of different thiol-containing peptides including RGDC (calculated: 879 [M + H]^+^, found: 879 [M + H]^+^), CEIE (calculated: 922 [M + H]^+^, found: 922 [M + H]^+^), PC-8 (calculated: 1417 [M + H]^+^, found: 1417 [M + H]^+^), Tet peptide (calculated: 1125 [M + 2H]^2+^, found: 1125 [M + 2H]^2+^) and EK1 peptide (calculated: 1622 [M + 3H]^3+^, found: 1622 [M + 3H]^3+^) with a tetrazine group.

Thereafter, 2-chloromethyl acrylamide derivatives were applied for cysteine modification on peptide substrates using compound 3 (Fig. 4a). First, the known WSCO2 peptide (sequence: IVRWSKKVCQVS), an endogenous peptide inhibitor of the chemokine CXCR4 receptor that is highly relevant for anti-infectivity in viral infection and anti-migratory effect in cancer,^37^ was selected as bioactive substrate (Fig. 4b). In ACN/PB mixture (1 : 10), one equivalent WSCO2 peptide was incubated with 1.1 equivalents of compound 3 for four hours. HPLC analysis of the crude reaction mixture indicated that more than 95% conversion to the desired modified product (WSCO2-PEG4-Tz) was achieved (Fig. 4c). As a control, the thiol-reactive reagent 4,4′-dithiodipyridine (4-DPS), which is often used for free thiol quantification on proteins *via* a thiol–disulfide exchange reaction (the reaction mechanism is shown in Scheme S6†),^38^ was used to mask the cysteine residue. In this case, no further reaction was observed in the HPLC chromatogram in the presence of compound 3 under the same reaction conditions (Fig. 4c). Taken together, these data clearly indicated that the 2-chloromethyl acrylamide compounds exhibit excellent chemoselectivity in combination with excellent modification efficiency. In addition to WSCO2, five other peptides, including RGDC, CEIE, PC-8, Tet, and EK-1 peptides (sequences and MS of the modified peptides were shown in Fig. 4e and S24–S28†), have also been successfully modified with compound 3. The broad range of substrates used here clearly demonstrates the general applicability of 2-chloromethyl acrylamide compounds for chemoselective modification at cysteine residues.

**Fig. 5:**
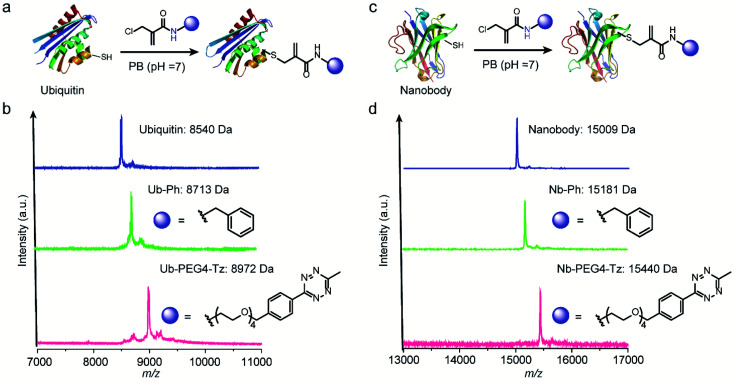
(a) Chemoselective modification of ubiquitin at cysteine site with two different functionalities: a phenyl and a tetrazine group. (b) The successful modification was proved by the MALDI-Tof-MS characterization with a peak at 8713 for Ub-Ph (calculated: 8714 Da, found: 8713 Da) and 8972 for Ub-PEG4-Tz (calculated: 8970 Da, found: 8972 Da). (c) Chemoselective modification of anti-GFP nanobody at cysteine site with two different functionalities: a phenyl and a tetrazine group (d) MALDI-Tof-MS of the modified nanobody with a peak at 15 181 for Nb-Ph (calculated: 15 183 Da, found: 15 181 Da) and 15 440 for Nb-PEG4-Tz (calculated: 15 438 Da, found: 15 440 Da).

After demonstrating the successful modification of the model peptides, we proceeded to functionalize the more complex substrates, *i.e.* proteins. The protein ubiquitin that plays an important role in protein degradation by the proteasome, which contains a cysteine mutation at its K63 position, was selected (Fig. 5a). After incubation of one equivalent ubiquitin with ten equivalents of two different 2-chloromethyl acrylamide derivatives respectively, the desired bioconjugates were obtained. The successful modification was confirmed with the expected *m*/*z* in the MS shown in Fig. 5b. Similarly, if 4-DPS was used to mask the accessible cysteine residue on the protein surface, no reaction was observed even in the presence of ten equivalents of the 2-chloromethyl acrylamides (Fig. S29†). Besides ubiquitin, a single-chain V_H_H antibody domain with specific binding activity against the green fluorescent protein (anti-GFP nanobody) has also been successfully modified with 2-chloromethyl acrylamide derivatives (Fig. 5c). The MALDI-Tof-MS characterization clearly indicated the successful modification with the expected *m*/*z* shown in Fig. 5d.

### 2-Chloromethyl acrylate reagents for disulfide modification

**Fig. 6:**
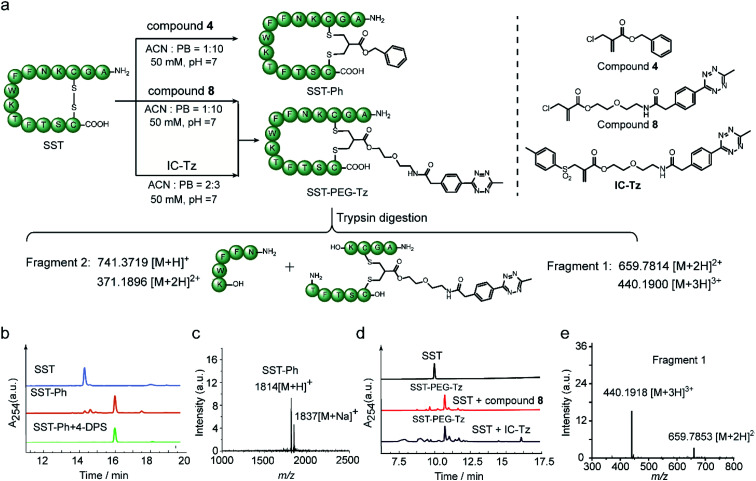
(a) Modification of SST with compound 4, compound 8, and an allyl sulfone reagent (IC-Tz). (b) HPLC trace of the crude reaction mixture between SST and compound 4 as well as the mixture of SST-Ph and 4-DPS; (c) MALDI-Tof-MS of the modified SST (SST-Ph) (calculated: 1814 [M + H]^+^, found: 1814 [M + H]^+^, 1837 [M + Na]^+^); (d) HPLC trace of the crude reaction mixture between SST and compound 8 (or IC-Tz); (e) ESI-MS of fragment 1 after trypsin digestion (calculated: 659.7814 [M + 2H]^2+^, 440.1900 [M + 3H]^3+^, found: 659.7853 [M + 2H]^2+^, 440.1918 [M + 3H]^3+^).

Next, the feasibility of 2-chloromethyl acrylate for disulfide bond modification was evaluated on both peptide and protein substrates. The cyclic peptide hormone somatostatin (SST), which plays a key role in regulating the endocrine system and contains an accessible disulfide bond in its sequence,^39^ was selected as a model peptide (Fig. 6a). The disulfide bond in SST was first reduced by two equivalents of tris(2-carboxyethyl)phosphine (TCEP) to generate the two free thiol groups in ACN/PB mixture (1 : 10) at pH 7, followed by incubation with 1.1 equivalents of compound 4 for overnight in one-pot. HPLC of the crude reaction mixture revealed good modification efficiency (91% based on HPLC quantification) (Fig. 6b). The isolated SST-Ph conjugate was also characterized by MALDI-Tof-MS showing successful functionalization (Fig. 6c). SST-Ph was further incubated with TCEP before the subsequent addition of the thiol-reactive reagent, 4,4′-dithiodipyridine (4-DPS). HPLC analysis showed that SST-Ph remained intact without any observation of side reactions occurring with 4-DPS. Native SST was used as a control and the LC showed that a new peak was formed when 4-DPS and TCEP were used (Fig. S32†). These results taken together indicated the complete modification at the disulfide site (Fig. 6b). Since the 2-chloromethyl acrylate compounds do not contain aromatic groups in their scaffold, they have a rather low log *P*_o/w_ and thus provide better water-solubility than the reagents that contain phenyl groups, such as allyl sulfone reagents. This is particularly advantageous when modifying some therapeutic relevant proteins, which will suffer from aggregation issues if a large amount of organic solvent is needed during the modification process. Therefore, the disulfide modification efficiency of SST with 2-chloromethyl acrylate and allyl sulfone reagents was evaluated and compared with using compound 8 and an allyl sulfone reagent (denoted as “IC-tetrazine”, which was developed by our group before^22^) (Fig. 6a). Disulfide modification of SST with IC-tetrazine required 40% ACN for solubilization, whereas less than 10% of ACN was needed to dissolve compound 8. More importantly, the modification efficiency of compound 8 was considerably higher (83% based on the quantification of HPLC peak for the reaction mixture) compared to IC-tetrazine (67%) (Fig. 6e). For definitive confirmation of the modification site, SST-PEG-Tz was selected for LC-MS/MS analysis. After trypsin digestion, a fragment with *m*/*z* at 659.7814 [M + 2H]^2+^ was observed corresponding to fragment 1 (Fig. 6e). The expected modified sequence was detected by MS/MS, thus confirming that the modification occurred at the disulfide site (Tables S2–S5 and Fig. S40–S44†). Besides SST, another therapeutic relevant cyclic peptide octreotide, an analog of somatostatin with a longer biological half-life that is often applied in cancer diagnostics,^40^ was also successfully functionalized with a coumarin motif under the similar reaction conditions mentioned above (Scheme S11†). The MALDI-Tof-MS data confirmed the successful functionalization with a peak at 1364 corresponding to [M + H]^+^ (Fig. S46†).

**Fig. 7:**
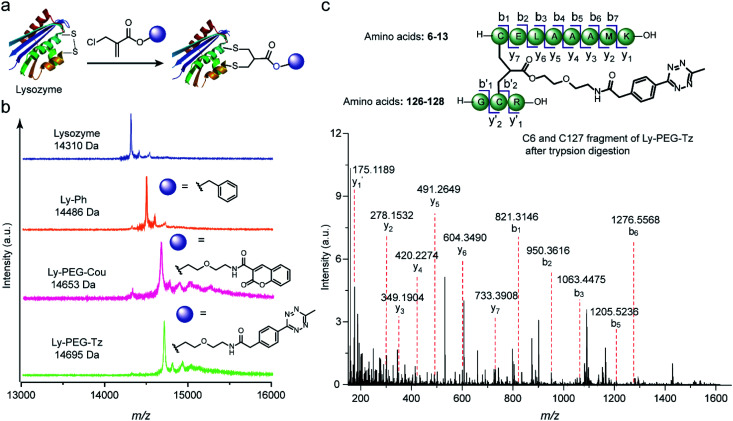
(a) Site-selective disulfide modification of lysozyme with different functionalities. (b) MALDI-Tof-MS of modified lysozyme with different functionalities: Ly-Ph (calculated: 14 485 [M + H]^+^, found: 14 486 [M + H]^+^), Ly-PEG-Cou (calculated: 14 654 [M + H]^+^, found: 14 653 [M + H]^+^), Ly-PEG-Tz (calculated: 14 694 [M + H]^+^, found: 14 695 [M + H]^+^) (c) MS/MS analysis of the C6 and C127 fragment of Ly-PEG-Tz after trypsin digestion. The expected y and b ions and the zoom-in spectra of the respective fragment ions are given in Section 8.2 in ESI.†

Subsequently, this new disulfide modification strategy was also evaluated on a more complex substrate, the protein enzyme lysozyme (from hen egg white), in which the disulfide at C6–C127 is predicted to be solvent-accessible among the four available disulfide bonds.^20,41^ To test the applicability of the 2-chloromethyl acrylate compounds for disulfide modification, different functionalities were incorporated into lysozyme, such as a phenyl group, a fluorescent dye (coumarin), or a bioorthogonal tag (tetrazine group) (Fig. 7a). After adding 1.2 equivalents of TCEP, the 2-chloromethyl acrylate derivatives were also added in one pot, and the reaction mixture was incubated at 50 mM PB (pH 7) overnight. Some precipitates were observed after incubation overnight, presumably due to the aggregation of reduced lysozyme despite the mild conditions employed.^42^ Thereafter, the modified lysozyme derivatives (Ly-Ph, Ly-PEG-Cou, and Ly-PEG-Tz) were purified by using Hi Trap hydrophobic interaction column with the isolated yields of 28%, 22%, and 24%, respectively. MALDI-Tof-MS data of the three modified lysozymes derivatives confirmed their successful functionalization (Fig. 7b). The yields are higher than our previous report where lysozyme was modified with allyl sulfone reagent (19% isolated yield, Table S12†) and comparable to that where cysteine in human serum albumin was modified with maleimide (∼30%).^20,43,44^ Notably, around 25–30% of native lysozyme was recovered after the purification, which can be recycled for modification. In order to identify the modification site, Ly-PEG-Tz was analyzed by LC-MS/MS. After trypsin digestion, only the fragment containing C6–C127 disulfide bonds was observed with an addition of PEG-Tz functionality (*m*/*z* 1553.7021 [M + H]^+^, Fig. S49–S54†). The fragments showing modification at other disulfide bonds were not observed in the analysis (Table S7 in ESI†). Further MS/MS analysis confirms the expected sequence and demonstrates the site-selective modification at the disulfide site (Fig. 7c). The expected y and b ions and the zoom-in spectra of the respective fragment ions are shown in Section 8.2 in ESI.†

**Fig. 8:**
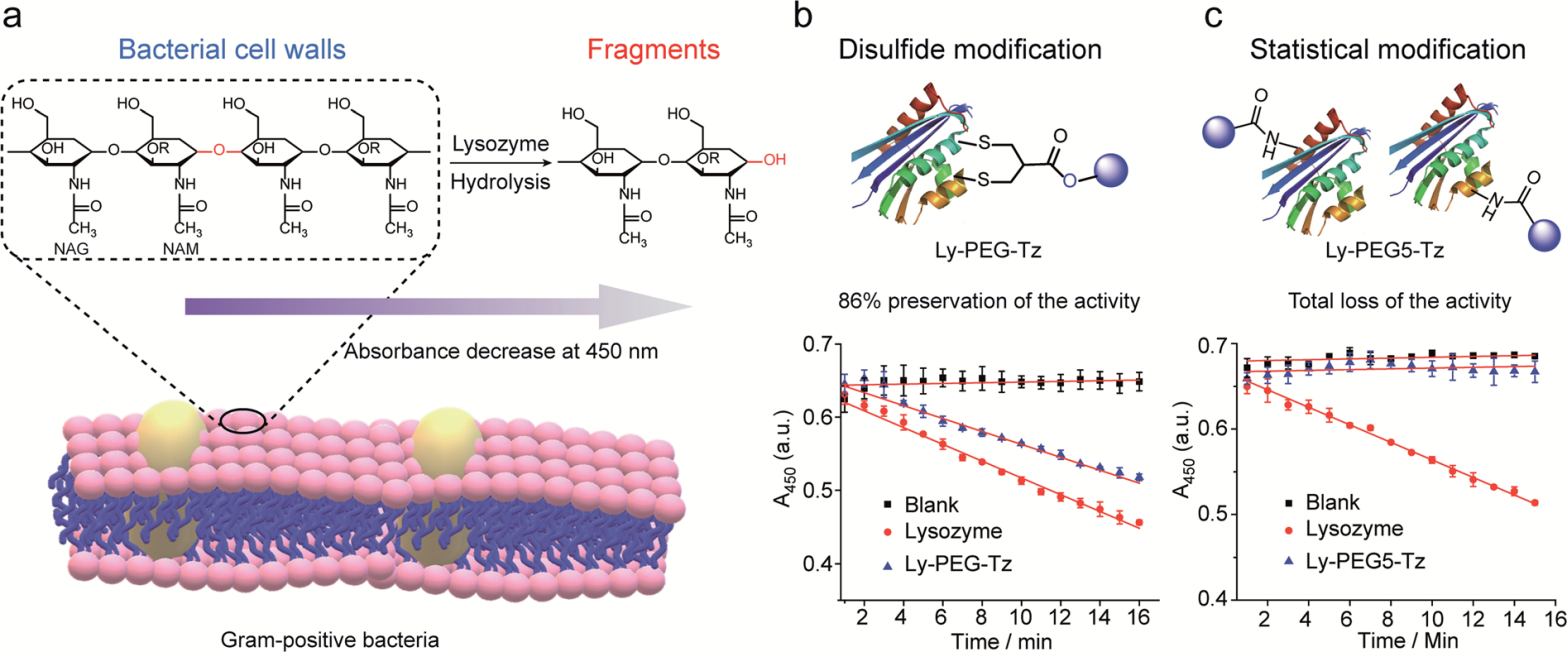
(a) The hydrolysis of 1,4-beta-linkages between *N*-acetyl-d-glucosamine (NAG) and *N*-acetylmuramic acid (NAM) residues in peptidoglycans of Gram-positive bacterial cell walls. The absorbance of the cell suspension at 450 nm was monitored to evaluate their catalytic activity and the rate of absorbance decrease was proportional to its activity. (b) Compared to native lysozyme (red), disulfide-modified lysozyme retained 86% activity (blue). The calculation details are shown in Section 8.3 of the ESI.† (c) Statistical modified lysozyme based on NHS ester chemistry resulted in total loss of the catalytic activity. The calculation details were shown in ESI.†

Lysozyme is an antimicrobial enzyme that is capable of hydrolyzing the 1,4-beta-linkages in the peptidoglycan of Gram-positive bacterial cell walls, thus leading to the lysis of bacteria (Fig. 8a). Therefore, the catalytic activity of modified lysozyme was assessed by investigation of the absorbance change at 450 nm of *Micrococcus lysodeikticus* lyophilized cell suspensions over time, where the activity of the modified lysozyme is proportional to their capability to hydrolyze the bacterial cell walls.^45^ In comparison to native lysozyme, the disulfide-modified lysozyme Ly-PEG-Tz retained 86% of its activity (Fig. 8b, calculation details shown in Section 8.3 in ESI†). In contrast, statistical modification of lysine residues of lysozyme using tetrazine *N*-hydroxysuccinimide compounds (Scheme S13†), which gave a heterogeneous mixture according to the MS data (Fig. S67†), resulted in total loss of its catalytic activity (Fig. 8c, calculation details shown in Section 8.3 in ESI†). Hence, disulfide modification of proteins with 2-chloromethyl acrylate compounds represents an attractive approach to functionalize enzymatic proteins at distinct sites to preserve their catalytic activity.

## 3. Conclusion

In conclusion, we report that single atom substitution in 2-chloromethyl acryl reagents can achieve selective protein modification at cysteine or disulfide sites on demand. The reactivity profile of the prepared bioconjugation reagents can be customized by simply selecting different end-group functionalities (either amino or hydroxyl groups) to obtain the respective 2-chloromethyl acrylamide and acrylate compounds. Notably, the synthesis of the reported 2-chloromethyl acrylamide and acrylate compounds proceeds *via* a simple and easily implemented one-pot reaction based on easily accessible starting materials. We anticipate that the synthetic approach presented herein can be easily adapted in any laboratory for a broader scientific community.

Excellent labeling efficiency and high chemoselectivity of the 2-chloromethyl acrylamide compounds were demonstrated by the chemoselective modification of cysteine residues in several model peptides as well as proteins. In contrast, 2-chloromethyl acrylate regents allow modification of disulfide-containing peptides and proteins, such as SST, octreotide, and lysozyme. In addition, our new approach could offer the possibility for the dual modification of proteins by capitalizing on the reactivity difference of the 2-chloromethyl acrylamide and acrylate compounds. In this way, one could envision protein dual functionalization at cysteine residues and disulfide bonds can be achieved in a stepwise fashion within one system. We believe that the strategy presented herein offers an entirely new and elegant chemical approach to chemists and biologists to greatly enrich the currently available methodology toolbox for cysteine and disulfide modification. In this way, such progressive technologies will provide easy access to the broader scientific community in the design and preparation of advanced protein conjugates for various biological, biophysical, and medicinal applications.

## Author contributions

T. W. conceptualized and supervised the project; S. L. K. supervised the project. L. X. designed and performed the experiments. M. J. S. A. S. and P. M. P. G. contributed with MS studies to elucidate the structure of several bioconjugates present in this manuscript. L. X. wrote the original draft of the paper; M. J. S. A. S., P. M. P. G., S. L. K. and T. W. reviewed and edited the paper.

## Conflicts of interest

The authors declare no conflict of interest.

## Supplementary Material

SC-012-D1SC03250J-s001

## Data Availability

Supporting data for this article is presented in the ESI.†
