# Supplementary material for: Chemoselective cysteine or disulfide modification via single atom substitution in chloromethyl acryl reagents
Source: Chem Sci. 2021 Sep 9;12(40):13321–30. doi: 10.1039/d1sc03250j (PMC8528048; doi:10.1039/d1sc03250j)
Supplement: SC-012-D1SC03250J-s001 [file SC-012-D1SC03250J-s001.pdf]

Electronic Supplementary Material (ESI) for Chemical Science.
This journal is © The Royal Society of Chemistry 2021

Supporting Information

Chemoselective cysteine or disulfide modification via single atom substitution in chloromethyl acryl reagents

Lujuan Xu[a,b], Maria J. S. A. Silva[c], Pedro M. P. Gois[c], Seah Ling Kuan[a,b]*, Tanja Weil[a,b]*

[a]Max-Planck Institute for Polymer Research, Ackermannweg 10, 55128 Mainz (Germany)

[b]Institute of Inorganic Chemistry I, Ulm University Albert-Einstein-Allee 11, 89081, Ulm (Germany)

[c] Research Institute for Medicines (iMed.ULisboa), Faculty of Pharmacy, Universidade de Lisboa, 1649-003 Lisbon (Portugal)

* Corresponding authors

1. Materials and methods

1.1 Materials

Unless otherwise stated, all solvents and reagents are purchased from commercial sources (Merck, Sigma Aldrich) and are used directly without further purification. High-performance liquid chromatography (HPLC) was performed using acetonitrile (ACN, HPLC grade) and water (obtained from a Millipore purification system), both containing 0.1 % trifluoroacetic acid (TFA). The reactions were monitored by thin-layer chromatography (TLC) with Macherey-Nagel Alugram Sil G/UV254 plates at 254 nm or with proper stains ($KMnO_4$ solutions). Flash column chromatography is carried out with silica gel (0.04 mm–0.063 mm, 60 Å).

1.2 Methods and Instruments

1.2.1 Nuclear Magnetic Resonance Spectroscopy (NMR)

The NMR spectra are recorded using Bruker Avance 300 NMR fourier transform spectrometer using $CDCl_3$, CD_2Cl_2 or CD_3OD. The chemical shifts (δ) were reported as parts per million (ppm) referenced with respect to the residual solvent peaks.

1.2.2 High-Performance Liquid Chromatography (HPLC)

Preparative HPLC was performed using Shimadzu LC-20AP system. Either Atlantis T3 Prep OBDTM 5 µm, 19 × 150 mm column (with a flowrate of 10 mL/min) or a Phenomenex Gemini 5 µm NX-C18 110 Å 150 × 30 mm (with a flowrate of 25 mL/min) was used. The gradient started with 5% ACN, which was linearly increased to 100% ACN within 20 minutes.

Analytical HPLC was performed using Shimadzu LC-20AT system. Atlantis T3 column (4.6 × 100 mm, 5 µm) with a flowrate of 4 mL/min was used. The gradient started with 5% ACN, which was increased to 100% ACN within 20 minutes or 26 minutes depending on the compounds.

For both preparative and analytical HPLC, ACN and water, which contains 0.1% TFA, were used as eluting solvent. The absorbance was recorded at 190 nm, 214 nm, 254 nm and 520 nm. The software LabSolutions by Shimadzu and Powerpoint were used to process all HPLC spectra.

1.2.3 Liquid Chromatography - Mass Spectrometry (LC-MS)

LC-MS(ESI) was measured using Shimadzu LCMS2020 with a Kinetex 2.6 μm EVO C18 100 Å LC 50 × 2.1 mm column, an electrospray ionization source, a SPD-20A UV-Vis detector. MilliQ water and ACN, both of which contain 0.1% formic acid, were used as the eluting solvents for all the measurements. The solvent gradients start with 5% ACN and 95% water, reach 100% ACN at 16 minute, and then go back to 5% ACN and 95% water at 20 minute. Data were processed with LabSolutions provided by Shimadzu.

1.2.4 Matrix-Assisted Laser Desorption/Ionisation - Time of Flight Mass Spectrometry (MALDI-Tof-MS)

All the MALDI-Tof-MS spectra were obtained from rapifleX MALDI-TOF/TOF from Bruker. Protein samples were mixed with a saturated solution with sinapinic acid and peptide samples were mixed with α-cyano-4-hydroxycinnamic acid (CHCA) in water/ACN 1/1 + 0.1% TFA. Data processing was performed in mMass.

1.2.5 Circular Dichroism Spectroscopy (CD)

Circular dichroism was measured at room temperature from 260 to 190 nm (a bandwith of 1 nm) with the sample concentration of 0.1 mg/mL in PBS buffer (pH 7.4). The data pitch was set to 0.2 nm with the scanning speed 5 nm/min. Each sample was measured three times and the signal from the buffer blank was subtracted from the sample scan. The obtained data was processed in the software Spectra Analysis and CD Multivariate SSE by JASCO

2. Synthesis of the 2-chloromethyl acrylamide and acrylate compounds

General procedure for one-pot synthesis of compound **1** to **8**

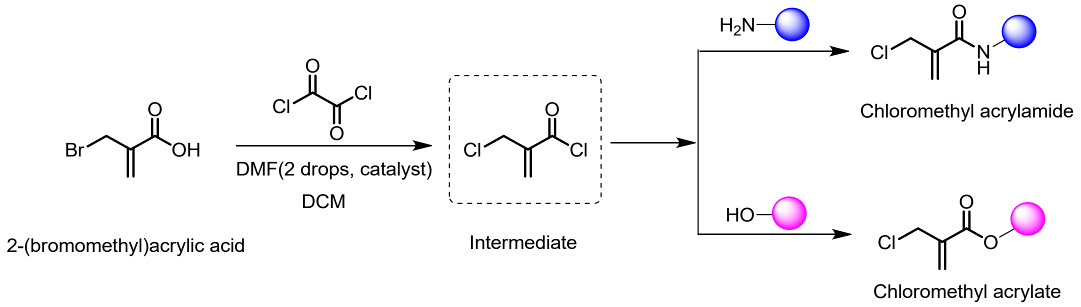

Scheme S1 The general synthetic scheme of the respective 2-chloromethyl acrylamide and acrylate derivatives with using the commercial available 2-(bromomethyl)acrylic acid as starting material.

Commercial available compound 2-(bromomethyl)acrylic acid (200 mg, 1.2 mmol, 1 eq) was added to a dry round-bottom with 10 mL anhydrous dichloromethane (DCM) under argon. Next, oxalyl chloride (1.2 g, 9.6 mmol, 8 eq) was added dropwise at 0°C. Then, several drops of anhydrous dimethylformamide (DMF) were also added via a syringe. The mixture was stirred under argon for 4 h. After that, the solvent was removed in vacuo and a white-to-yellow solid was obtained in the bottle. Without any further purification, the obtained intermediate, 2-(chloromethyl)acryloyl chloride, was dissolved in 10 mL anhydrous DCM and used directly for the next step. Thereafter, *N,N*- diisopropylethylamine (DIEA, 2 eq) and the amine or alcohol substrate (1 eq) were added to the acid chloride solution sequentially at 0°C. The resulting reaction mixture was stirred at room temperature overnight. After that, the mixture was washed with 1 M NaCl solution and the organic layer was dried over anhydrous $MgSO_4$. The solvent was evaporated under reduced pressure and the crude product was purified either with HPLC or flash column chromatography.

2.1 Synthesis of compound **1**

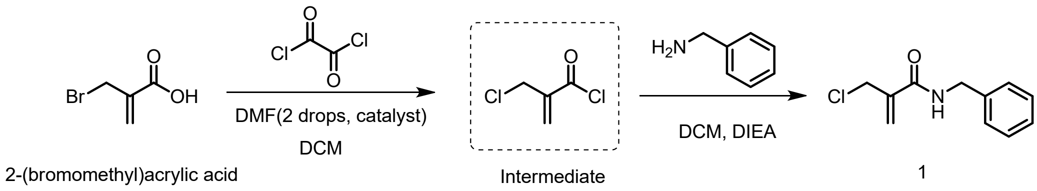

Compound **1** was synthesized according to the procedure described above. The crude product was purified with flash column chromatography (DCM:MeOH = 25:1) and compound **1** was obtained as white solid with 71% yield.

^{1}H NMR (300 MHz, $CDCl_3$) δ 7.44–7.28 (m, 5H), 5.86 (s, 1H), 5.70 (s, 1H), 4.54 (d, *J* = 5.7 Hz, 2H), 4.35 (s, 2H). ^{13}C NMR (75 MHz, $CDCl_3$) δ 166.04, 141.12, 137.84, 128.81, 127.78, 127.68, 122.05, 43.86,43.55

2.2 Synthesis of Compound **2**

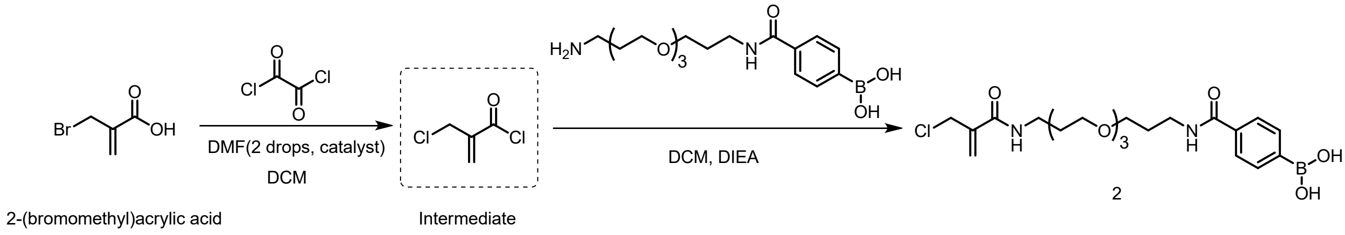

Amine-PEG3-B(OH) was synthesized based on the methods published from our group before.[1-3] Compound **2** was synthesized according to the general procedure described above and was purified with preparative HPLC using the general methods described above to offer a colorless liquid with 61% yield.

^{1}H NMR (300 MHz, $CDCl_3$) δ 7.81 (dd, *J* = 17.8, 7.6 Hz, 4H), 5.86 (s, 1H), 5.68 (s, 1H), 4.31 (s, 2H), 3.74 – 3.54 (m, 12H), 3.51 – 3.45 (m, 4H), 3.42 – 3.35 (m, 2H), 1.96 – 1.85 (m, 2H), 1.84 – 1.69 (m, 2H).^{13}C NMR (75 MHz, CD_2Cl_2) δ 168.75, 143.06, 134.69, 127.16, 122.70, 71.55, 71.53, 71.31, 71.25, 70.31, 70.04, 44.32, 38.75, 38.28, 30.42, 30.28. LC-MS: m/z = 471 $[M+H]^+$, 493 $[M+Na]^+$

2.3 Synthesis of Compound **3**

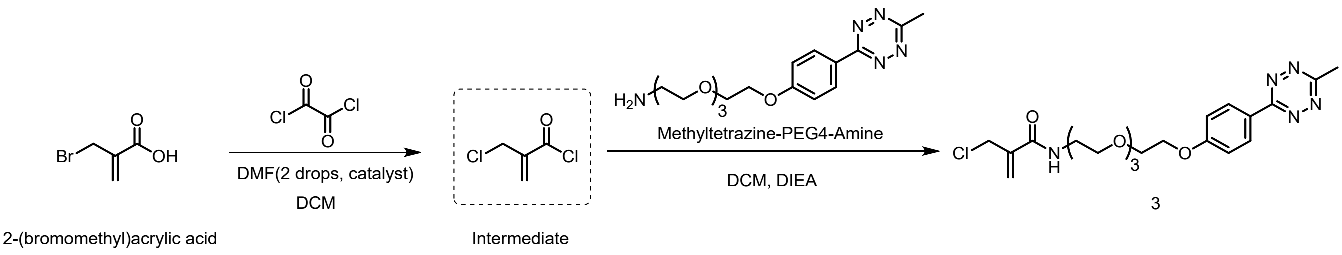

Compound **3** was synthesized according to the procedure described above. The crude product was purified with flash column chromatography (DCM:MeOH = 20:1) to obtain compound **3** as a pink to purple solid with 68% yield.

^{1}H NMR (300 MHz, $CDCl_3$) δ 8.53 (d, J = 8.8 Hz, 2H), 7.09 (d, J = 8.8 Hz, 2H), 5.86 (s, 1H), 5.67 (s, 1H), 4.31 (s, 2H), 4.24 (t, 2H), 3.90 (t, 2H), 3.79 – 3.58 (m, 11H), 3.57 – 3.51 (m, 2H), 3.06 (s, 3H).^{13}C NMR (75 MHz, $CDCl_3$) δ 166.63, 166.21, 163.71, 162.43, 141.16, 129.72, 124.41, 121.86, 115.25, 70.93, 70.61, 70.34, 69.62, 67.65, 53.43, 43.54, 39.56, 29.78, 21.06.LC-MS: m/z = 466 $[M+H]^+$, 488 $[M+Na]^+$

2.4 Synthesis of compound **4**

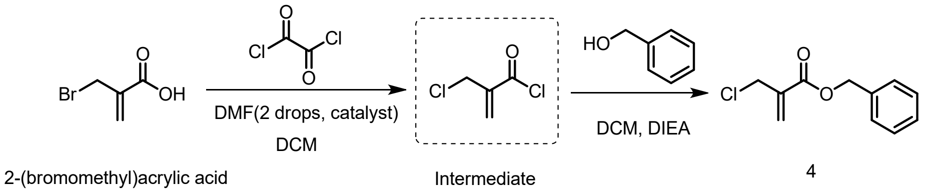

Compound **4** was synthesized according to the procedure described above. The crude product was purified with flash column chromatography (DCM:MeOH = 40:1) and compound **4** was obtained as white solid with 85% yield.

^{1}H NMR (300 MHz, $CDCl_3$) δ 7.49 – 7.29 (m, 5H), 6.44 (s, 1H), 6.01 (s, 1H), 5.25 (s, 2H), 4.31 (s, 2H).^{13}C NMR (75 MHz, $CDCl_3$) δ 164.88 (s), 136.81 (s), 135.56 (s), 129.05 (s), 128.63 (s), 128.38 (s), 128.17 (s), 66.97 (s), 42.53 (s).

2.5 Synthesis of compound **5**

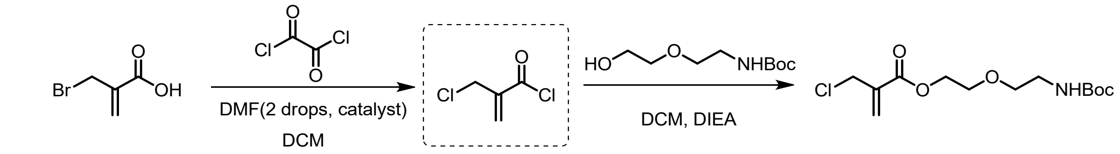

2-(bromomethyl)acrylic acid Intermediate 5

Compound **5** was synthesized according to the procedure described above. The crude product was purified with flash column chromatography (DCM:MeOH = 40:1) and compound **5** was obtained as white liquid with 67% yield.

^{1}H NMR (300 MHz, $CDCl_3$) δ 6.40 (s, 1H), 5.99 (s, 1H), 4.34 (t, 2H), 4.28 (s, 2H), 3.70 (t, 2H), 3.53 (t, J = 5.1 Hz, 2H), 3.36 – 3.24 (m, 2H), 1.42 (s, 10H). ^{13}C NMR (75 MHz, CD_2Cl_2) δ 165.17, 156.12, 137.26, 129.11, 79.24, 70.48, 69.05, 64.58, 43.07, 40.71, 28.48. LC-MS: m/z = 207 (Boc-deprotection under conditions used for LC-MS), 308 $[M+H]^+$, $[330 M+Na]^+$

2.6 Synthesis of compound **6**

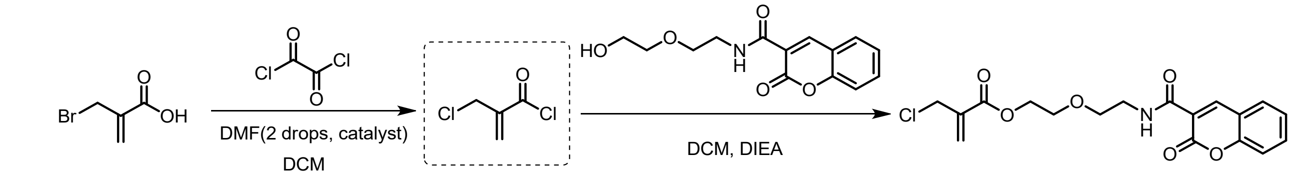

2-(bromomethyl)acrylic acid Intermediate 6

Compound **6** was synthesized according to the procedure described above. The crude product was purified with preparative HPLC via using the general method described above and compound **6** was obtained as yellow liquid with 58% yield.

^{1}H NMR (300 MHz, $CDCl_3$) δ 7.76 – 7.62 (m, 2H), 7.46 – 7.31 (m, 2H), 6.44 (s, 1H), 5.99 (s, 1H), 4.38 (t, 2H), 4.30 (s, 2H), 3.77 (t, 3H), 3.69 (s, 4H). ^{13}C NMR (75 MHz, $CDCl_3$) δ 164.97, 161.67, 161.24, 154.52, 148.31, 136.65, 134.05, 129.80, 129.11, 125.26, 118.68, 118.45, 116.65, 69.66, 68.85, 64.25, 42.57, 39.69. LC-MS: m/z = 380 $[M+H]^+$, 402 $[M+Na]^+$

2.7 Synthesis of compound **7**

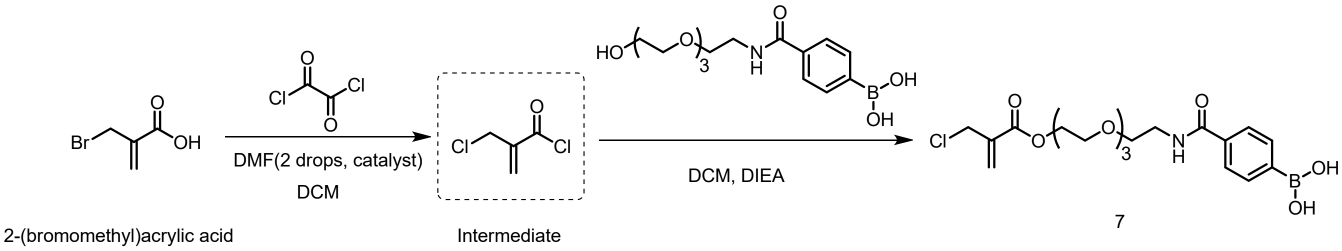

Compound **7** was synthesized according to the procedure described above.[1-3] The crude product was purified with preparative HPLC via using the general methods described above and compound **7** was obtained as white liquid with 64% yield.

^{1}H NMR (300 MHz, CD_2Cl_2) δ 7.89 (dd, *J* = 22.9, 7.5 Hz, 4H), 6.53 (s, 1H), 6.13 (s, 1H), 4.56 – 4.37 (m, 4H), 3.97 – 3.69 (m, 12H). ^{13}C NMR (75 MHz, CD_2Cl_2) δ 168.19, 165.36, 137.28, 134.60, 129.33, 126.47, 70.75, 70.61, 70.48, 70.38, 69.29, 64.67, 43.06, 40.26, 25.14. LC-MS: m/z = 444 $[M+H]^+$, 466 $[M+Na]^+$

2.8 Synthesis of compound **8**

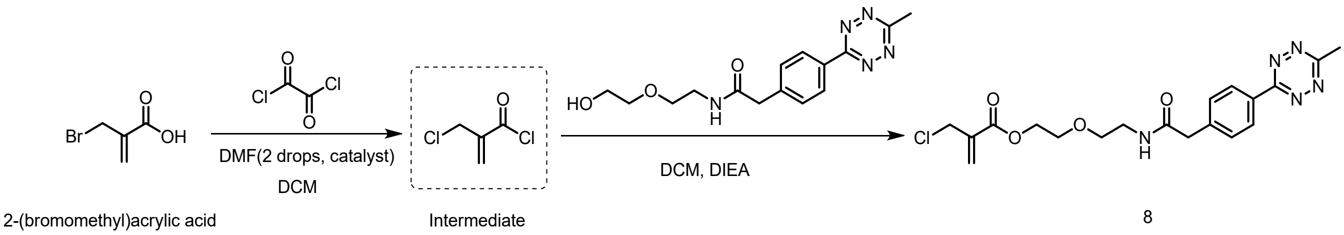

Compound **8** was synthesized according to the procedure described above The crude product was purified with flash column chromatography (DCM:MeOH = 25:1) and compound **8** was obtained as pink solid with 56% yield.

^{1}H NMR (300 MHz, $CDCl_3$) δ 8.46 (d, *J* = 8.1 Hz, 2H), 7.44 (d, *J* = 8.1 Hz, 2H), 6.28 (s, 1H), 5.91 (s, 1H), 4.31 – 4.14 (m, 4H), 3.60 (t, 2H), 3.56 (s, 2H), 3.47 (t, *J* = 5.0 Hz, 2H), 3.34 (dd, *J* = 10.2, 5.1 Hz, 2H), 2.99 (s, 1H). ^{13}C NMR (75 MHz, $CDCl_3$): δ 170.16, 167.30, 164.80, 163.85, 139.78, 136.72, 130.80, 130.31, 129.25, 128.38, 69.61, 68.84, 64.00, 43.53, 42.70, 39.50, 21.17.LC-MS: m/z = 420 $[M+H]^+$, 442 $[M+Na]^+$

2.9. Proposed mechanism

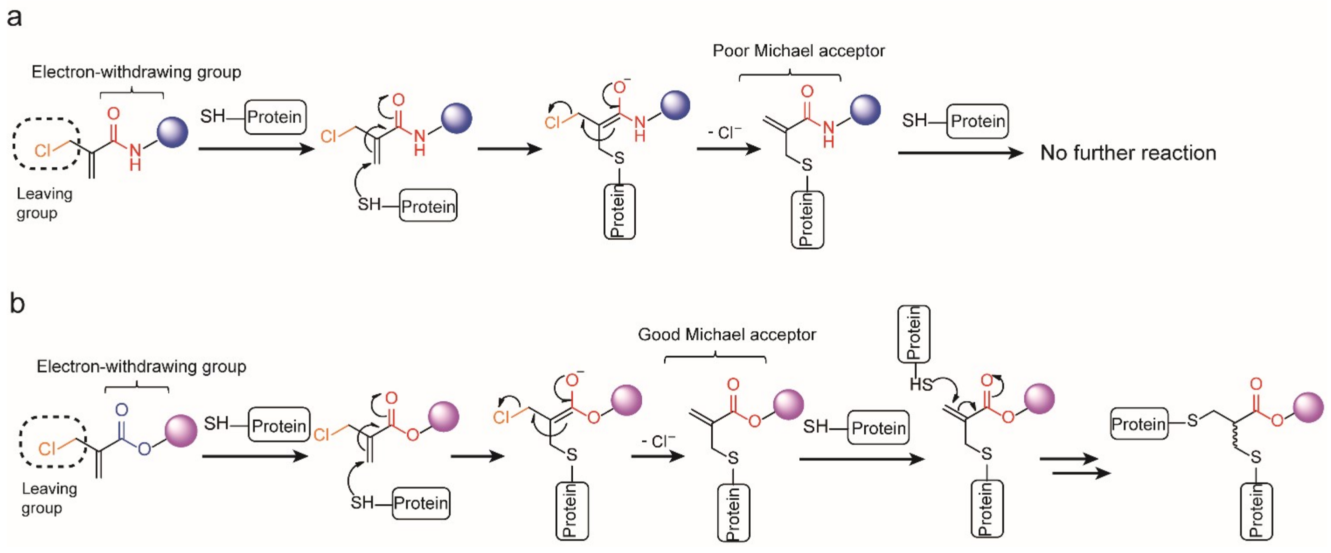

Fig. S1 Proposed mechanism for the reaction of 2-chloromethyl acryl derivatives and proteins which contain surface-exposed cysteines.

2.10 Log P (*n*-octanol to water) prediction of 2-chloromethyl acrylamide and acrylate derivative based on Chembiodraw 2016

Partition coefficient (P) refer to the ratio of the concentration of a compound in two immiscible solvents in equilibrium, such as *n*-octanol and water. The logarithm of the ratio is log P, which is often used in the literature and considered as a parameter to estimate how hydrophilic or hydrophobic a compound is. The smaller the log P is, the more hydrophilic a compound should be and vice versa. The log P of the 2-chloromethyl acrylamide and acrylate derivatives are summarized below. The log P data suggested that the 2-chloromethyl acrylamide and acrylate compounds \ designed in this paper have lower log P, thus they should be more hydrophilic than the corresponding carbonylacrylic and allyl sulfone derivatives developed by Bernardes[4] and our group[5] respectively. Higher hydrophilicity of the conjugation reagent should facilitate the subsequent reactions as less organic solvent is required to eliminate the risk for protein degradation or

aggregation.

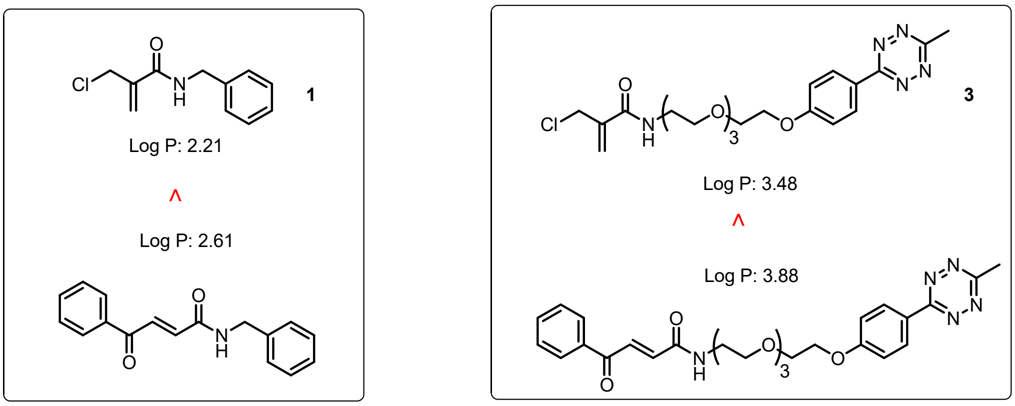

Fig. S2 The log P value of the 2-chloromethyl acrylamide derivatives (compound **1** and **3**) and the corresponding carbonylacrylic derivatives (developed by Bernardes and coworkers[4]) predicted from Chemdraw

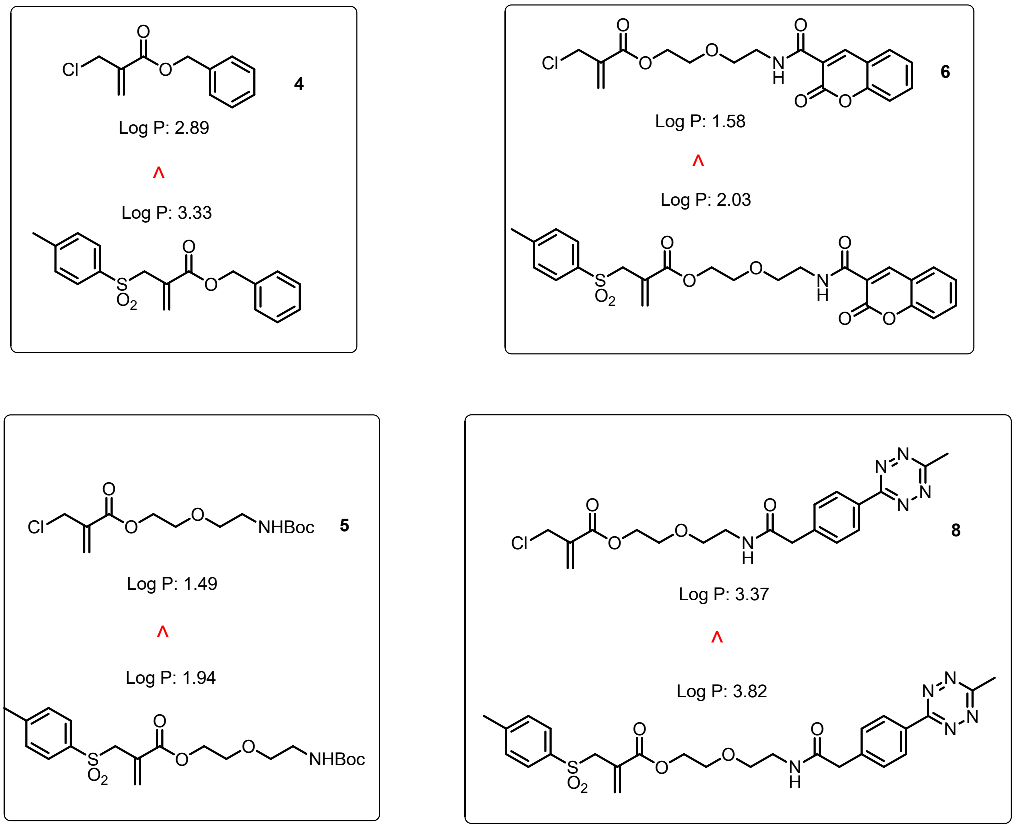

Fig. S3 The log P value of the 2-chloromethyl acrylate derivatives (compound **4**, **5**, **6** and **8**) and the corresponding allyl sulfone derivatives (developed by our group before[5]) predicted from Chemdraw.

2.10 Stability study of the 2-chloromethyl acrylamide and acrylate reagents

Compound **3** and compound **4** were selected for stability studies. In general, compound **3** (or **4**) was dissolved in 50 mM PB buffers at three different pH (pH 6, 7 or 8) with a concentration of 1 mg/mL. The mixed solutions at three different pH were incubated for 12, 24 and 36 hours. An aliquot of 10 μL of each solution was taken at each time point, mixed with 200 μL methanol and injected into HPLC to evaluate their stability.

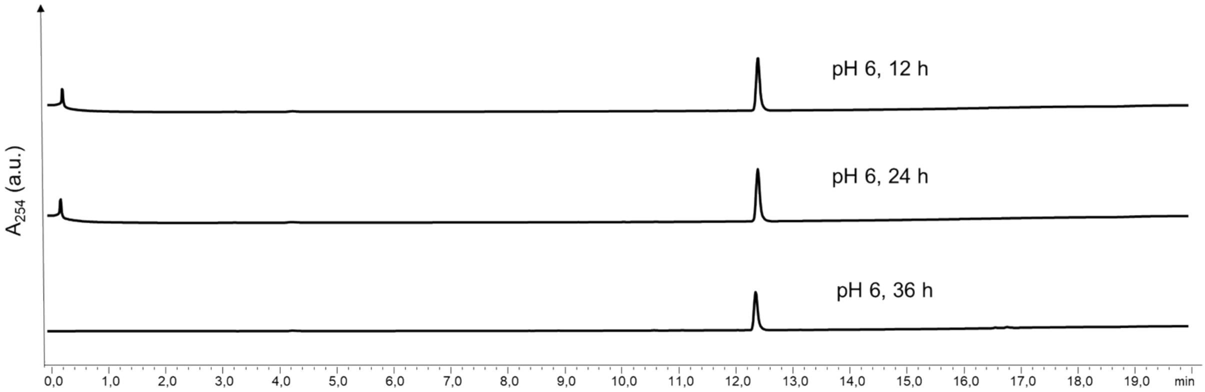

Fig. S4 Stability study of compound **3** at pH 6.

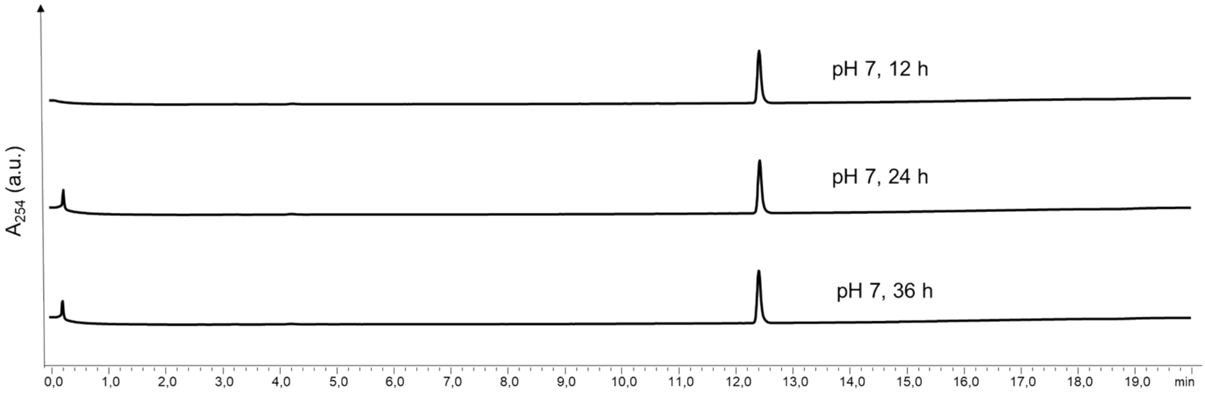

Fig. S5 Stability study of compound **3** at pH 7.

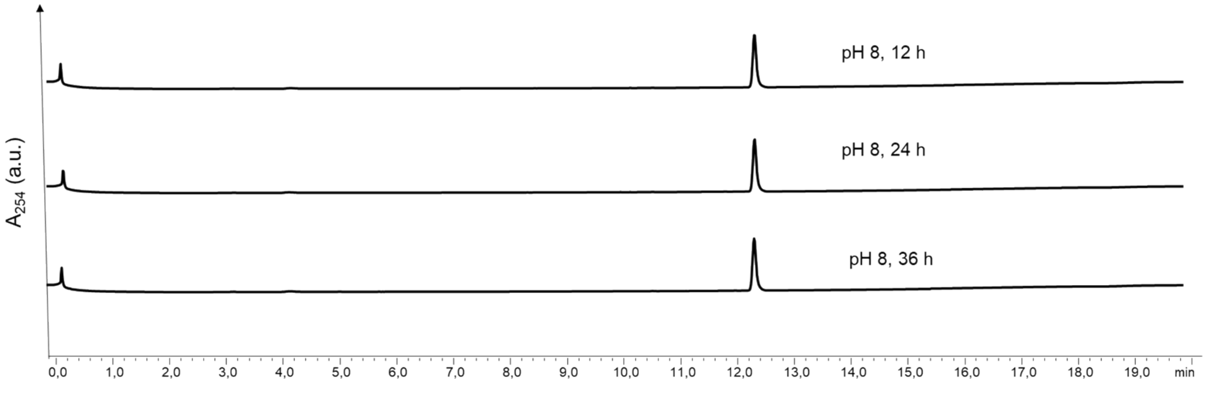

Fig. S6 Stability of compound **3** at pH 8.

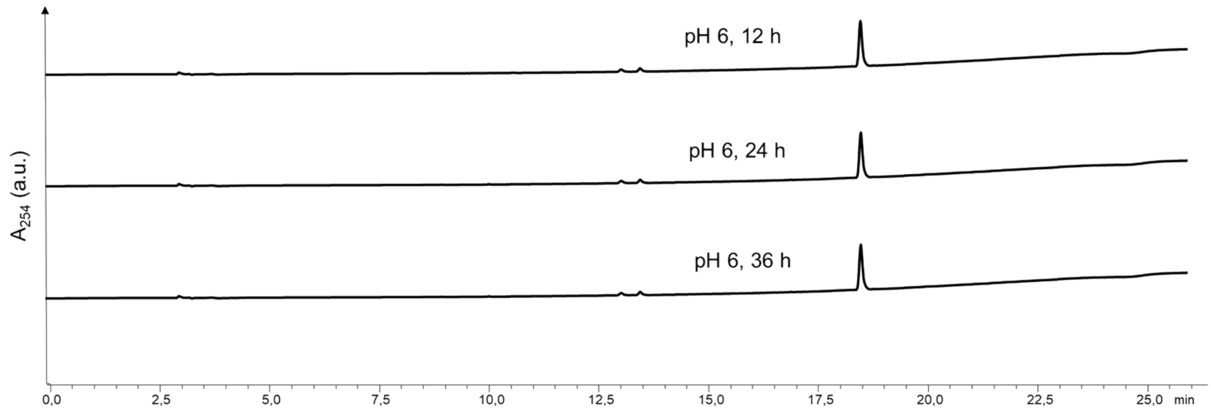

Fig. S7 Stability of compound **4** at pH 6.

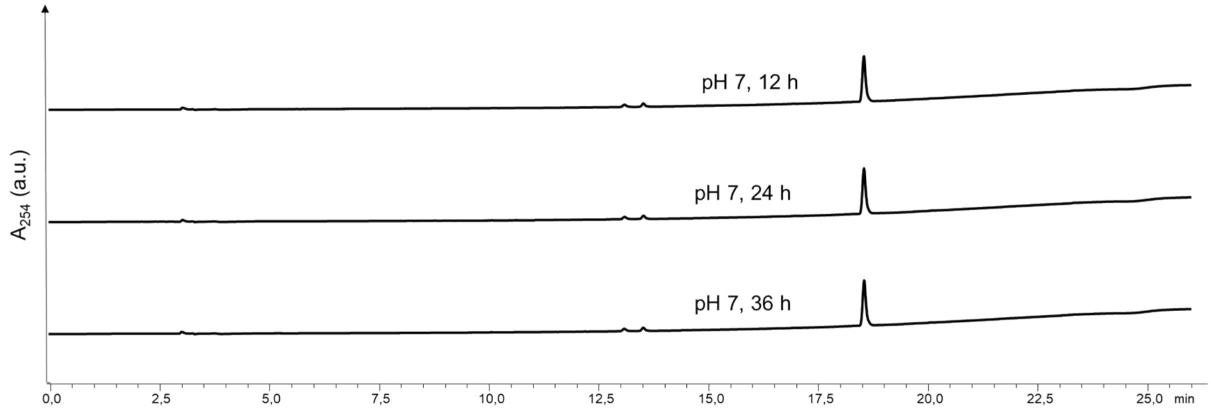

Fig. S8 Stability of compound **4** at pH 7.

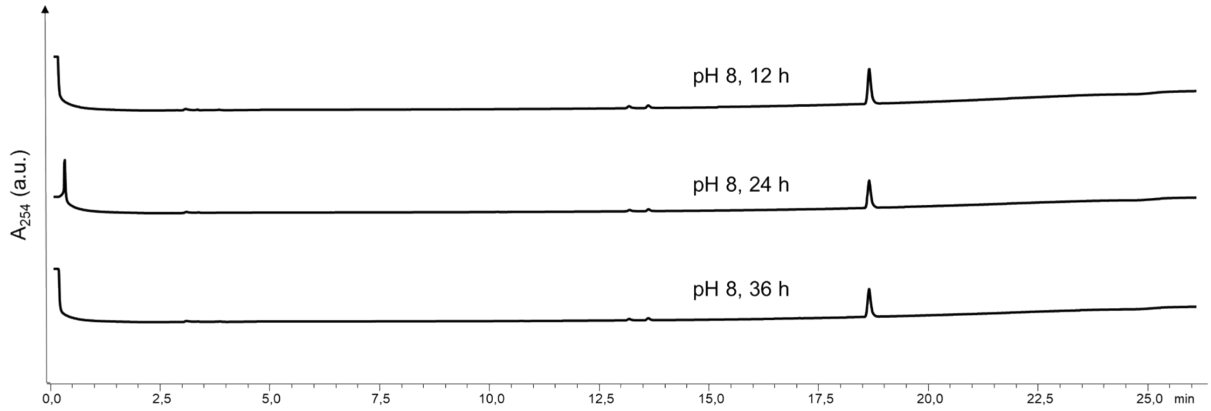

Fig. S9 Stability of compound **4** at pH 8.

2.11 Stability studies of the maleimide reagent

Maleimide compounds are the most commonly applied cysteine-modification reagent in the literature.

However, it is easy to undergo hydrolysis to obtain the ring-opening compound.[6] The hydrolytic pathway is shown below. The stability of the maleimide reagents was also evaluated at three different pH (pH 6, 7 and 8) and the results are shown below.

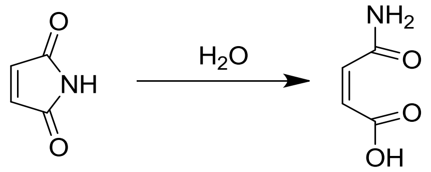

Scheme S2 General hydrolytic pathway of maleimide compounds.

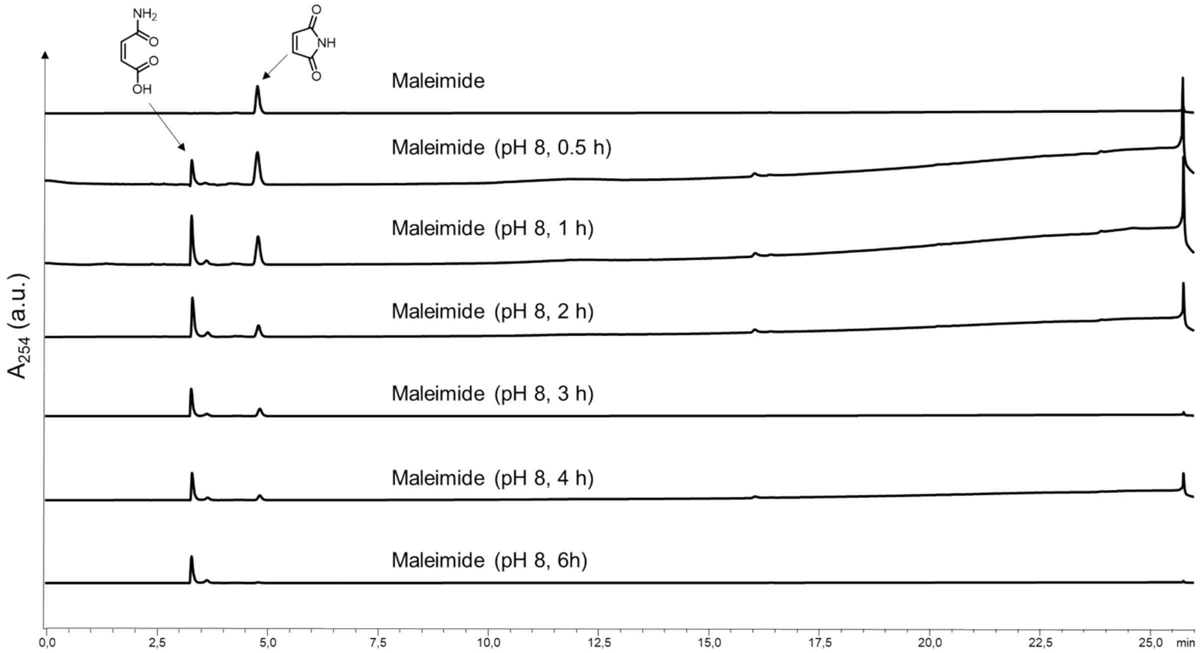

Fig. S10 Stability of maleimide reagent at pH 8.

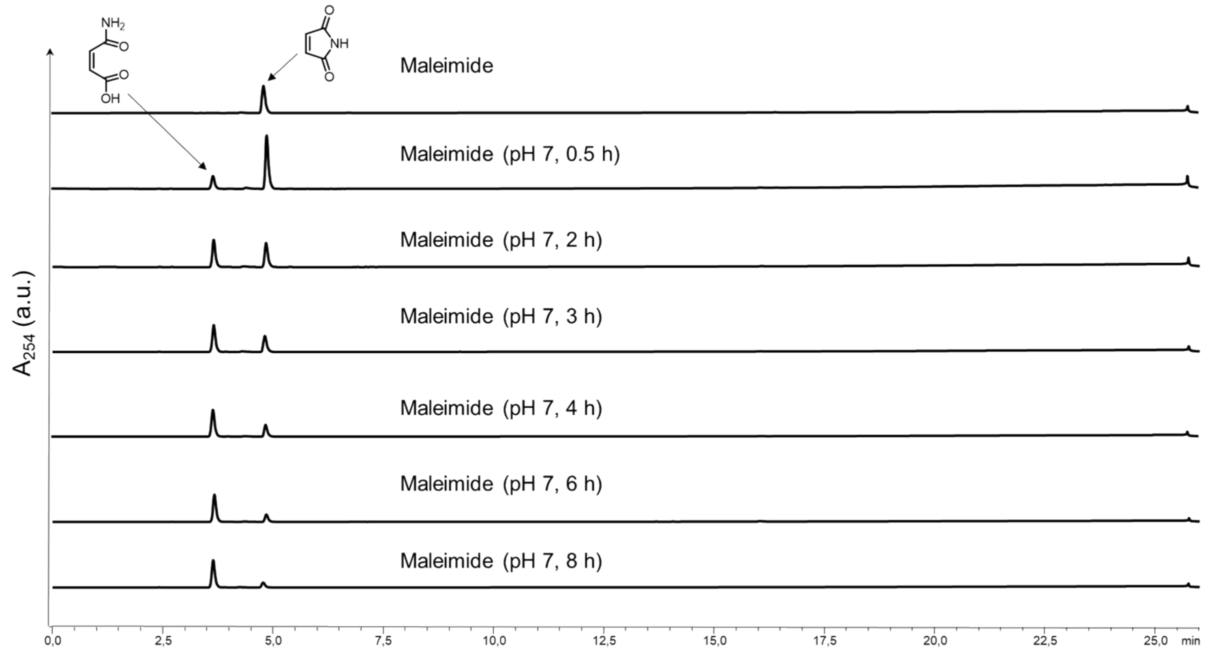

Fig. S11 Stability of maleimide reagents at pH 7.

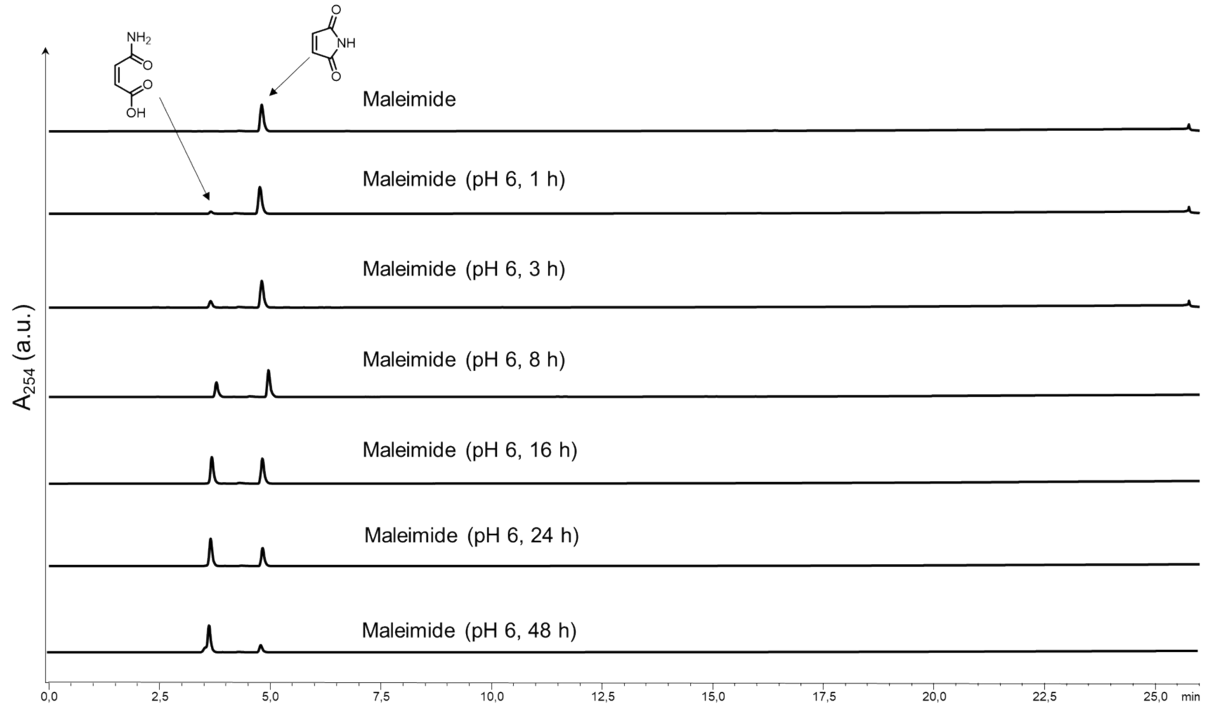

Fig. S12 Stability of maleimide reagent at pH 6.

3. Procedure for conducting model reactions to check chemoselectivity

To evaluate the chemoselectivity of the reagents towards thiol and amino groups, two model amino acids, Boc-Cys-OMe and Boc-Lys-OH, were selected to react with compound **1** or compound **4**. For the reaction between 2-chloromethyl acrylamide compound **1** and Boc-Cys-OMe (and Boc-Ly-OH), compound **1** (50 μg, 1 eq, 0.24 μmol) was mixed with Boc-Cys-OH (67 μg ,1.2 eq, 0.29 μmol, 50 mg/mL in DMF stock solution) and Boc-Ly-OH (70 μg, 1.2 eq, 0.29 μmol, 50 mg/mL in DMF stock solution) in ACN:PB (pH = 7) = 1:10. The mixture was incubated at room temperature for four hours. After that, 10 μL of the reaction mixture was mixed with 200 μL methanol and injected to LC-MS to check the product. By using the same procedure, the ratio of Boc-Cys-OMe was gradually increased to 2 eq, 4 eq, 6 eq, and 8 eq. After 4 h, the mixture was again diluted with methanol and injected to LC-MS to check the product.

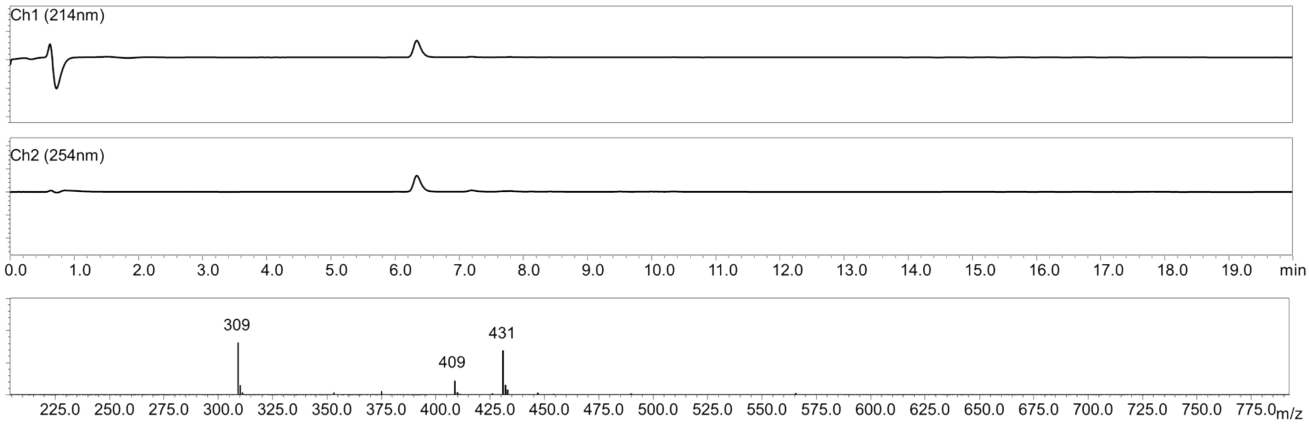

Fig. S13 LC-MS of compound **10** (calculated: 409 $[M+H]^+$, 431 $[M+Na]^+$, found: 409 $[M+H]^+$, 431 $[M+Na]^+$)

Reactivity studies of compound 10 with different nucleophiles

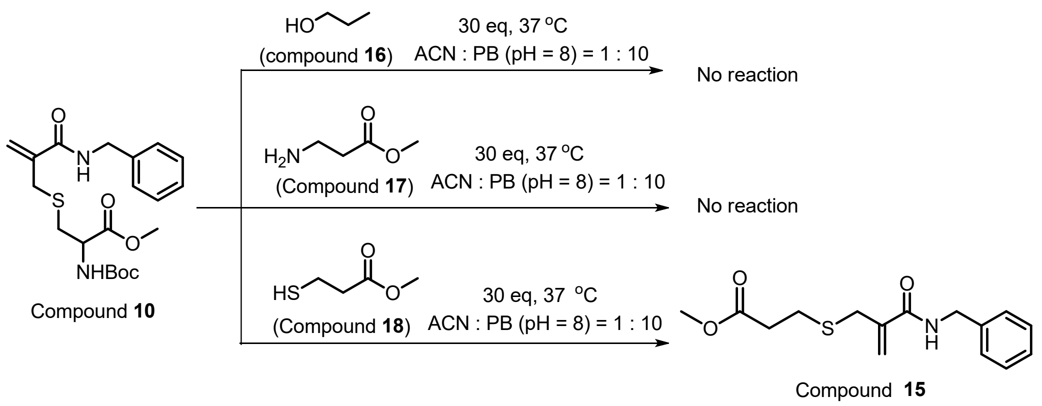

Scheme S3. Assessment of the reactivity of compound 10 with different nucleophiles (hydroxyl group, amino group and thiol group).

In order to assess the reactivity of compound **10** with different nucleophiles (hydroxyl group, amino group and thiol group), compound **10** (50 µg, 1 eq, 0.12 µmol) was incubated with a large excess (30 eqiv.) of compound **16**, **17** or **18** in ACN / PB (pH = 8) at 37 °C for overnight. No further reaction was observed between compound **10** and compound **16** or **17** on the LC-MS trace. However, compound **15** was formed after incubating compound **10** with compound **18** overnight at 37 °C, in which the first thiol-functionality was eliminated presumably due to an addition-elimination reaction. The reactions were monitored using LC and MS (Figure S14).

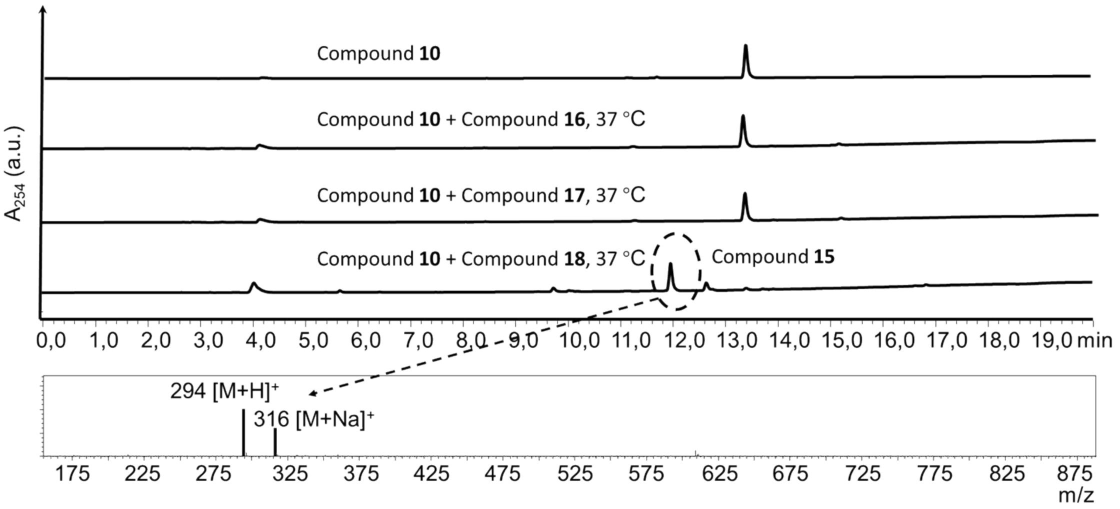

Fig. S14 HPLC analysis of the reaction between compound **10** and three different nucleophiles (hydroxyl, amino and thiol groups)

For the reaction between 2-chloromethyl acrylate compound **4** and Boc-Cys-OMe (and Boc-Ly-OH), compound **4** (50 µg, 1 eq, 0.24 µmol, 40 mg/mL in DMSO stock solution) was mixed with 67 µg ,1.2 eq, 0.29 µmol, 50 mg/mL in DMF stock solution) and Boc-Ly-OH (70 µg, 1.2 eq, 0.29 µmol, 50 mg/mL in DMF stock solution) in ACN:PB (pH = 7) = 2:3. After that, 10 µL of the reaction mixture was diluted with 200 µL methanol and injected to LC-MS to check the product. Following the same procedure, the ratio of Boc-Cys-OMe increased from 1.2 eq to 2 eq, 3 eq, 6 eq, and finally to 8 eq. For each reaction, 10 µL of reaction mixture was diluted with 200 µL methanol and LC-MS was used to evaluate the reaction progress.

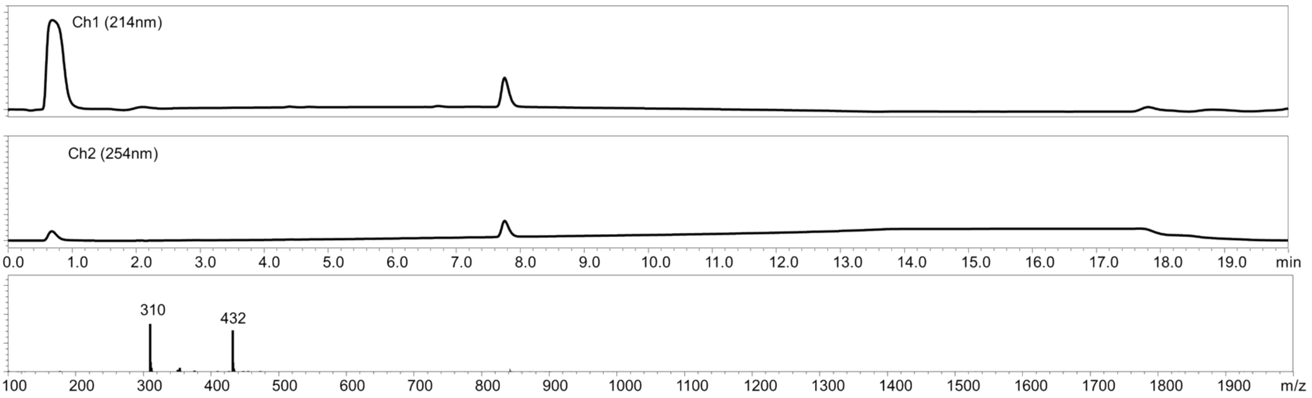

Fig. S15 LC-MS of compound **12** (calculated: 410 [M+H]+ , found: 432 [M+Na]$^+$, 310 [boc-deprotected product]$^+$)

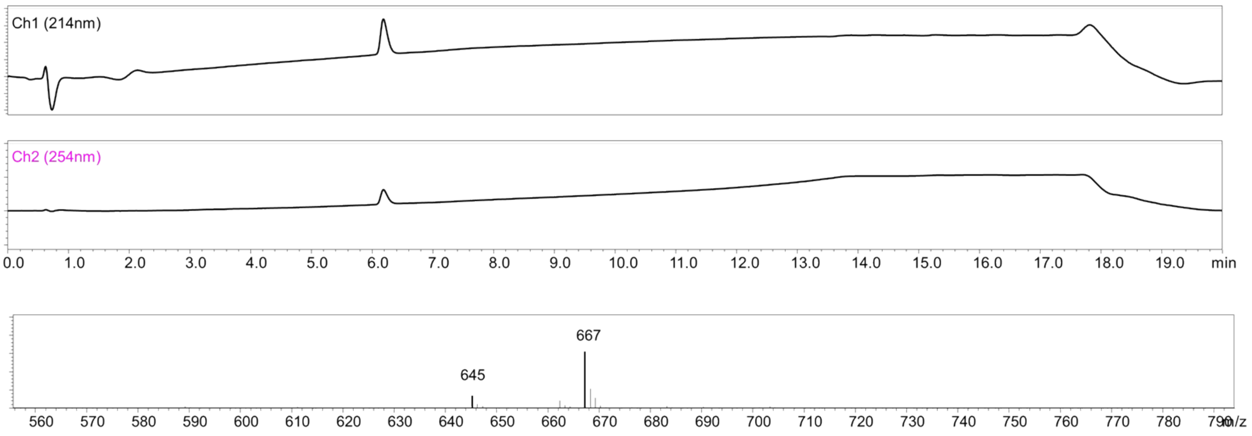

Fig. S16 LC-MS of compound **13** (calculated: 645 [M+H]$^+$, 667 [M+Na]$^+$, found: 645 [M+H]$^+$, 667 [M+Na]$^+$)

4. Kinetic study

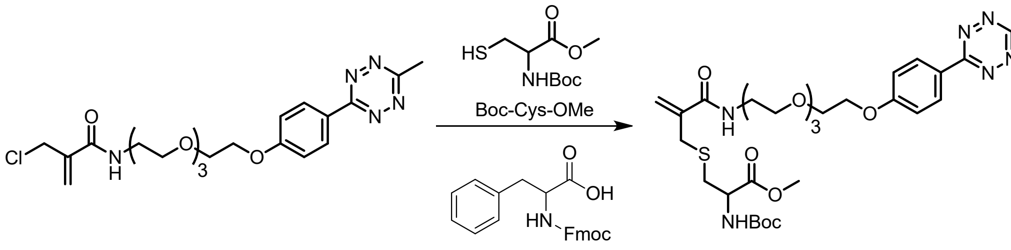

Compound 3 Fmoc-Phe-OH Compound 14

Scheme S4 Reaction scheme between compound **3** and Boc-Cys-OMe with Fmoc-Phe-OH as internal standard

For the kinetic study, compound **3** (1 mM, 20 mg/mL in DMF stock solution), Boc-Cys-OMe (1 mM, 50 mg/mL in DMF stock solution) and internal standard Fmoc-Phe-OH (0.2 mM, 40 mg/mL in DMF) were mixed in 200 μL ACN : PB (pH 7) mixture in 1.5 mL eppendorf. At certain time point (e.g. 15 min, 30 min, 60 min, 90 min, 120 min, 180 min, 240 min, 270 min, 360 min), 10 μL of reaction mixture was withdrawn from the eppendorf and the reaction was immediately quenched by 200 μL methanol which contained 10 μL trifluoroacetic acid. After quenching the reaction, the mixture was frozen before injecting to HPLC. HPLC data clearly indicated that the peak for compound **3** was gradually decreased while the peak for compound **14** increased. The quantification was obtained based on the integration of the absorbance peak of compound **3** and compound **14** in comparison to the internal standard Fmoc-Phe-OH, which correspond to the conversion during the reaction progress.

The rate constant of the reaction between compound **3** and Boc-Cys-OMe was obtained based on the second-order reaction kinetics. The second-order reaction equation: v = k × [A] [B]

If [A] = [B], $-\frac{d[A]}{dt} = k \times [A]^2$, so $\frac{d[A]}{[A]^2} = -k \times t$,

$$\int_{[A_0]}^{[A]} \frac{d[A]}{[A]^2} = -k \int_0^t dt$$

$$\frac{1}{[A]} = \frac{1}{[A_0]} + k\,t$$

[A]: real-time concentration of compound **3**, [B]: real-time concentration of Boc-Cys-OMe

[A_0]: initial concentration of compound **3** (1 mM), k: second-order rate constant.

Based on the HPLC data, $\frac{1}{[A]}$ over time was linearly-plotted which is shown in Fig. 3 and the slope is the second-order rate constant (1.17 ± 0.01 $M^{-1}s^{-1}$).

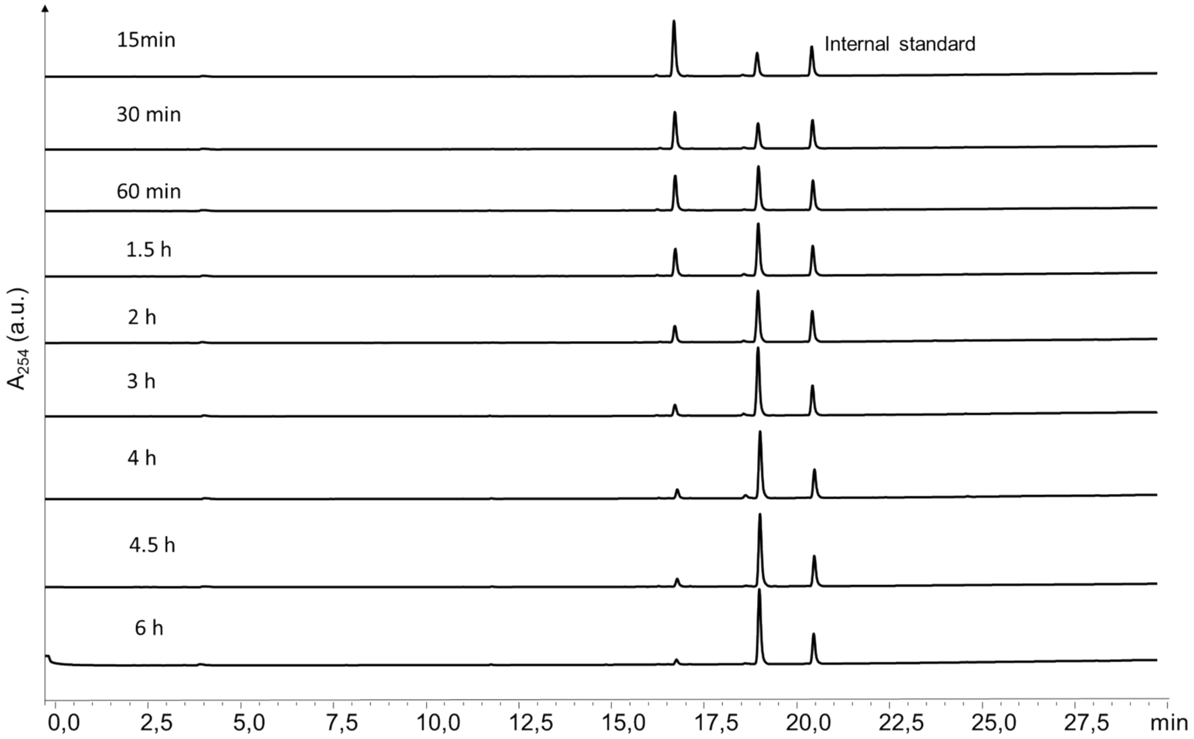

Fig. S17 HPLC trace of the reaction progress between compound **3** and Boc-Cys-OMe with Fmoc-Phe-OH as internal standard at different time points.

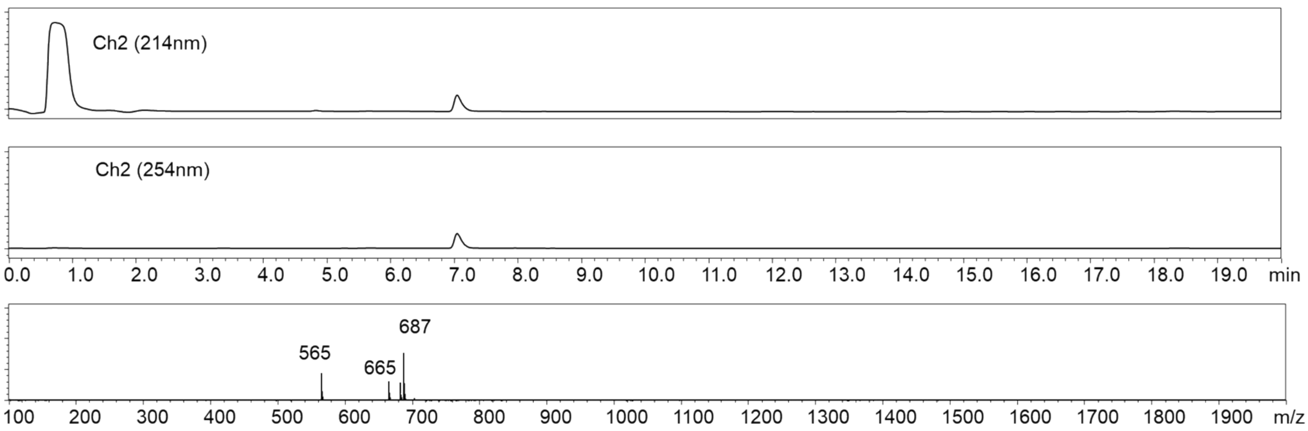

Fig. S18 LC-MS of compound **14**. (calculated: 664 $[M+H]^+$, found: 664 $[M+H]^+$. 687 $[M+Na]^+$, 565: Boc-deprotected product)

Reagents	Rate constant ($M^{-1}s^{-1}$)	Reference
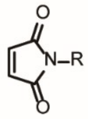 Maleimide	10 - 1000	*ACS Chem. Biol.*, **2015**, *10*, 1026−1033
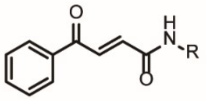 Carbonylacrylic reagents	10.9	*Nat. Comm.*, **2016**, *7*,13128
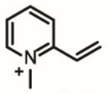 Quaternized vinyl pyridine	200	*Angew. Chem. Int. Ed.*, **2019**, *58*, 6640 –6644
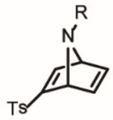 Azabicyclic vinyl sulfones	Not reported	*Chem. Sci.*, **2019**, *10*, 4515–4522
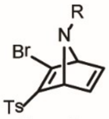 Azanorbornadiene bromovinyl sulfones	Not reported	*Angew. Chem. Int. Ed.*, **2020**, *132*, 6255 –6259
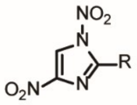 1,4-Dinitroimidazoles	Not reported	*Nat. Commun.* **2019**, *10*, 142
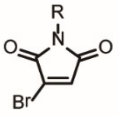 3-Bromo-5-methylene	Not reported	*Nat. Commun.*, **2020**, *11*, 1015
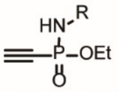 Ethynylphosphoamidate	0.62	*Angew. Chem. Int. Ed.*, **2019**, *58*,11625 –11630
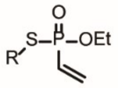 Vinylphosphonothiolate	0.0021	*J. Am. Chem. Soc.* **2020**, *142*, 9544–9552
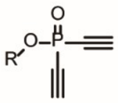 Diethynyl phosphinate	0.47	*Angew. Chem. Int. Ed.*, **2021**, *60*, 15359 –15364
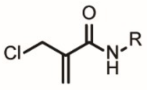 2-Chloromethyl acrylamide	1.17	This work

Table S1 A brief summary of the reaction rate of representative cysteine modification strategies developed in the literature and in this work.

5. Peptide modification with 2-chloromethyl acrylamide compounds

5.1 Cysteine modification of WSC02 peptide

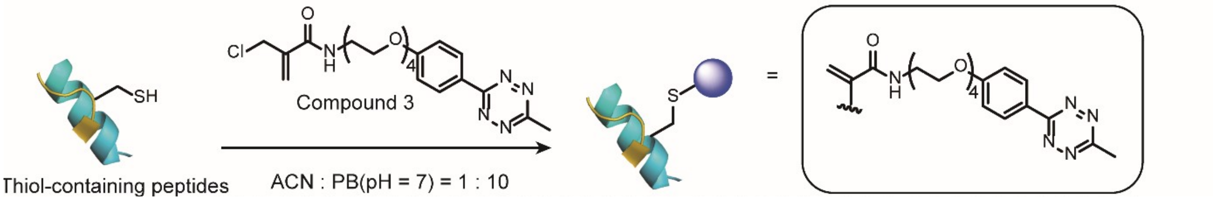

Scheme S5 Site-selective modification of different thiol-containing peptides with compound 3

WSC02 peptide (sequence: IVRWSKKVCQVS) was selected as model substrate for modification. WSCO2 peptide (1 mg, 1 eq, 0.71 μmol) was dissolved in 1 mL ACN: PB (pH = 7) = 1:10 mixture. Next, compound **3** (36 μg, 1.1 eq, 0.78 μmol, 20 mg/mL in DMF stock solution) was also added to the WSC02 peptide solution. The mixture was incubated at room temperature for 4 h. Next, 10 μL of mixture was diluted with 200 μL methanol and injected to LC-MS to check if any product was formed. The HPLC trace of the crude reaction mixture was shown in Fig. 4c showing excellent modification efficiency. Modified WSC02 peptide was purified by analytical HPLC and WSC02-PEG4-Tz was obtained as pink powder with the yield of 88%. The modified WSC02 peptide was also characterized with MALDI-Tof-MS with sinapinic acid as matrix showing the correct molecular weight at 1830 ($[M+H]^+$). The peak at 1761 in MALDI-Tof-MS spectra is the fragmentation product, which is shown below.

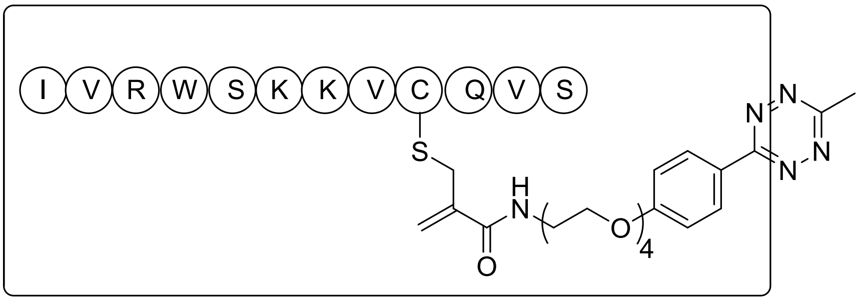

Fragmentation of WSC02-PEG4-Tz (1761)

Fig. S19 The chemical structure of the fragmentation part (in the frame) of WSC02-PEG4-Tz in MALDI-Tof-MS spectra.

4,4'-Dithiodipyridine is a disulfide-containing compound which is often used for thiol quantification.[7] It

reacts with thiols quantitatively in a thiol-disulfide exchange reaction. The reaction mechanism is shown in Scheme S5.

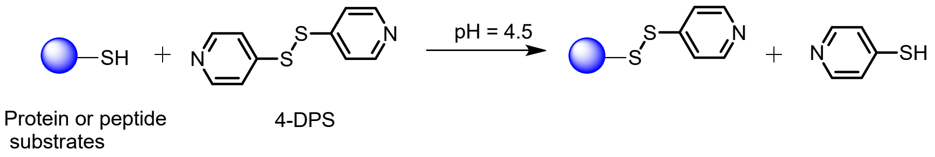

Scheme S6 Reaction mechanism of thiol-containing substrates with 4-DPS.

In order to check if the reaction is chemoselctive to thiol groups, the thiol group of native WSCO2 was firstly masked with 4-DPS. WSC02 peptide (100 μg, 1 eq, 0.071 μmol) was dissolved in 100 μL ACN : citrate buffer (pH = 4.5) = 1:10 mixture. 4-DPS (126 μg, 8 eq, 0.57 μmol) was added. The mixture was incubated at room temperature for 4 h. After that, the pH of the reaction mixture was adjusted to pH 7 and compound **3** (39.9 μg, 1.2 eq, 0.085 μmol) was added and the resultant mixture was incubated for another 4 hours. After that, 10 μL mixture was withdrawn and diluted with 200 μL methanol. LC-MS data indicated that there is no further reaction observed after the masking of the thiol groups (shown in Fig. 4c).

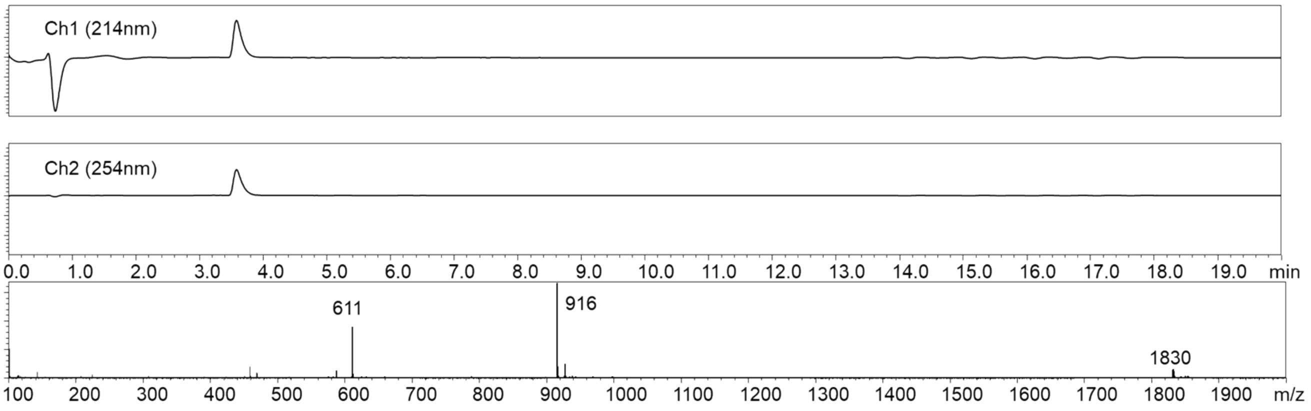

Fig. S20 LC-MS of the modified WSC02 peptide (WSC02-PEG4-Tz) (calculate: 1830 $[M+H]^+$, found: 1830 $[M+H]^+$. 916 $[M+2H]^{2+}$, 611 $[M+3H]^{3+}$)

5.2 Stability study of the tetrazine-modified WSC02 peptide (WSC02-PEG4-Tz).

It was reported that thiol-maleimide conjugates are prone to decompose via hydrolysis and/or retro-

Michael reaction.[6, 8] Therefore, the stability of WSC02-PEG4-Tz was evaluated at three different pH (pH 6, 7 and 8). As shown below, there is no obvious decomposition of WSC02-PEG4-Tz in the tested pH for 36 hours.

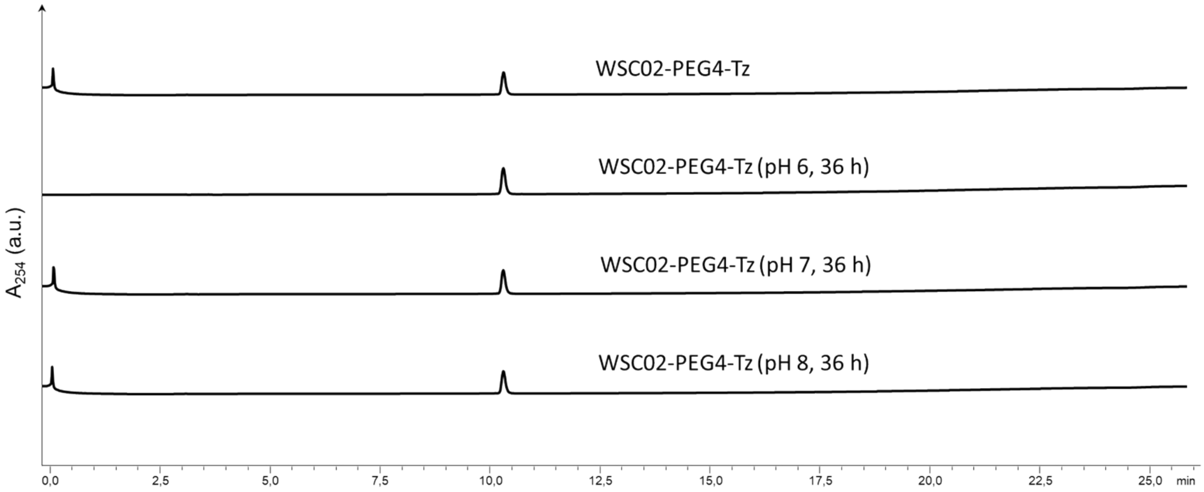

Fig. S21 Stability study of WSC02-PEG4-Tz at three different pH (pH 6, 7 and 8) for 36 hours.

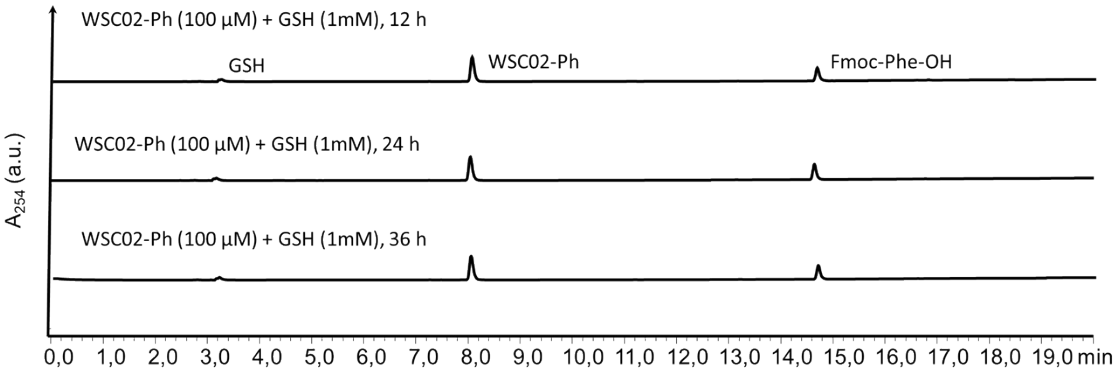

Fig. S22 Stability study of WSC02-Ph (100 µM) under 1 mM GSH for different time (12 h, 24 h, and 36 h).

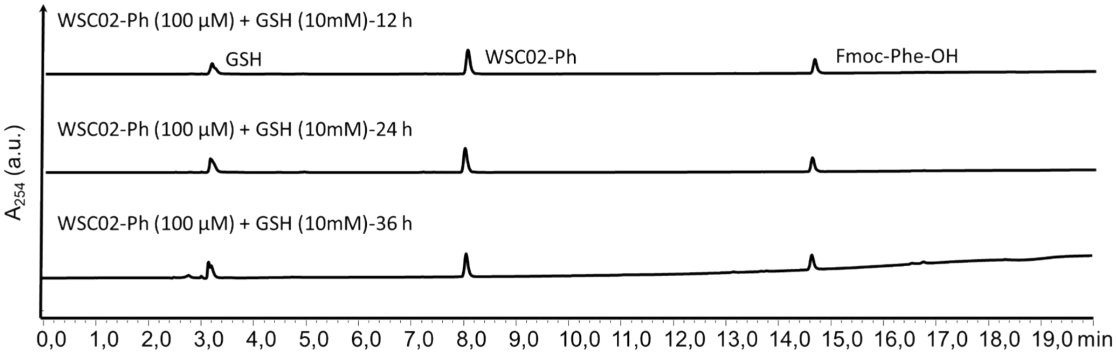

Fig. S23 Stability study of WSC02-Ph (100 µM) under 10 mM GSH for different time (12 h, 24 h, and 36 h).

5.3 Cysteine modification of other thiol-containing peptides

Besides WSC02 peptides, five other peptide substrates (RGDC, CEIE, PC-8, Tet and EK1 peptide) were also successfully modified following the similar procedures described above. Briefly, the peptide substrate (1 eq) was dissolved in ACN : PB (pH = 7) mixture with a concentration of 1 mg/mL. Compound **3** (1.2 eq, 20 mg/mL in DMF stock solution) was added and the mixture was incubated at room temperature for 4 hours. Next, 10 μL of mixture was took and diluted with 200 μL methanol. The modified peptide conjugates were purified by analytical HPLC with using the general methods described above and the isolated yield for each conjugate are 85% (RGDC), 84% (CEIE), 78% (PC-8), 83% (Tet peptide) and 77% (EK1 peptide). The LC-MS of the modified peptide conjugate was shown below.

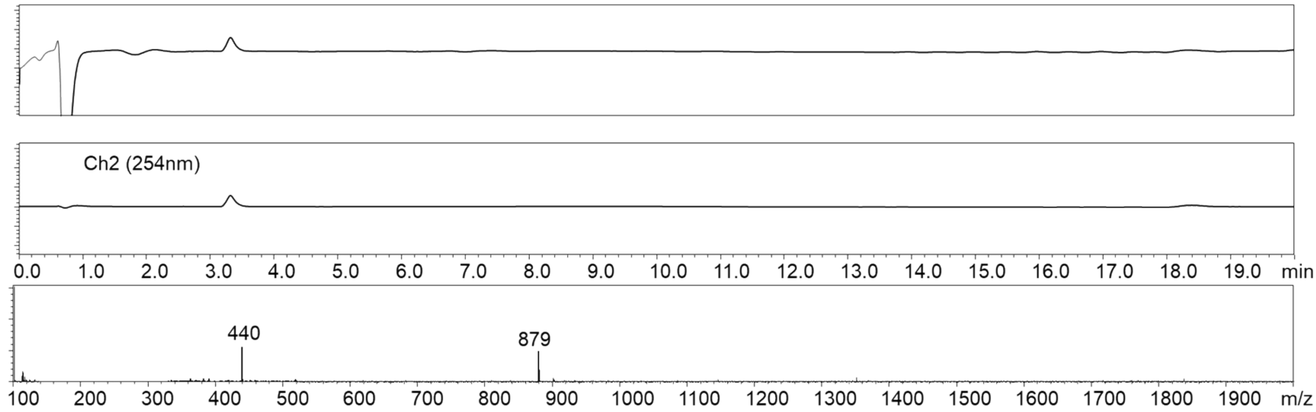

Fig. S24 LC-MS of modified peptide RGDC (calculated: 440 $[M+2H]^{2+}$, 879 $[M+H]^{+}$, found: 879 $[M+H]^{+}$, 440 $[M+2H]^{2+}$)

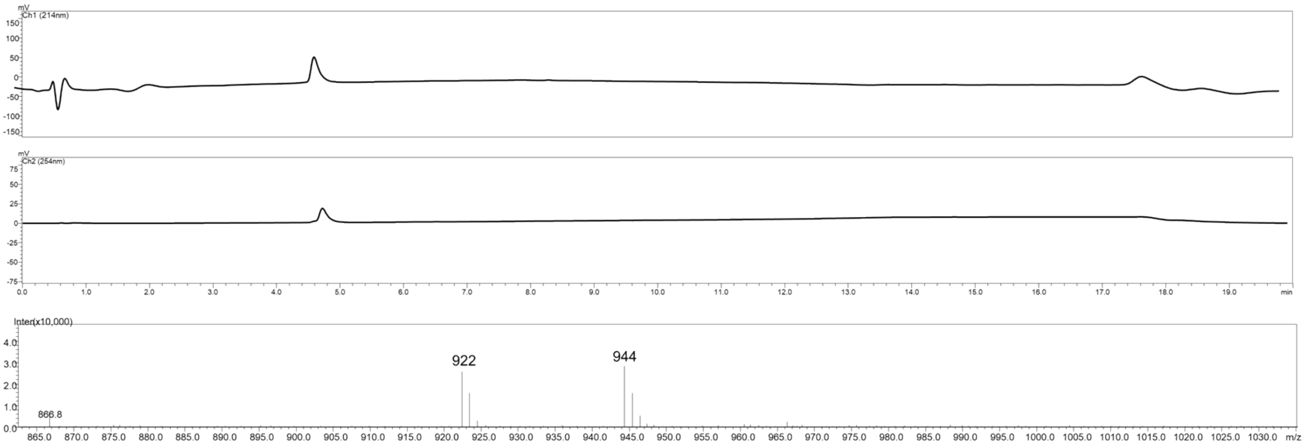

Fig. S25 LC-MS of modified peptide CEIE (calculated: 922 [M+H]+, 944 [M+2H]2+, found: 922 [M+H]+, 944 [M+2H]2+)

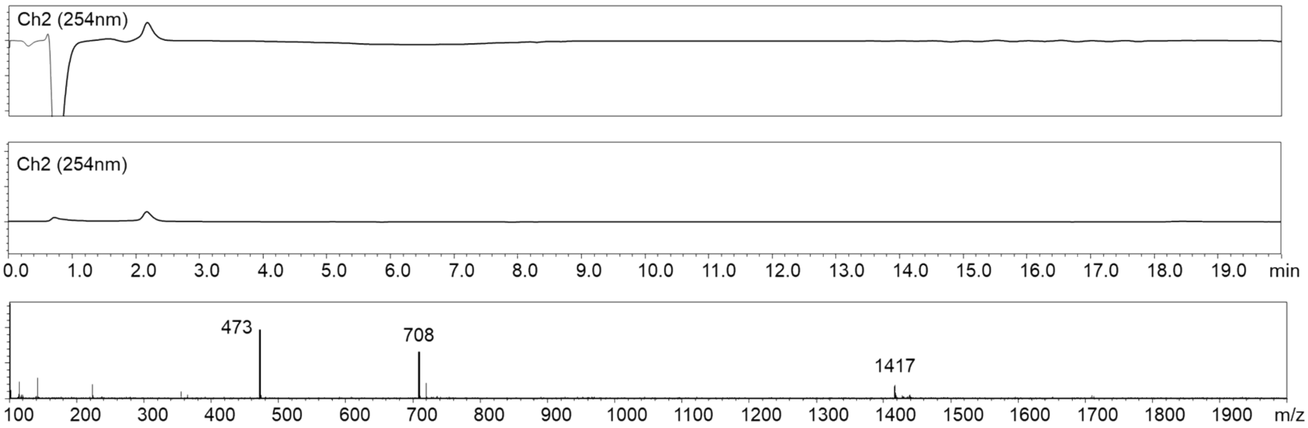

Fig. S26 LC-MS of modified peptide PC-8 (calculated: 473 [M+3H]2+, 708 [M+2H]2+, 1417 [M+H]+, found:473 [M+3H]2+, 708 [M+2H]2+, 1417 [M+H]+)

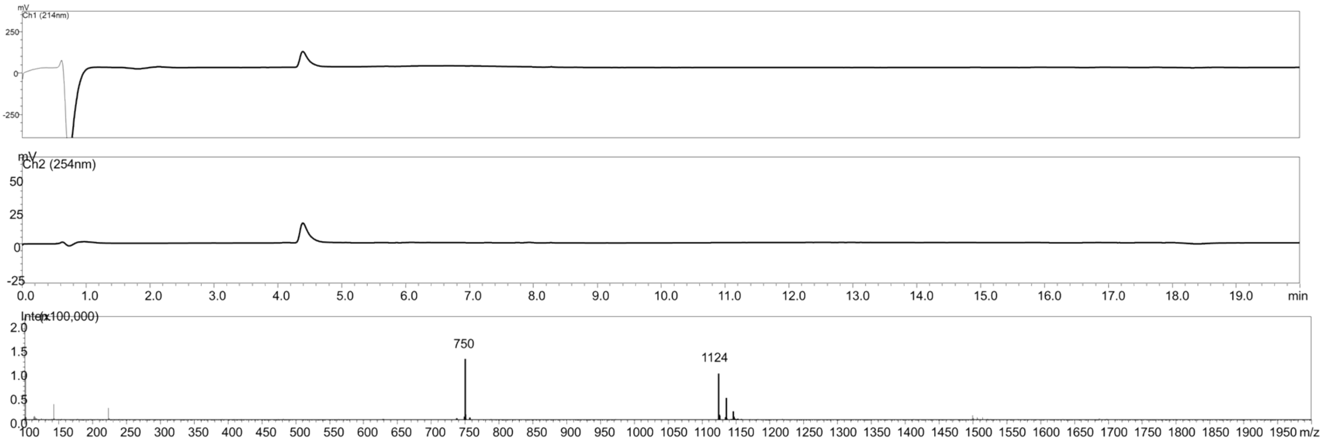

Fig. S27 LC-MS of modified Tet peptide (calculated: 750 [M+3H]3+, 1124 [M+2H]2+, found: 750 [M+3H]3+,

1124 [M+2H]$^{2+}$)

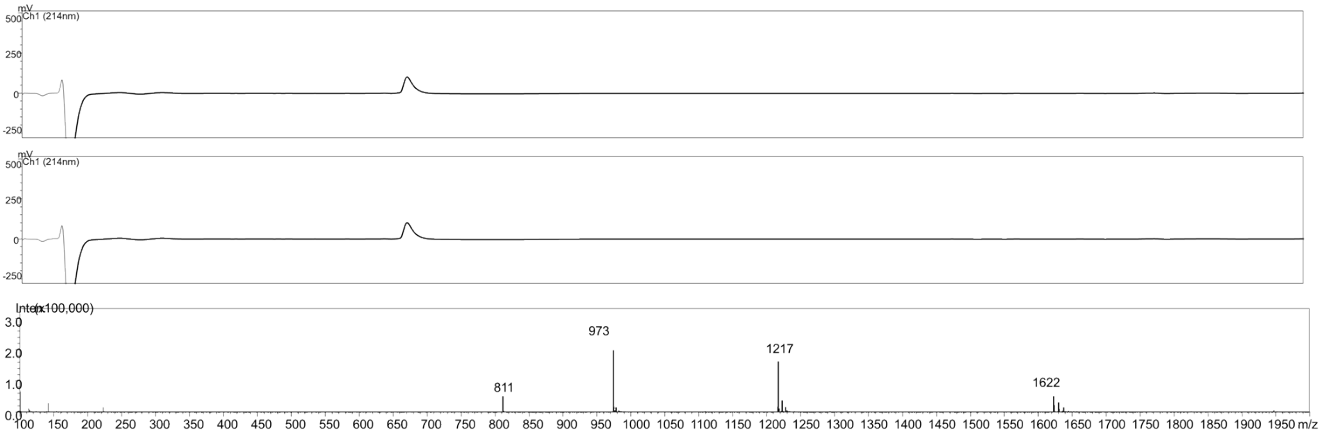

Fig. S28 LC-MS of modified EK1 peptide (calculated: 811[M+6H]$^{6+}$, 973 [M+5H]$^{5+}$, 1217 [M+4H]$^{4+}$, 1622 [M+3H]$^{3+}$, found: 811[M+6H]$^{6+}$, 973 [M+5H]$^{5+}$, 1217 [M+4H]$^{4+}$, 1622 [M+3H]$^{3+}$)

6. Ubiquitin modification with 2-chloromethyl acrylamide compounds

6.1 Cysteine modification of ubiquitin with 2-chloromethyl acrylamide compounds

(a). (b).

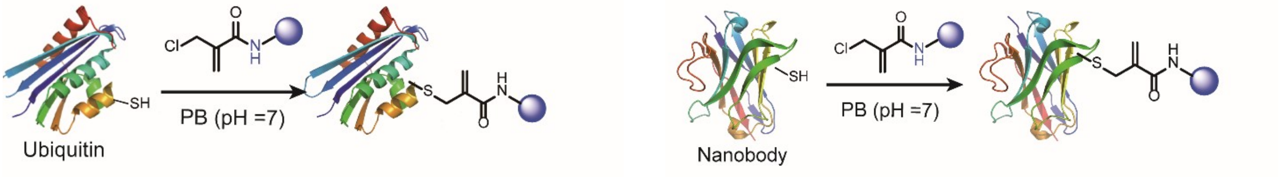

Scheme S7 Site-selective modification of (a) ubiquitin and (b) anti-GFP nanobody with 2-chloromethyl acrylamide compounds at cysteine site

Ubiquitin-K63C (Ub-K63C), in which lysine 63 has been mutated to a cysteine residue, was selected as model substrate to evaluate the modification efficiency on the protein level. Ub-K63C was purchased from the company UbiQ as lyophilized powder. The sequence of Ub-K63C: MQIFVKTLTGKTITLEVEPSDTIENVKAKI QDKEGIPPDQQRLIFAGKQLEDGRTLSDYNIQ**C**ESTLHLVLRLRGG. Its molecular weight is 8540 Da which was confirmed by MALDI-Tof-MS.

The general procedure to modify the ubiquitin substrate is as follows. First, ubiquitin was dissolved in

DMSO to prepare the stock solution with the concentration of 40 mg/mL. To an Eppendorf containing 8 µL PB (pH = 7) buffer, 8 µg Ub-K63C (1 eq) was added and the resulting mixture was vortexed for 20 seconds. Tris(2-carboxyethyl)phosphine (TCEP) (2eq, dissolved in PB buffer) was added to the ubiquitin solution and incubated for 30 minutes. Afterwards, compound **1** (10 eq, 20 mg/mL stock solution in DMF) or compound **3** (10 eq, 40 mg/mL stock solution in DMF) was added and the resulting mixture was incubated for another 4 hours at room temperature. Next, 5 µL aliquot was analyzed by MALDI-Tof-MS. The peak for Ub-K63C totally disappeared in MALDI-Tof-MS spectra while the product peaks (observed MS 8713 for Ub-Ph and 8970 for Ub-PEG4-Tz) were observed indicating the very excellent modification efficiency.

Similarly, if Ub-K63C reacted with 4-DPS first to mask the free cysteine residue on the protein surface, no further reaction was observed when adding compound **3** into it using the same conditions described above

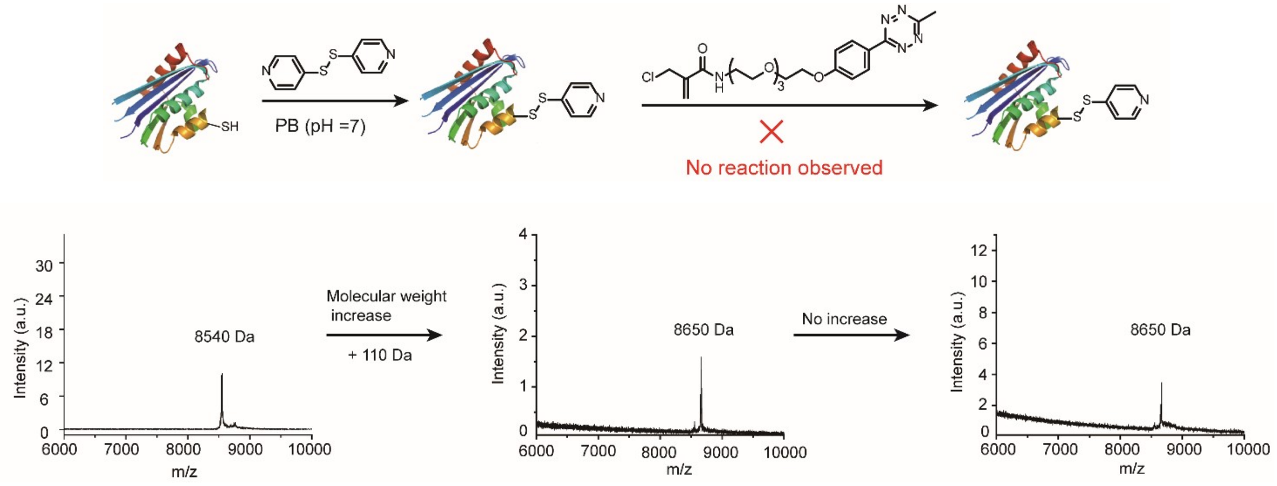

Fig. S29 Modification of ubiquitin with 4-DPS first to mask the accessible cysteine residue which followed the addition of compound **3.**

Nanobodies are small, antigen-binding, single-domain polypeptides derived from the variable heavy chain, which are considered as potent alternatives to conventional antibodies with enhanced stability and reduced size but similar antigen-binding characteristics.[9-10] The anti-GFP nanobody used in this study was purchased from NanoTag Biotechnologies, which contains a solvent accessible mutated cysteine residue.

The sequence of anti-GFP nanobody: MADVQLVESGGALVQPGGSLRLSCAASGFPVNRYSMRWYRQAP GKEREWAVGMSSAGDRSSYEDSVKGRFTISRDDARNTVYLQMNSLKPEDTAVYYCNVNVGFEYWGQGT QVTVSSADPNSSSVDKLACALEHHHHHH. The modification of anti-GFP nanobody at cysteine site with 2-chloromethyl acrylamide compounds was following the same procedures described for ubiquitin. After the reaction, the reaction mixture was characterized by MALDI-Tof-MS as sinapinic acid as matrix. The results are shown in Fig. 5b of the main text.

6.3 CD spectra of ubiquitin and Ub-PEG4-Tz

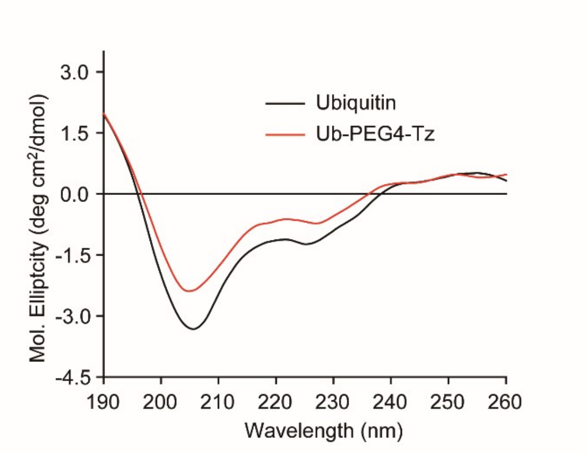

Fig. S30 CD spectra of ubiquitin and Ub-PEG4-Tz (0.1 mg/mL in PB (pH 7.4)).

7. Disulfide modification of somatostatin and Octreotide

7.1 Disulfide modification of somatostatin

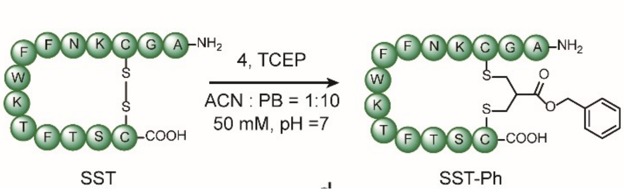

Scheme S8 Disulfide modification of SST with compound **4**

Somatostatin (SST, 1 mg, 1 eq, 0.61 μmol) was dissolved in 1 mL ACN / PB (50 mM, pH = 7) (v/v = 1:10). TCEP (0.35 mg, 2eq, 1.22 μmol) was dissolved in 10 μL ACN/PB (50 mM, pH = 7) (v/v = 1:10) and added to the SST solution. The mixture was incubated at room temperature for 30 minutes. Thereafter, compound **4**

(0.14 mg, 1.1 eq, 0.67 μmol, 40 mg/mL in DMSO stock solution) was also added in one-pot and the resulting mixture was gently shaken at room temperature overnight. 10 μL crude mixture was diluted with 200 μL methanol and injected to LC-MS directly to check the modification efficiency. The rest of the mixture was purified with analytical HPLC by using the general methods described above.

Next, 4-DPS was used to investigate if there are any free thiol groups in the modified SST-Ph. If the modification did not take place at the disulfide site, after the reduction of TCEP, the free thiol groups would still be able to react with 4-DPS, leading to a clearly detectable molecular weight increase. Consequently, purified SST-Ph (94 μg, 1 eq, 0.052 μmol) was dissolved in 94 μL citrate buffer and TCEP (59 μg, 4 eq, 0.20 μmol) was added. The mixed solution was incubated at room temperature for 60 minutes to ensure that TCEP reduced the disulfide bond if they are existing in the SST-Ph compound. Thereafter, 4-DPS (57 μg, 5 eq, 0.26 μmol) was added and the mixture was incubated for another 4 h. 10 μL of the obtained mixture was diluted with 200 μL methanol and injected to LC-MS to check if there is any further reaction between SST-Ph and 4-DPS. From the LC-MS data, no reaction was observed as the retention time of the SST-Ph was not changed and the molecular weight remained the same. As a control, native SST (94 μg, 1 eq, 0.057 μmol) was also incubated with TCEP (59 μg, 4 eq, 0.20 μmol), followed by the addition of 4-DPS (57 μg, 5 eq, 0.26 μmol). HPLC data clearly showed the occurrence of a new peak with a different retention time (Fig. S32).

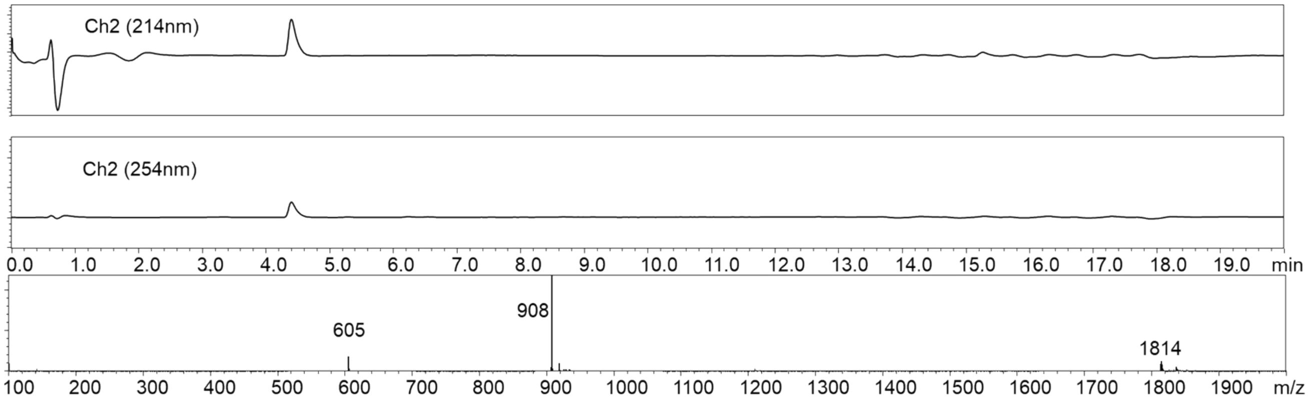

Fig S31 LC-MS of the purified SST-Ph (calculated: 604 $[M+3H]^{3+}$, 908 $[M+2H]^{2+}$, 1814 $[M+H]^{+}$, found: 605 $[M+3H]^{3+}$, 908 $[M+2H]^{2+}$, 1814 $[M+H]^{+}$)

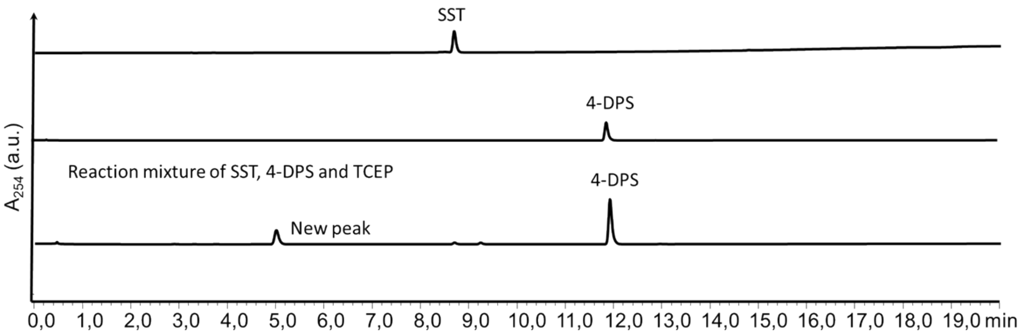

Fig. S32 HPLC analysis of the reaction between native somatostatin (SST) that contains a disulfide bond and 4-DPS**.** A new peak was formed after the reaction of SST with 4-DPS. However, the peak of SST-Ph, which was functionalized at the disulfide site, remained intact in the presence of 4-DPS indicating that the modification took place at the disulfide site.

The stability of the disulfide modified SST was also evaluated in pH 6, 7, and 8 as well as against GSH, which is depicted below.

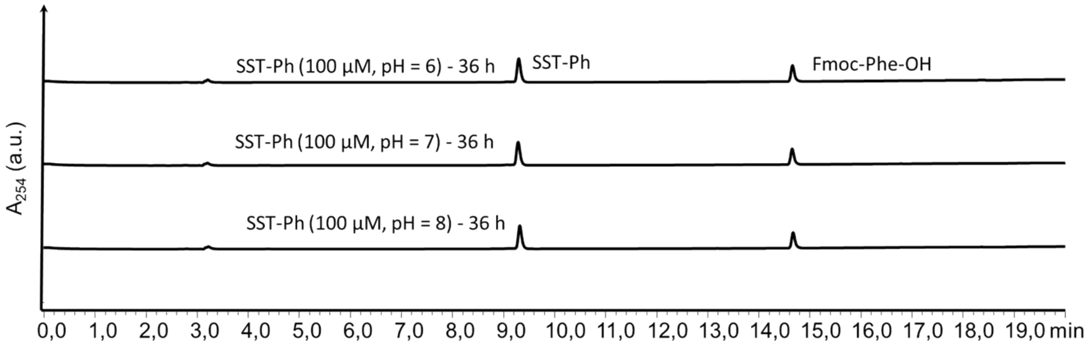

Fig. S33. Stability study of SST-Ph (100 µM) under three different pH for 36 h.

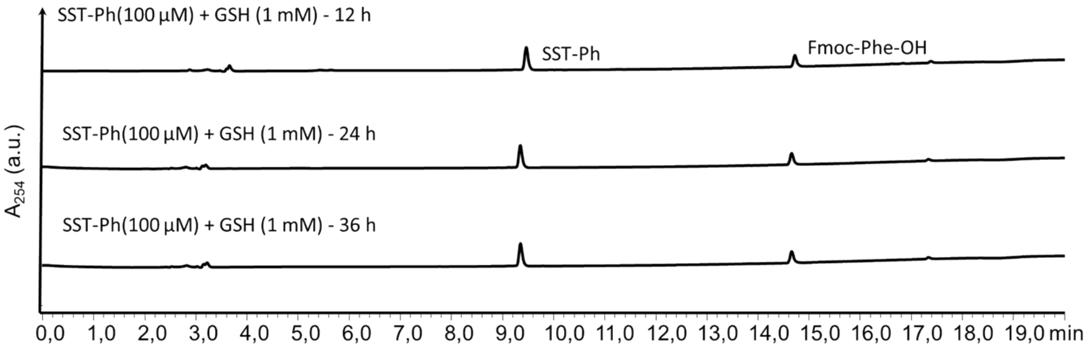

Fig. S34 Stability study of SST-Ph (100 µM) under 1 mM GSH for different time (12 h, 24 h, and 36 h).

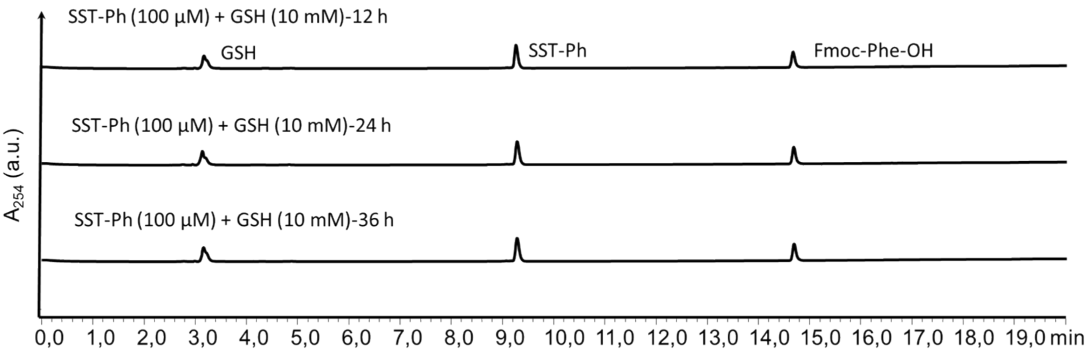

Fig. S35 Stability study of SST-Ph (100 μM) under 10 mM GSH for different time (12 h, 24 h, and 36 h)

CD spectra of native SST and SST-Ph

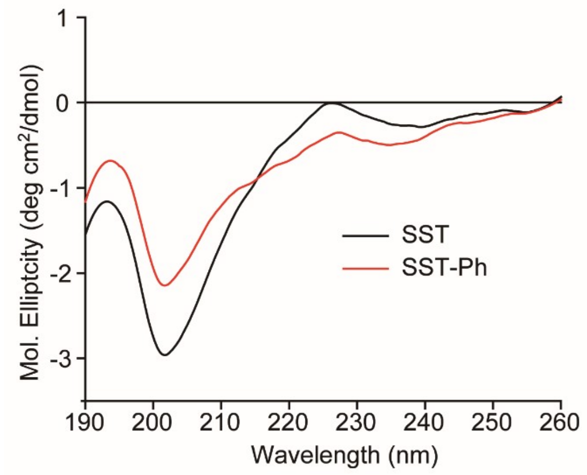

Fig. S36 CD spectra of native SST and modified one (SST-Ph)

7.2 Disulfide modification of somatostatin with compound **8** and IC-Tz

In order to compare the disulfide modification efficiency of the 2-chloromethyl acrylate compounds developed in this paper and the allyl sulfone reagents reported previously by our group, an allyl sulfone reagent (denoted as “IC-Tz”), which contained the same PEG chain length as compound **8**, was synthesized by following the protocol published before.[11] SST modification with compound **8** was following the procedure described above. For the modification with IC-Tz, SST (1 mg, 1 eq, 0.61 μmol) was dissolved in ACN: PB (pH =7.8) (v/v = 2:3). TCEP (0.35 mg, 2 eq,1.2 μmol) was added. The mixture was incubated at room temperature for 30 minutes. Afterwards, IC-Tz (0.66 mg, 2 eq, 1.2 μmol) was also added and the resulting mixture was gently shaken at room temperature overnight. 10 μL crude mixture was diluted with 200 μL methanol and

injected to HPLC directly to check the modification efficiency. The result is shown in Fig 6e of the main text.

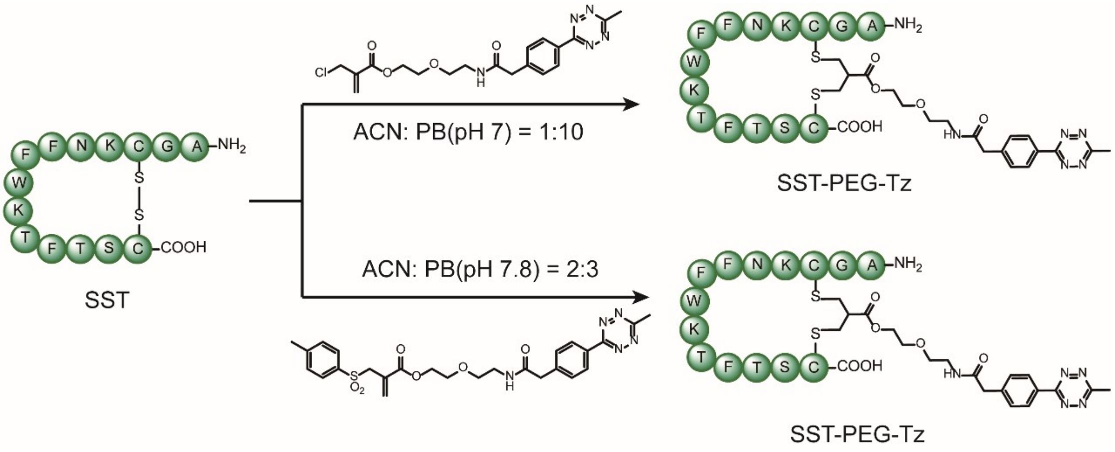

Scheme S9 Somatostatin modification with two different disulfide rebridging reagents.

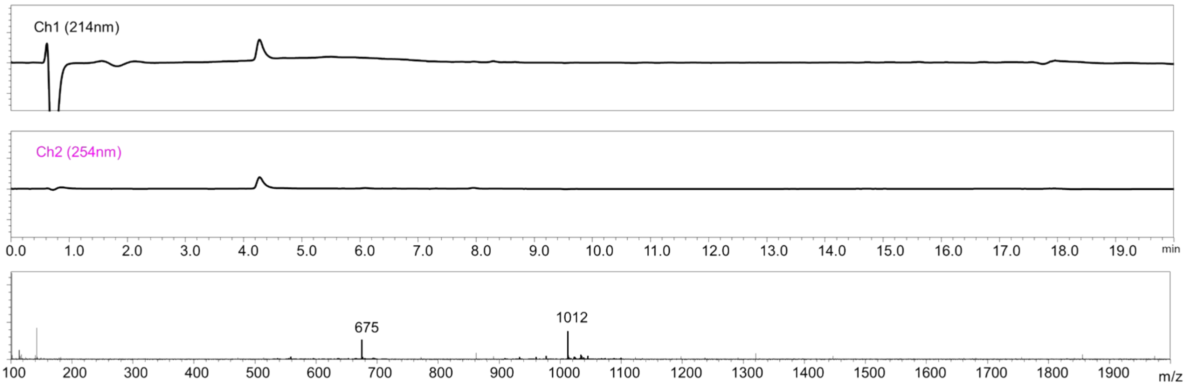

Fig. S37 LC-MS of SST-PEG-Tz (calculated: 1011 $[M+2H]^{2+}$, 674 $[M+3H]^{3+}$, found: 1012 $[M+2H]^{2+}$, 675 $[M+3H]^{3+}$

7.3 Trypsin digestion of SST-PEG-Tz

SST-PEG-Tz (0.83 µg/µL; 0.45 mM in water) (50 µL, 0.023 µmol) was added to 62.6 µL ammonium bicarbonate (200 mM, 16 mg/mL), followed by the addition of trypsin (0.1 µg/µL) (10.4 µL, 0.56 nmol). The resultant mixture was incubated for 2 h at 37 °C. Samples for LC-MS analysis were diluted 4 times in ammonium acetate solution (20 mM, pH 7.0).

Liquid chromatography–mass spectrometry (LC-MS) measurements were conducted using a Dionex Ultimate 3000 UHPLC+ system equipped with a Multiple-Wavelength detector, an imChem Surf C18 TriF 100 Å 3 µm 100 x 2.1mm column connected to Thermo Scientific Q Exactive hybrid quadrupole-Orbitrap mass spectrometer (Thermo Scientific^{TM} Q Exactive^{TM} Plus). Tryptic peptides were separated with a 0.2 mL/min and mixture of water with 0.1% formic acid (buffer A) acetonitrile with 0.1% formic acid (buffer B), according to the following table:

Time (min)	A (%)	B (%)	curve
0-5	97 → 97	3 → 3	5
5-75	97 → 5	5 → 95	7
75-85	5 → 5	95 → 95	5
85-88	5 → 97	95 → 3	5
88-90	97 → 97	3 → 3	3

The exactive mass spectrometer was operated in the positive ion mode with alternating MS scans of the precursor ions and AIF (all ion fragmentation) scans in which the peptides were fragmented by SID (Surface-Induced Dissociation) of 50 eV. Both scan types were performed with 70,000 resolution (at *m/z* 200) with each scan taking 0.05 s, and the maximum fill time was set to 1 s as well. The *m/z* range for the MS scans was selected between 300–1600, and the *m/z* range for AIF scans was chosen between 150–1600. The target value for the MS scans was 10^6 ions, and the target value for the AIF scans was 3 x 10^6 ions.

In order to confirm the structure of the double modification, trypsin digestion was performed. Taking in consideration that trypsin will cut the peptide at Lys (K) and Arg (R), the extracted ion chromatogram (EIC) within a 6 ppm range charged by 2-3 H^+ was searched for the fragments (Figures S36-44). A fragment was observed with single modification at the C-terminal Cys but was determined to occur via a retro-Michael reaction during the digestion process (data not shown).

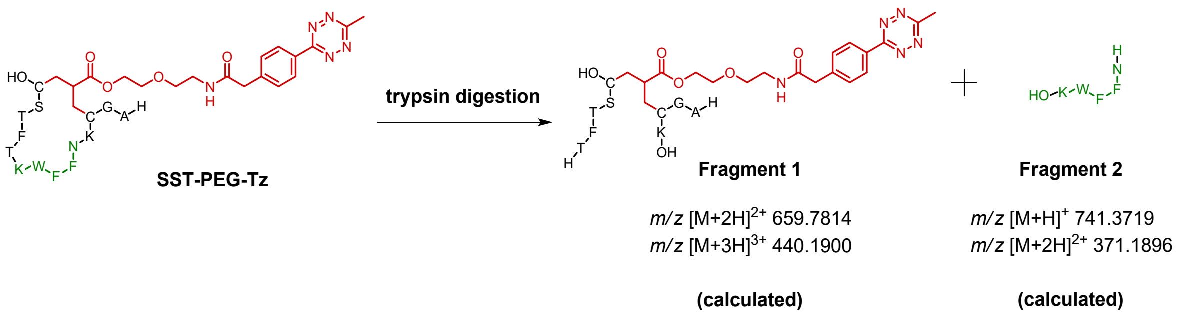

Scheme S10 Fragment 1 and Fragment 2 formed from trypsin digestion of SST-PEG-Tz

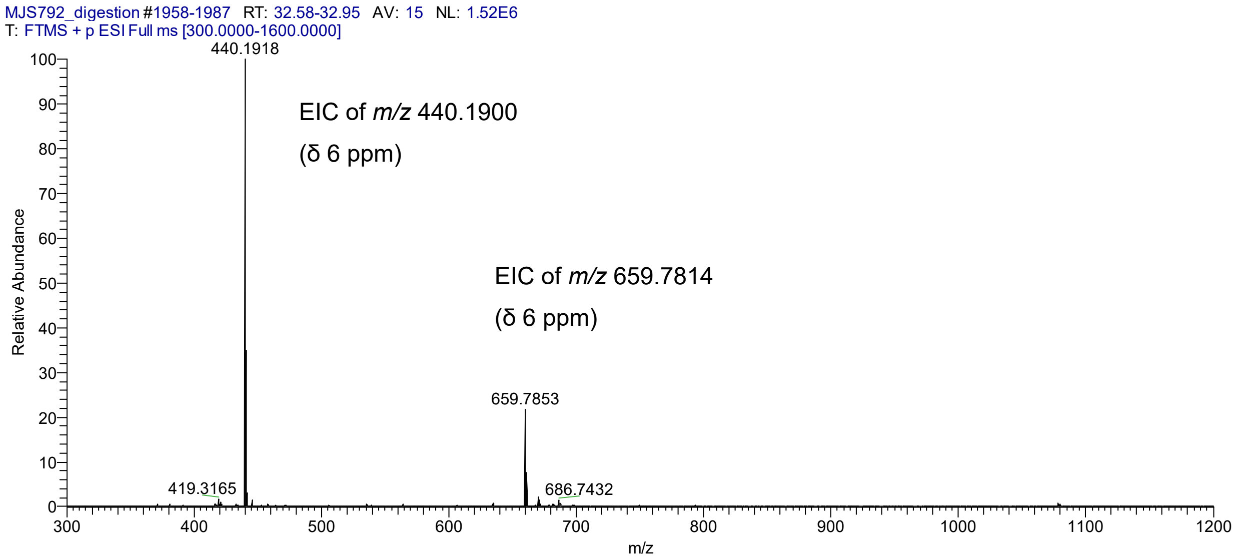

Fig. 38 ESI-MS of fragment 1

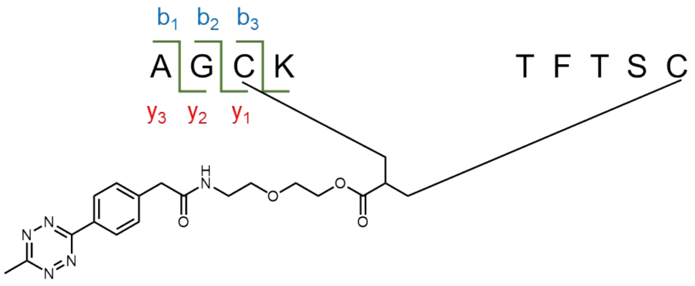

Lost fragment	#b	b^+	b^{2+}	Seq	y^+	y^{2+}	#y	Lost fragment
A	1	72.0444	36.02222	A	1318.5555	659.78135	3	-
AG	2	129.0659	64.53295	G	1247.51826	623.75913	2	A
-	3*	-	-	C	1190.49680	595.2484	1*	AG

Table S2 Expected b and y ions from AIF (ions that are found are highlighted in red)

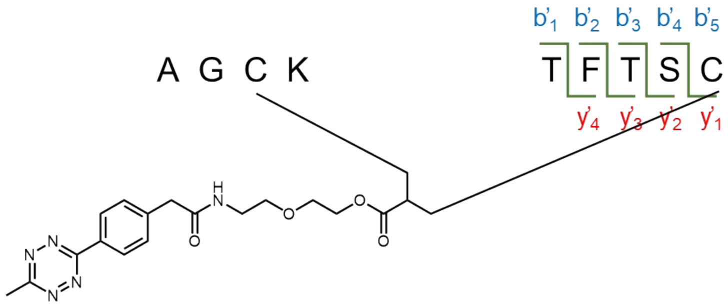

Lost fragment	#b'	b^+	b^{2+}	Seq	y^+	y^{2+}	#y'	Lost fragment
H_2O	5*	1301.5521	650.7761	T	18.01056	9.00528	1*	H_2O
T	4	1217.5077	608.7539	F	102.055	51.0275	2	T
TF	3	1070.4393	535.2196	T	249.12341	124.561705	3	TF
TFT	2	969.3916	484.6958	S	350.17109	175.085545	4	TFT

Table S3 - Expected b and y ions from AIF (ions that are found are highlighted blue and red)

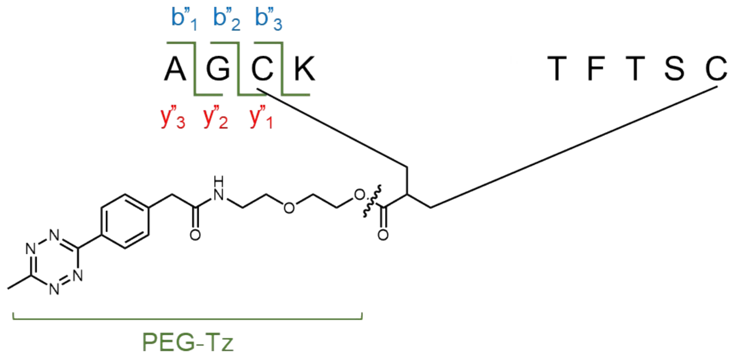

Lost fragment	#b''	b^+	b^{2+}	Seq	y^+	y^{2+}	#y''	Lost fragment
A	1	72.0444	36.02222	A	930.3696	465.1848	3	A
AG	2	129.0659	64.53295	G	873.3481	436.6741	2	AG

Table S4. Expected b and y ions from AIF (ions that are found are highlighted in red)

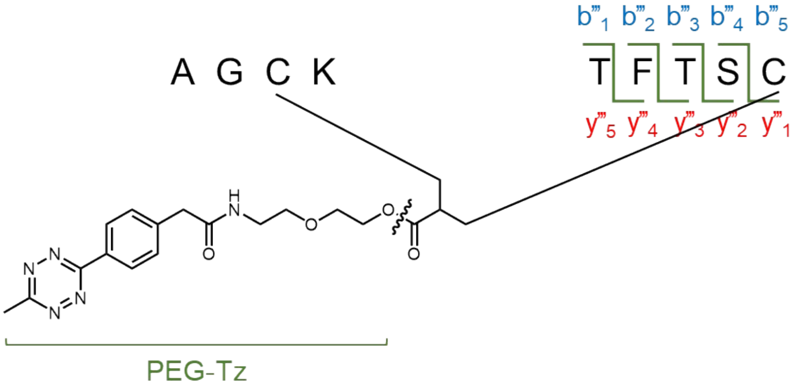

Lost fragment	#b'''	b^+	b^{2+}	Seq	y^+	y^{2+}	#y'''	Lost fragment
H_2O	5*	18.01056	9.00528	T	984.4034	492.2017	1*	H_2O
T	4	102.0550	51.0275	F	900.3590	450.1795	2	T
TF	3	249.1234	124.5617	T	753.2906	376.6453	3	TF
TFT	2	350.1711	175.0855	S	652.2429	326.1215	4	TFT

Table S5. Expected b and y ions from AIF (ions that are found are highlighted in red and blue)

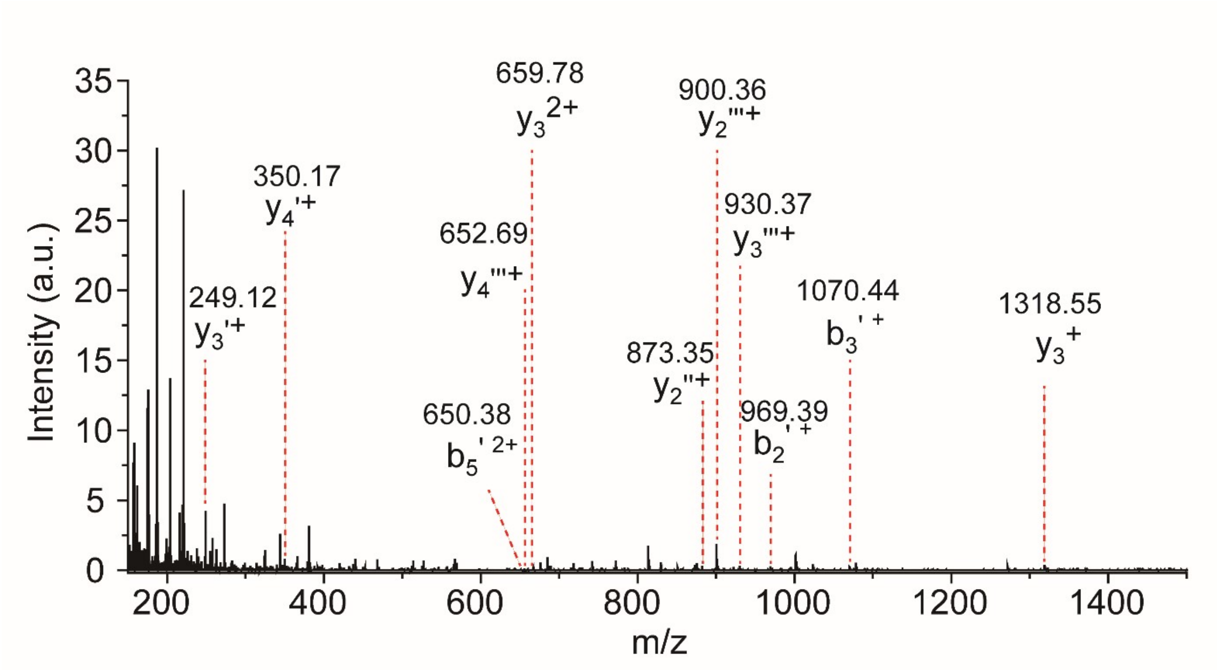

Fig. 39 Full spectrum of fragment 1 using SID 50 eV

Spectra zoom-in:

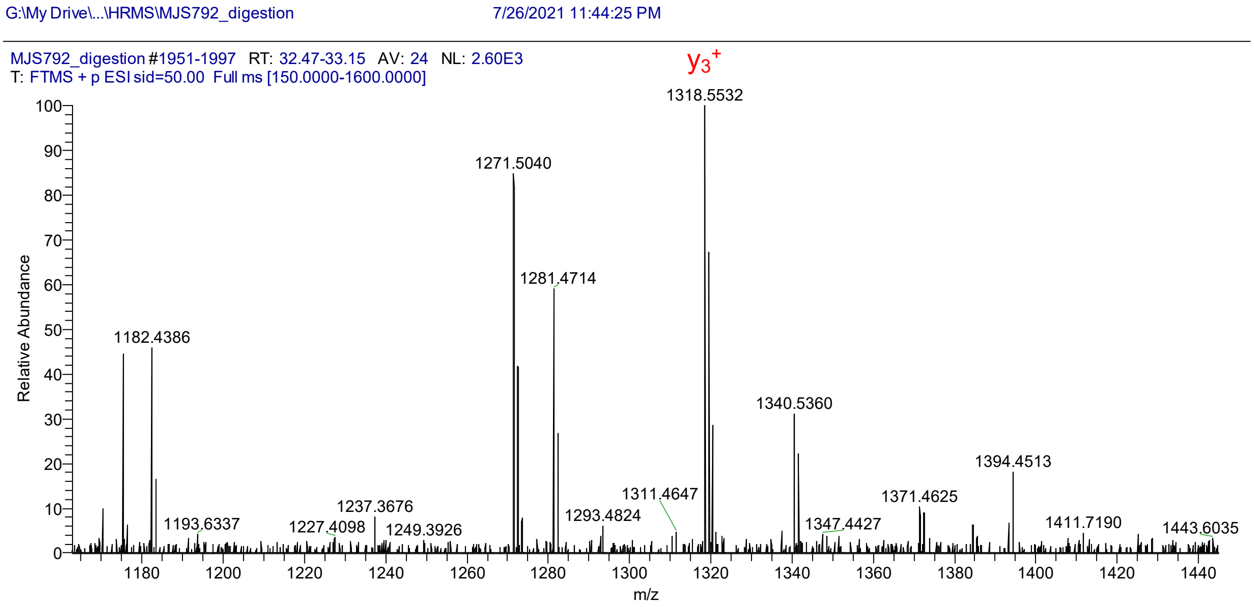

Fig S40. AIF spectrum of peak at 32.8 min using SID 50 eV between *m/z* 1150-1450 range.

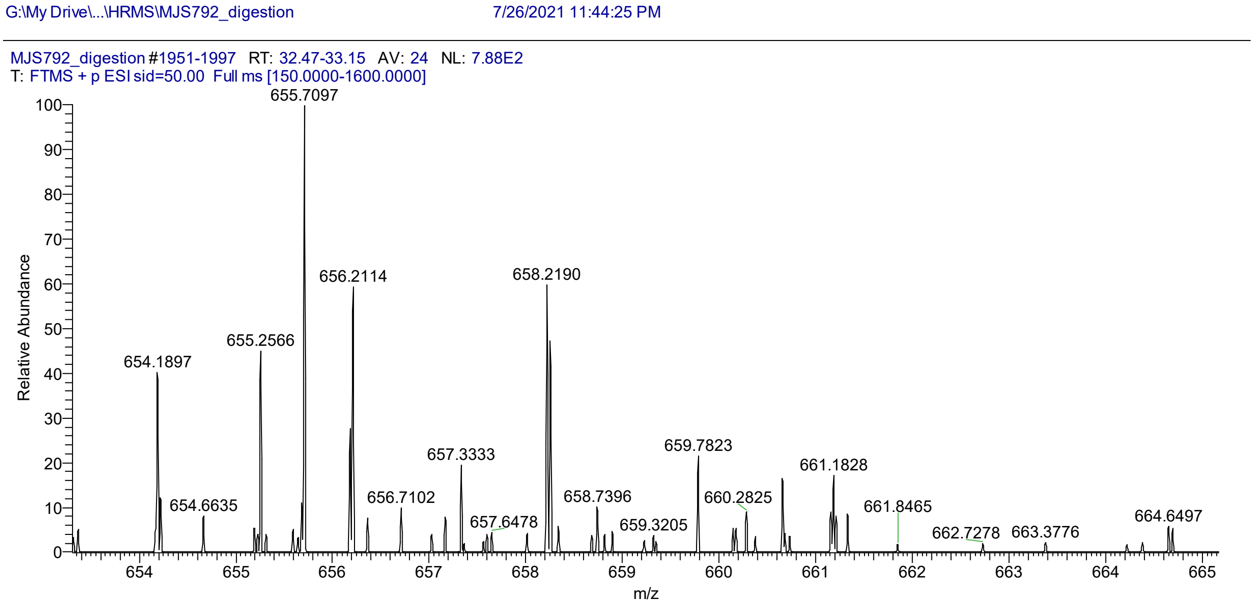

Fig S41. AIF spectrum of peak at 32.8 min using SID 50 eV between *m/z* 653-665 range.

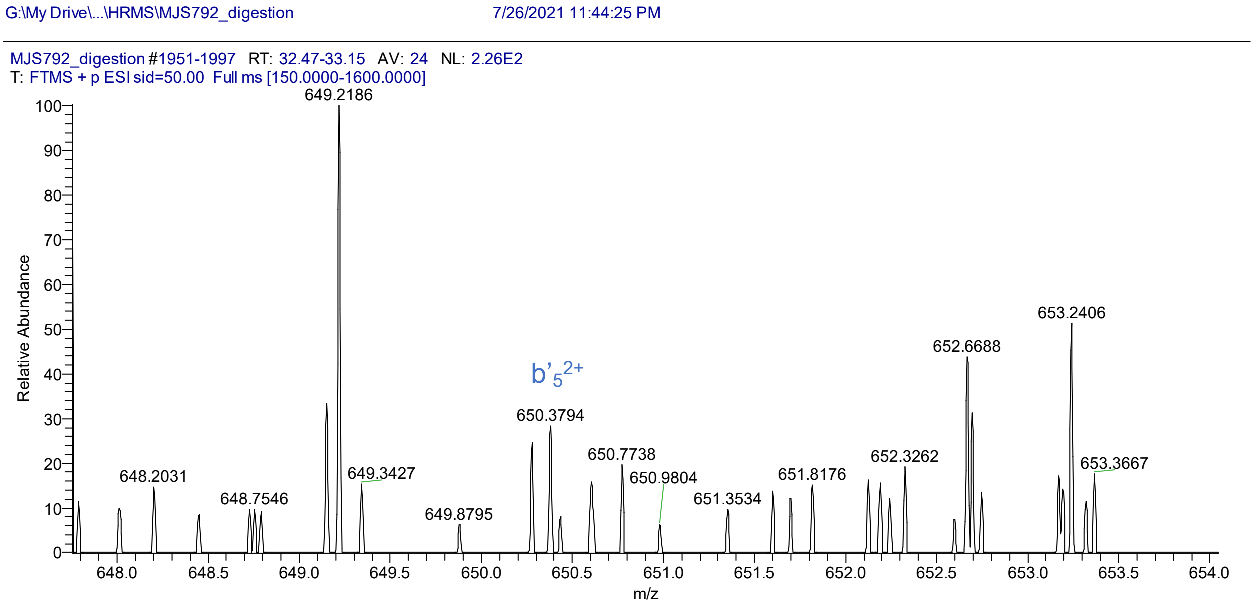

Fig S42. AIF spectrum of peak at 32.8 min using SID 50 eV between *m/z* 648-654 range.

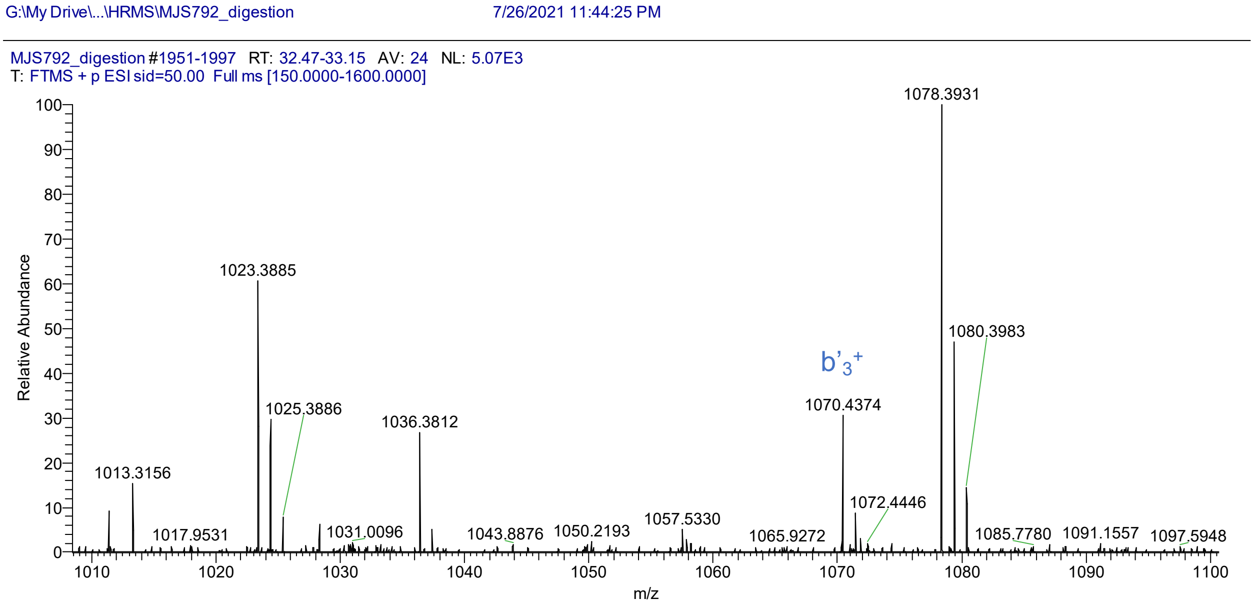

Fig S43. AIF spectrum of peak at 32.8 min using SID 50 eV between *m/z* 1010-1100 range.

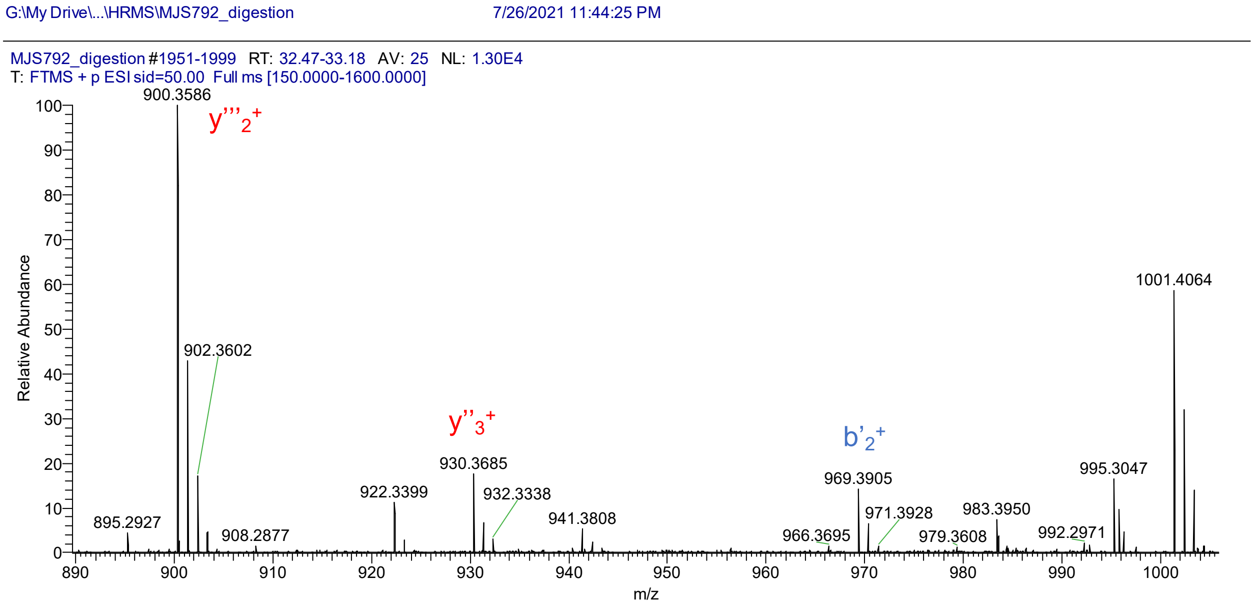

Fig S44. AIF spectrum of peak at 32.8 min using SID 50 eV between *m/z* 890-1005 range.

7.4 Disulfide modification of octreotide

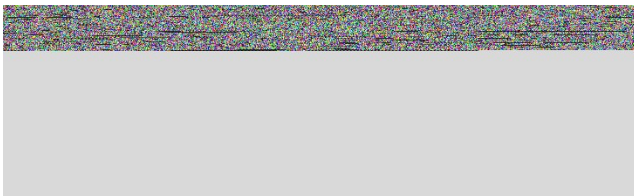

Scheme S11 Disulfide modification of octreotide with compound **6**

The modification of octreotide followed the similar procedure as the modification with SST. Briefly, octreotide (200 μg, 1 eq, 0.20 μmol) was dissolved in ACN/PB (50 mM, pH = 7) (v/v = 1:10) with the concentration of 1 mg/mL. TCEP (112 μg, 2 eq, 0.40 μmol) was also added and the resulting mixture was incubated at room temperature for 30 minutes to fully break the disulfide bond. Afterwards, compound **6** (89 μg, 1.2 eq, 0.24 μmol) was added to the aforementioned mixture above and incubated for overnight. After that, the crude mixture was purified with analytical HPLC to get a while solid. MALDI-Tof-MS data demonstrated the successful modification

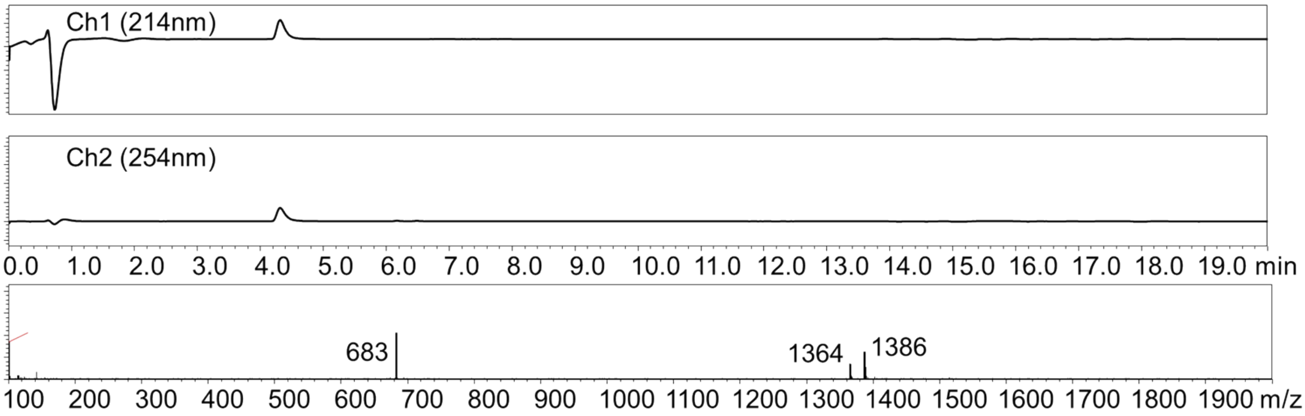

Fig. S45 LC-MS of modified octreotide with coumarin group (Oc-Coumarin) (calculated: 1364 $[M+H]^+$, found: 1364 $[M+H]^+$, 1386 $[M+Na]^+$, 683 $[M+2H]^+$)

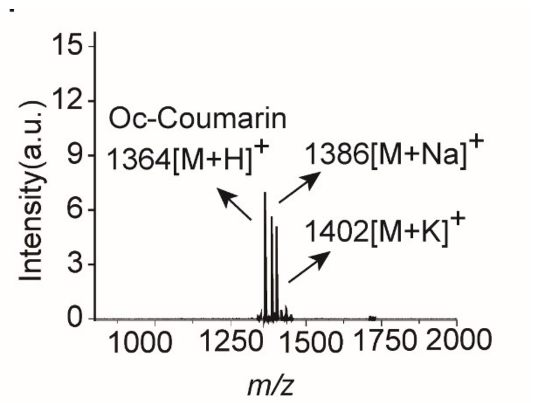

Fig. S46 MALDI-Tof-MS of Oc-Coumarin (calculated: 1364 $[M+H]^+$, 1386 $[M+Na]^+$, 1402 $[M+K]^+$, found: 1364

$[M+H]^+$, 1386 $[M+Na]^+$, 1402 $[M+K]^+$)

CD spectra of native octreotide and Oc-Cou

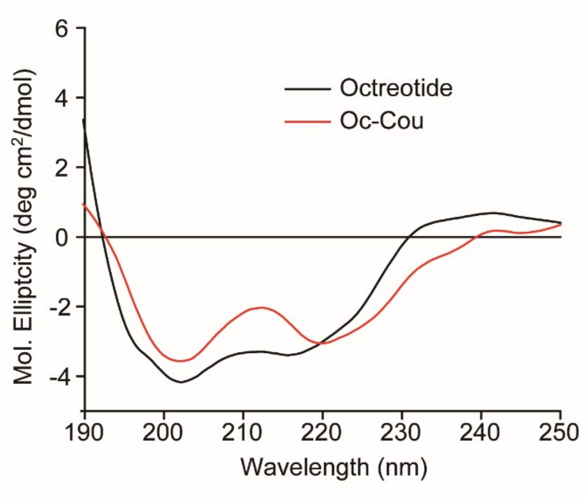

Fig. S47 CD spectra of native octreotide and Oc-Cou (0.1 mg/mL in PB (pH 7.4)).

8. Lysozyme modification

8.1 Disulfide modification of lysozyme

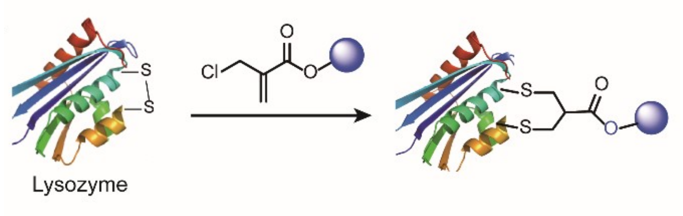

Scheme S12 Disulfide modification of lysozyme with 2-chloromethyl acrylate compounds

Lysozyme (500 μg, 1 eq, 0.035 μmol) was dissolved in 500 μL PB buffer (50 mM, pH = 7). TCEP (12 μg, 1.2 eq, 0.042 μmol) was dissolved in 10 μL PB buffer (50 mM, pH = 7) and added to the lysozyme solution. Subsequently, the respective 2-chloromethyl acrylate compounds, for example compound **8** stock solution (23 μg, 1.1 eq, 0.042 μmol) was added to the aforementioned lysozyme mixture in one-pot. The mixed solution was incubated for overnight at room temperature and purified with FPLC (ÄKTA from GE) by using Hi Trap phenyl HP column (1 mL, from GE) based on the different hydrophobicity of native and modified lysozyme. The collected fractions were concentrated with ultrafiltration tube and the solvent was removed with freeze dryer. The obtained modified lysozyme was characterized by MALDI-Tof-MS showing the correct MS.

8.2 Trypsin digestion of Ly-PEG-Tz

Lysozyme-PEG-Tz protein solution (9.6 μL, 100 μM) in ammonium acetate (20 mM, pH 7.0) was added to a solution of ammonium bicarbonate (14.4 μL, 16 mg/mL), followed by activated trypsin (7 μL, 0.1 μg/μL in ammonium bicarbonate (16 mg/mL) solution. The resultant mixture was incubated at 37 °C for 20 h. Thereafter, the reaction was quenched with formic acid (5 μL), vortexed briefly and then centrifuged.

LC-MS method

Liquid chromatography–mass spectrometry (LC-MS) runs were realized using a Dionex Ultimate 3000 UHPLC+ system equipped with a Multiple-Wavelength detector, an imChem Surf C18 TriF 100 Å, 3 μm 100x2,1 mm column connected to Thermo Scientific Q Exactive hybrid quadrupole-Orbitrap mass spectrometer (Thermo Scientific^{TM} Q Exactive^{TM} Plus). Tryptic peptides were separated with a 0.2 mL/min and mixture of water with 0.1% formic acid (buffer A) acetonitrile with 0.1% formic acid (buffer B), according to the following table:

Time (min)	A (%)	B (%)	curve
0-5	97 → 97	3 → 3	5
5-75	97 → 5	5 → 95	7
75-85	5 → 5	95 → 95	5
85-88	5 → 97	95 → 3	5
88-90	97 → 97	3 → 3	3

Table S6 HPLC method used for the analysis of Trypsin digestion of Ly-PEG-Tz

The exactive mass spectrometer was operated in the positive ion mode with alternating MS scans of the precursor ions and AIF (all ion fragmentation) scans in which the peptides were fragmented by SID (Surface-Induced Dissociation) with 50 eV. Both scan types were performed with 70,000 resolution (at *m/z* 200) with each scan taking 0.05 s, and the maximal fill time was set to 1 s as well. The *m/z* range for MS scans was 300–1600, and the *m/z* range for AIF scans was 150–1600. The target value for the MS scans was 10^6 ions, and the target value for the AIF scans was 3 x 10^6 ions.

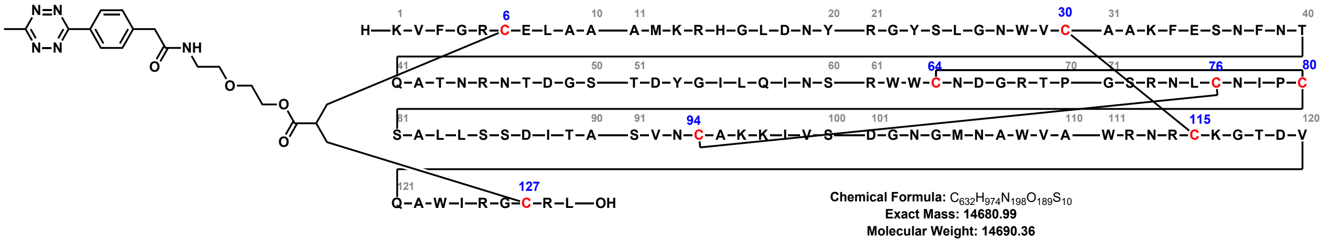

Fig. S48 Sequence profile and expected modification site of Ly-PEG-Tz.

Possible combinations for PEG-Tz crosslink in Lysozyme protein

To validate the site-selectivity of PEG-Tz crosslink for C6 and C127 of Lysozyme C (P00698), all possible S-S rebridging positions were considered. Skyline software[12] was used to generate the list of Lysozyme digestion fragments from *in silico* Trypsin digestion.[12] The simulation of protein digestion also considered zero missed cleavages, no Methionine oxidation and all Cys in reduced form. All generated fragments were combined and added PEG-Tz crosslink as a structural modification variable on Cys residues. For the analysis, the transition list was used to check Extracted Ion chromatogram (EIC) within a 6 ppm range for all possible fragments that included precursor charges of 1 to 6, within 300-1600 *m/z* range.

Peptide A	Peptide B	Precursor *m/z*					
		1+	2+	3+	4+	5+	6+
**C⁶ELAAAMK**	**GYSLGNWVC³⁰AAK**	1294.0733	1244.0845	829.7254	622.5459	498.2382	415.3664
**C⁶ELAAAMK**	**WWC⁶⁴NDGR**	-	1077.9690	718.9817	539.4881	431.7920	359.9945
**C⁶ELAAAMK**	**NLC⁷⁶NIPC⁸⁰SALLSSDITASVNC⁹⁴AK**	-	-	1185.8973	889.6748	711.9413	593.4523
**C⁶ELAAAMK**	**C¹¹⁵K**	1468.6745	734.8409	490.2297	367.9241	-	-
**C⁶ELAAAMK**	**GC¹²⁷R**	**1553.7022**	**777.3547**	**518.5722**	**389.1810**	311.5463	-
**GYSLGNWVC³⁰AAK**	**WWC⁶⁴NDGR**	-	1294.0733	863.0513	647.5403	518.2337	432.0293
**GYSLGNWVC³⁰AAK**	**NLC⁷⁶NIPC⁸⁰SALLSSDITASVNC⁹⁴AK**	-	-	1329.966854	997.72696	798.383023	665.4871
**GYSLGNWVC³⁰AAK**	**C¹¹⁵K**	-	950.9453	634.2993	475.9763	380.9825	317.6533
**GYSLGNWVC³⁰AAK**	**GC¹²⁷R**	-	993.4591	662.6418	497.2332	397.9880	331.8245
**WWC⁶⁴NDGR**	**NLC⁷⁶NIPC⁸⁰SALLSSDITASVNC⁹⁴AK**	-	-	1219.2231	914.6692	731.9368	610.1152
**WWC⁶⁴NDGR**	**C¹¹⁵K**	1568.6522	784.8297	523.5556	392.9185	314.5363	-
**WWC⁶⁴NDGR**	**GC¹²⁷R**	-	827.3435	551.8981	414.1754	331.5418	-
**NLC⁷⁶NIPC⁸⁰SALLSSDITASVNC⁹⁴AK**	**C¹¹⁵K**	-	1485.2030	990.4711	743.1052	594.6856	495.7392
**NLC⁷⁶NIPC⁸⁰SALLSSDITASVNC⁹⁴AK**	**GC¹²⁷R**	-	1527.7168	1018.8136	764.3621	611.6911	509.9105
**C¹¹⁵K**	**GC¹²⁷R**	967.4237	484.2155	323.1461	-	-	-

*The ions found in the Trypsin digestion LC-MS are highlighted in green.

Table S7 Possible fragment combinations of Lysozyme protein with PEG-Tz crosslink modification from Trypsin digestion

Site-selective crosslink for C6 and C127

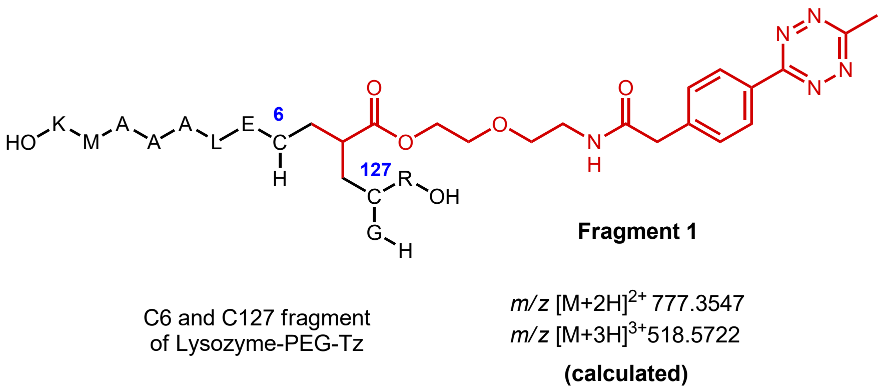

Fig. S49 Chemical structure of the C6 and C127 fragment.

Peptide Modified Sequence	Charge	Calculated *m/z*	Observed *m/z*	δ ppm
**C⁶ELAAAMK** - PEG-Tz - **GC¹²⁷R**<br>Fragment 1<br>**[M+383.1594+H_2O]**	1+	1553.7022	1553.7021	-0.06
	2+	777.3547	777.3577	3.86
	3+	518.5722	518.5723	0.19
	4+	389.1810	389.1821	2.83
	5+	311.5463	NF	-

*NF: not found

Table S8 Calculated and observed *m/z* values for Fragment 1 (highlighted in green)

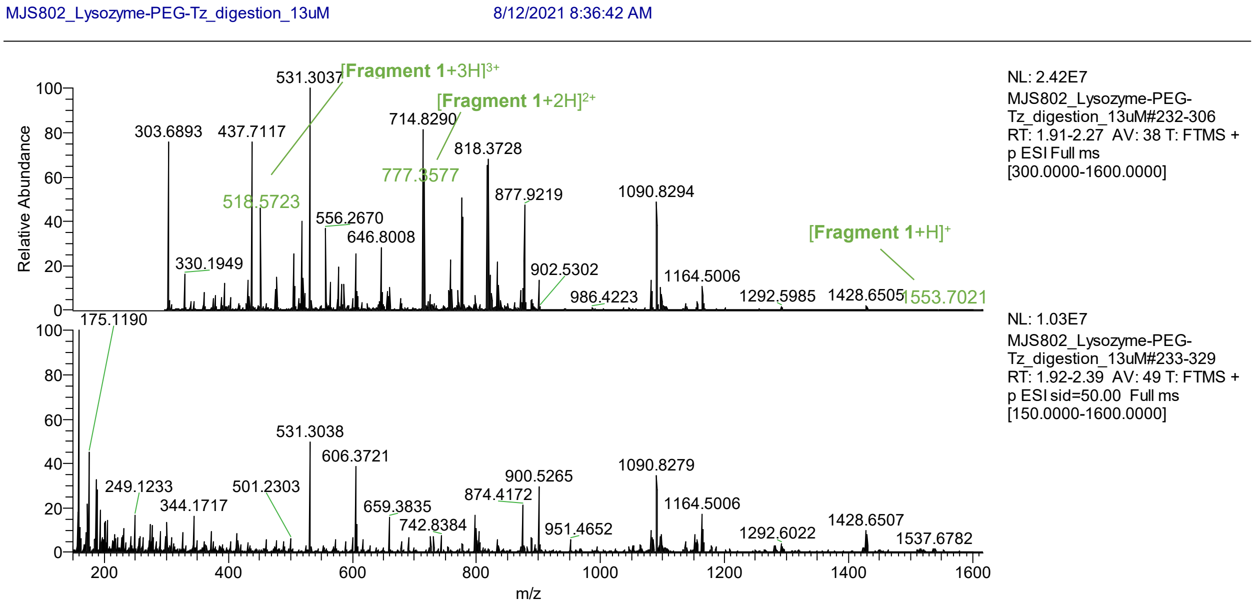

Fig. S50 Full spectrum of peak at 2.0 min, ions found are highlighted in green (top) and full spectrum from AIF spectrum of peak at 2.0 min using SID 50 eV (bottom).

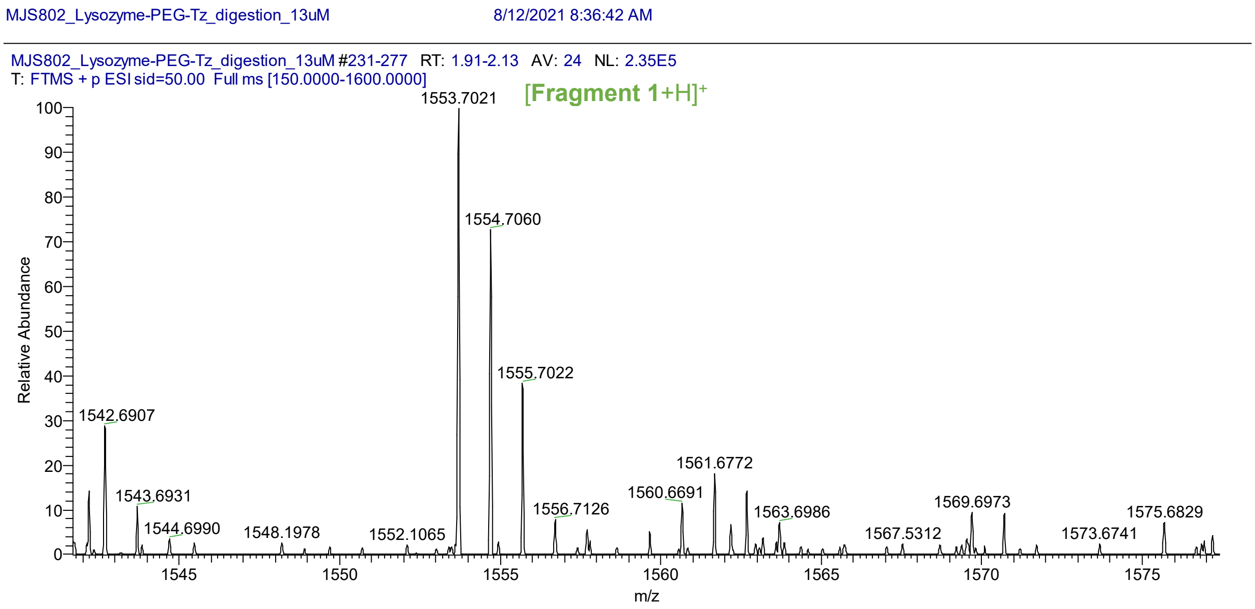

Fig. S51 Spectrum of peak at 2.0 min between 1540-1575 *m/z* range.

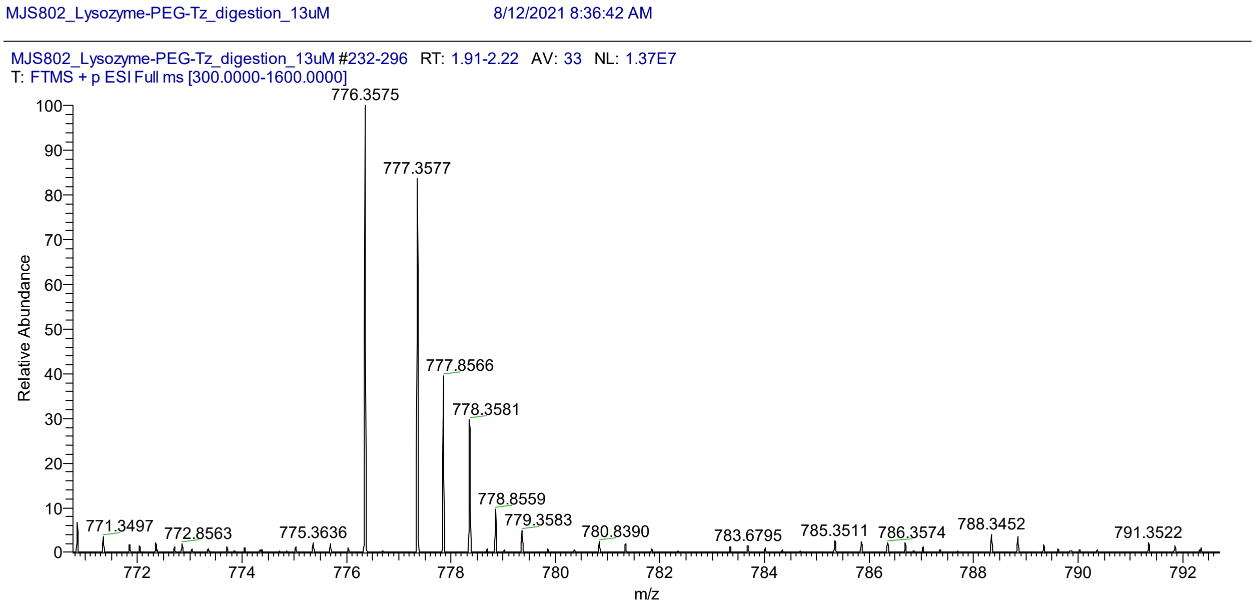

Fig. S52 Spectrum of peak at 2.0 min between 770-792 *m/z* range.

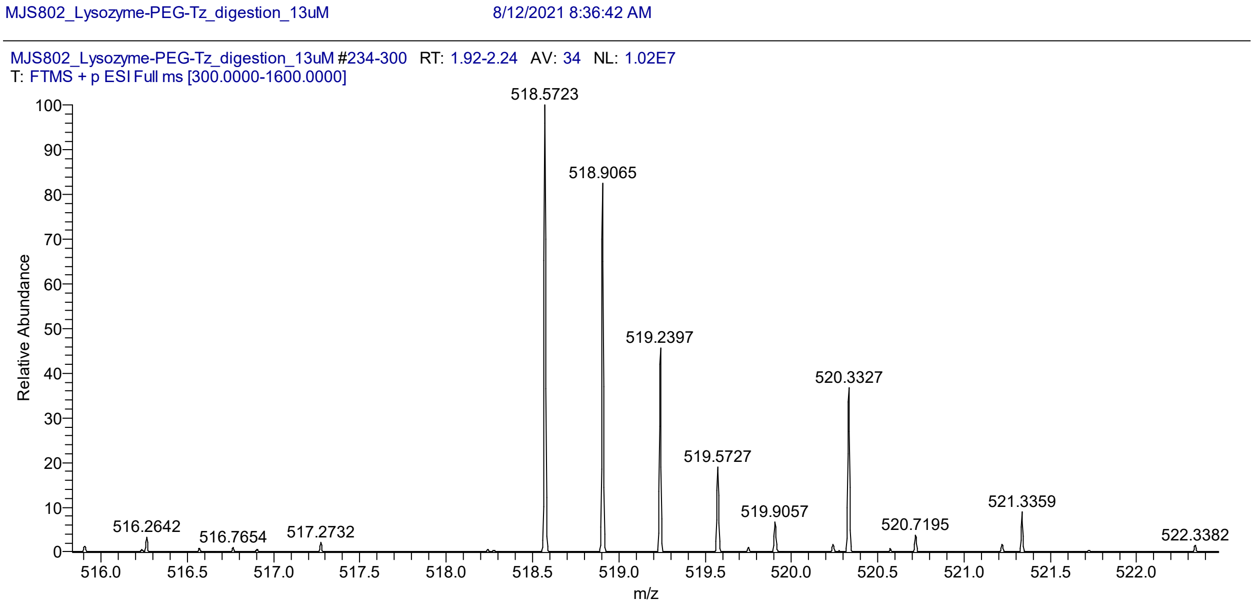

Fig. S53 Spectrum of peak at 2.0 min between 516-522 *m/z* range.

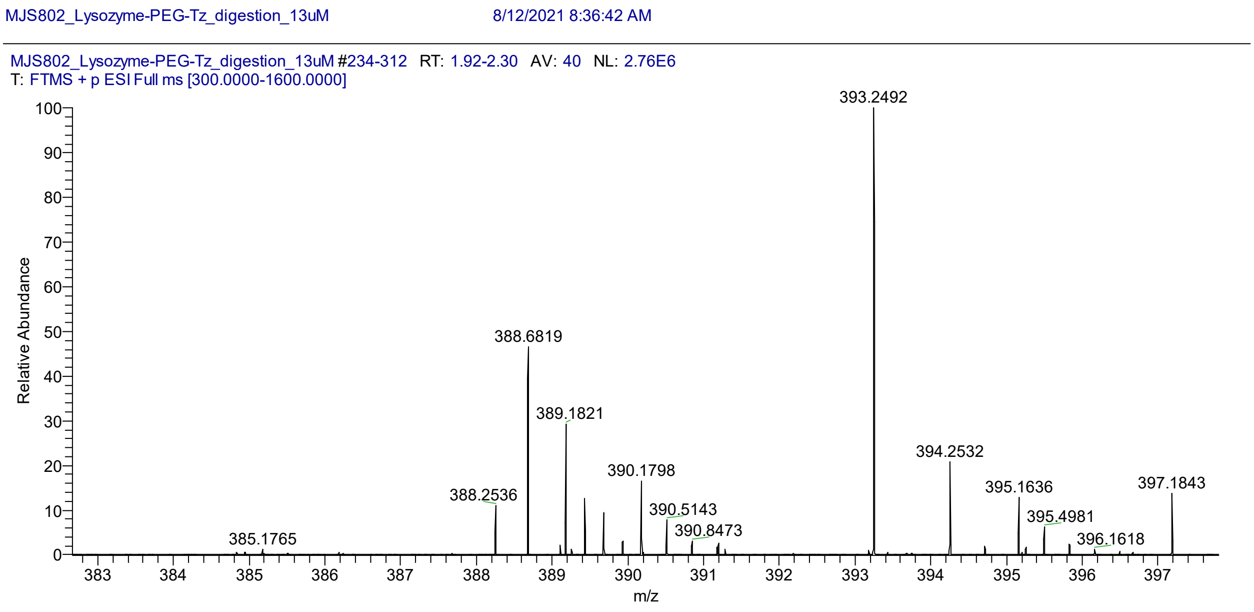

Fig. S54 Spectrum of peak at 2.0 min between 383-397 *m/z* range

LC–MS/MS analysis crosslink for C6 and C127

For the fragment sequence analysis five different sets of b and y ions were considered and searched in the SID 50eV spectrum of peak at 2.0 min. Found b ions are highlighted in blue, y ions in red and PEG-Tz fragment ions in green.

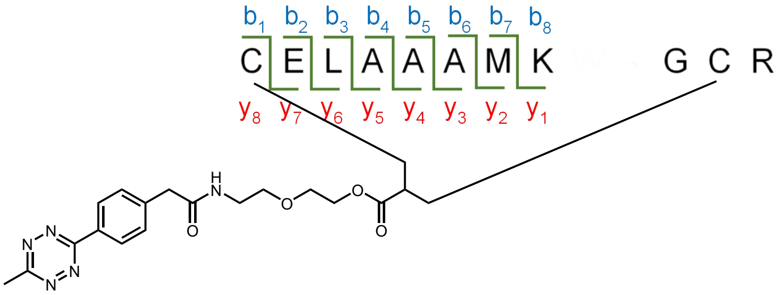

Lost fragment	#b	b⁺	Seq	y⁺	#y	Lost fragment
ELAAAMK	1*	821.3187	C	733.3913	7	C
LAAAMK	2	950.3613	E	604.3487	6	CE
AAAMK	3	1063.4453	L	491.2646	5	CEL
AAMK	4	1134.4825	A	420.2275	4	CELA
AMK	5	1205.5196	A	349.1904	3	CELAA
MK	6	1276.5567	A	278.1533	2	CELAAA
K	7	1407.5972	M	147.1128	1*	CELAAAM

Table S9 Expected b and y ions from AIF

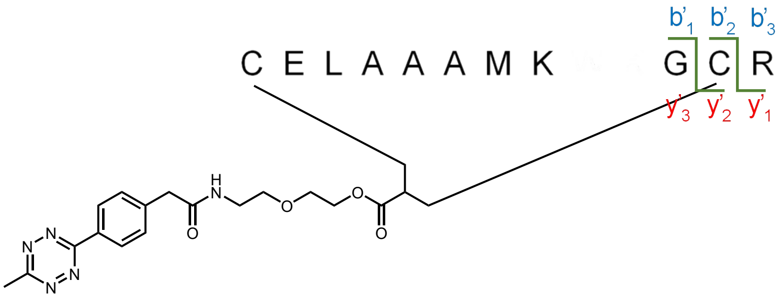

Lost fragment	#b'	b⁺	Seq	y⁺	#y'	Lost fragment
G	1	58.0287	G	1496.6807	2*	G
R	2*	1380.5978	C	175.1190	1	R

Table S10 Expected b' and y' ions from AIF

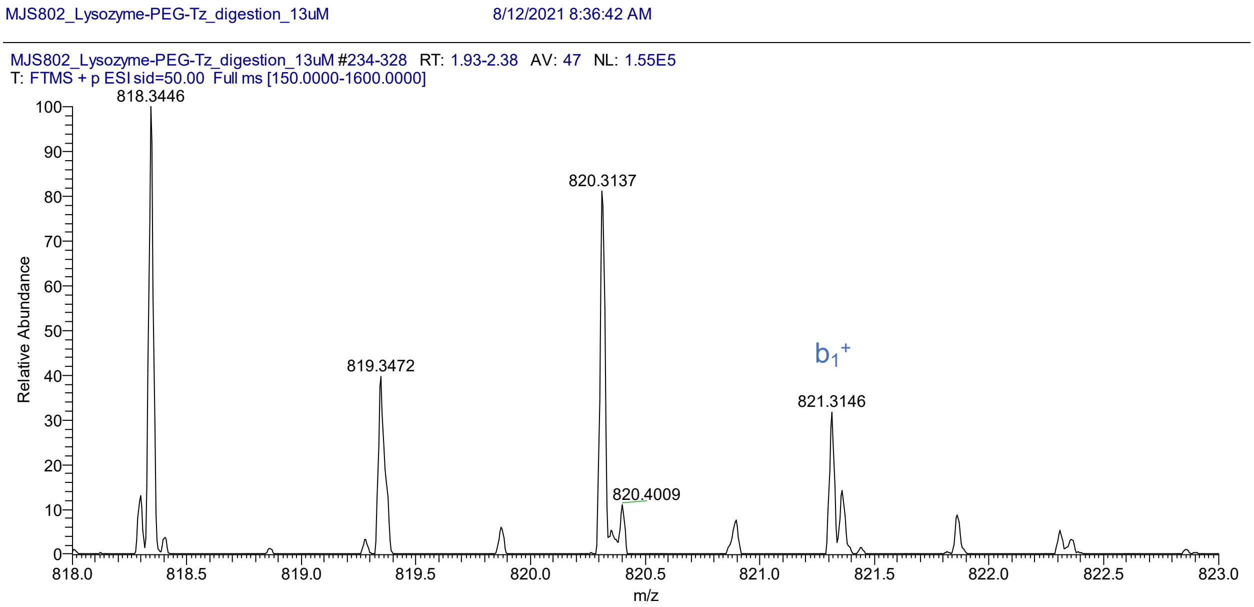

Fig. S55 AIF spectrum of peak at 2.0 min using SID 50 eV between 818-823 *m/z* range.

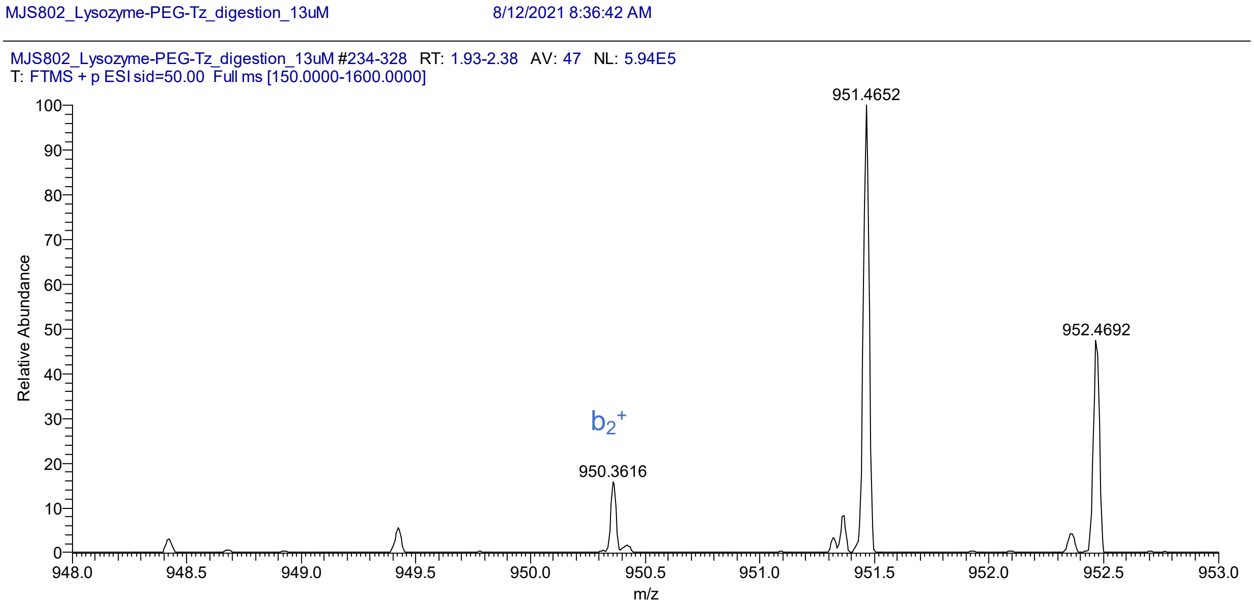

Fig. S56 AIF spectrum of peak at 2.0 min using SID 50 eV between 948-953 *m/z* range.

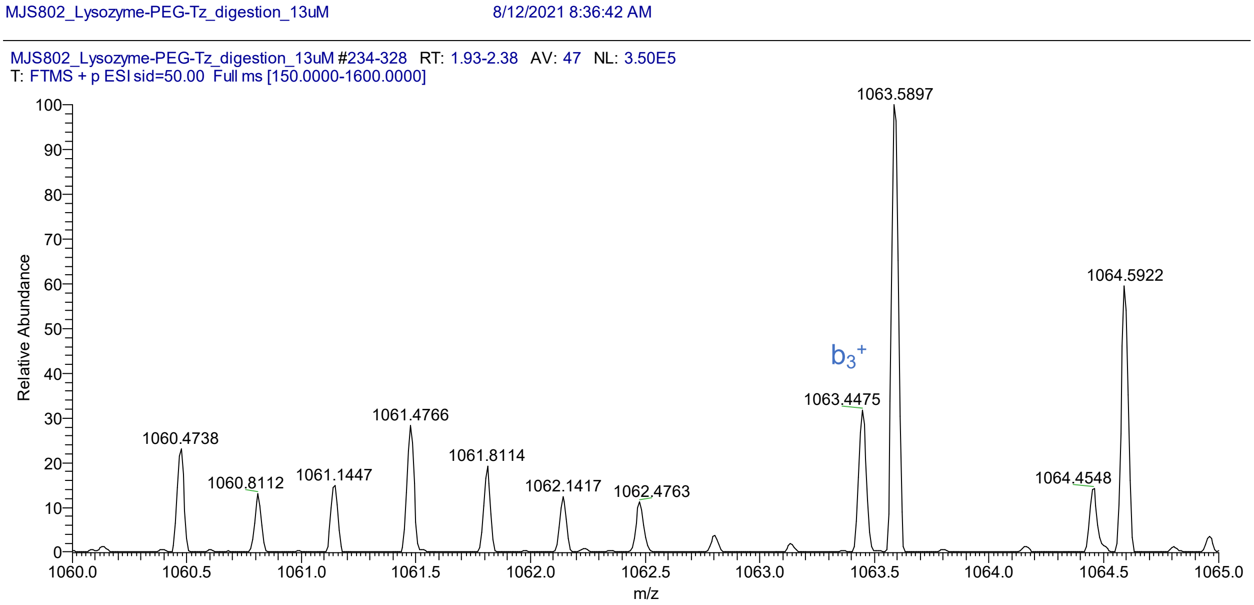

Fig. S57 AIF spectrum of peak at 2.0 min using SID 50 eV between 1060-1065 *m/z* range.

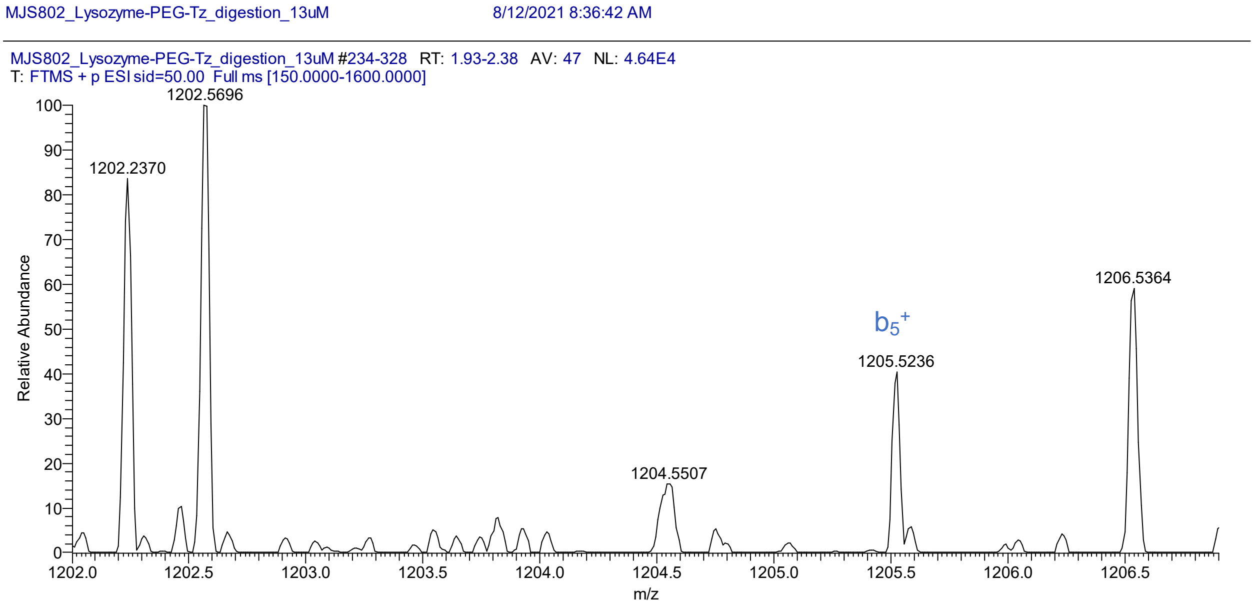

Fig. S58 AIF spectrum of peak at 2.0 min using SID 50 eV between 1202-1206.9 *m/z* range.

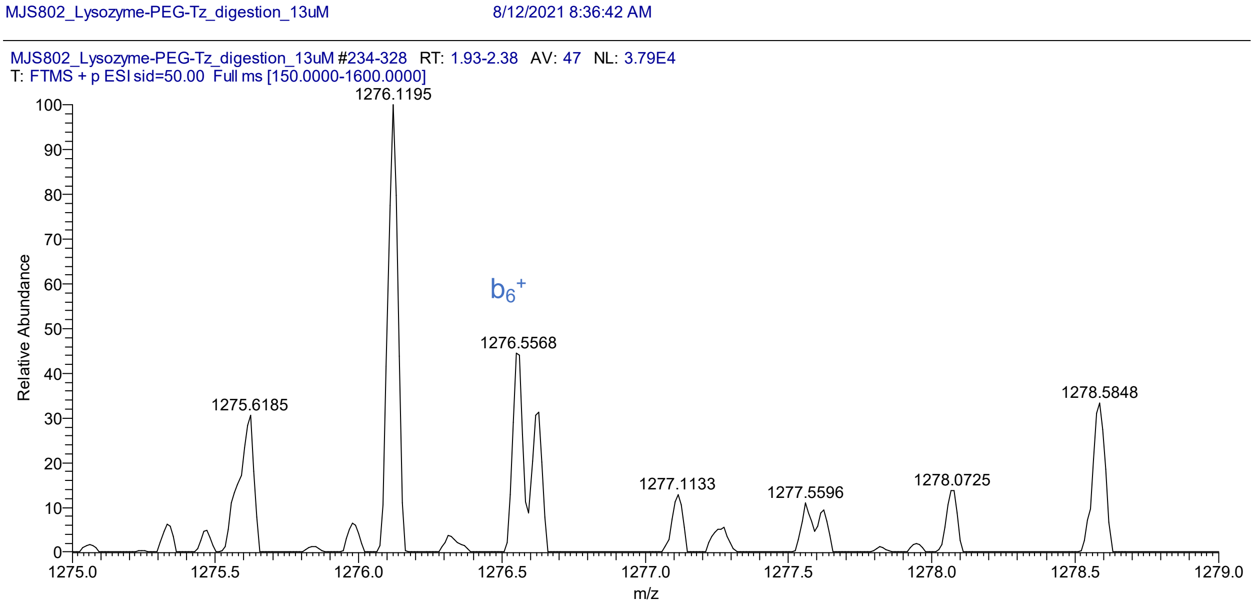

Fig. S59 AIF spectrum of peak at 2.0 min using SID 50 eV between 1275-1279 *m/z* range.

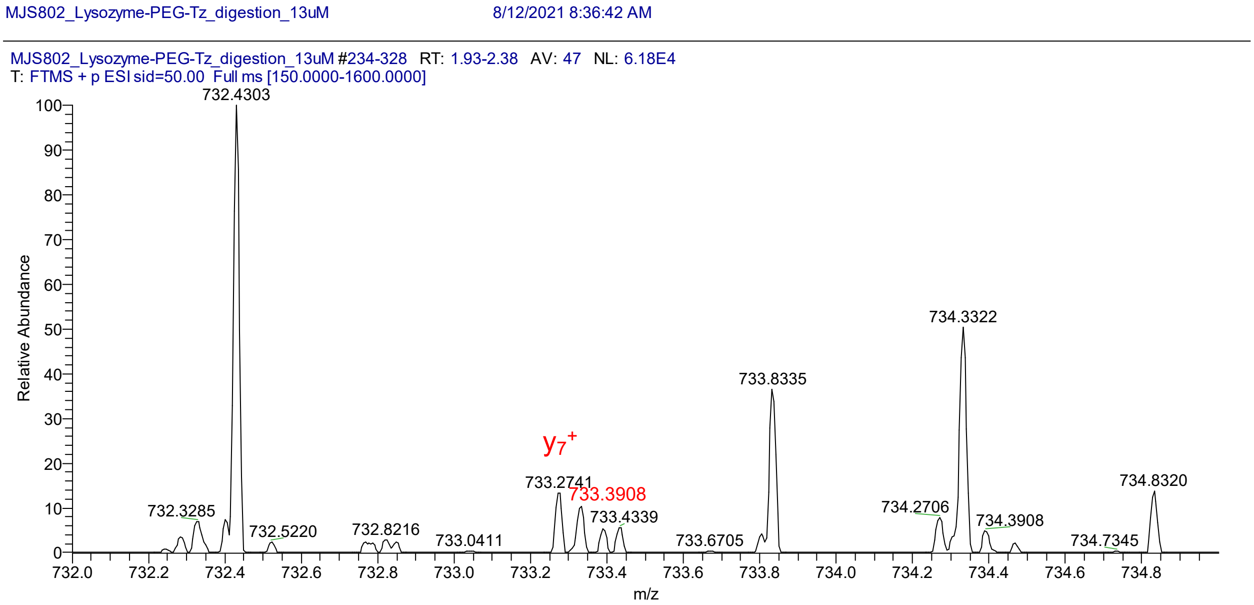

Fig. S60 AIF spectrum of peak at 2.0 min using SID 50 eV between 732-735 *m/z* range.

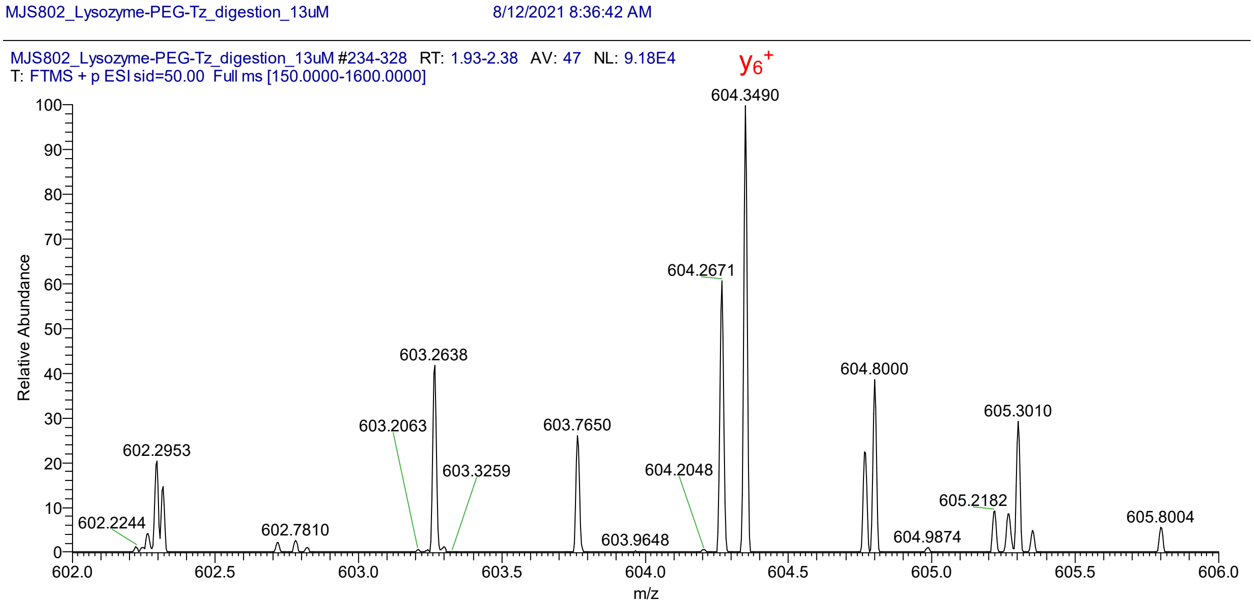

Fig. S61 AIF spectrum of peak at 2.0 min using SID 50 eV between 602-606 *m/z* range.

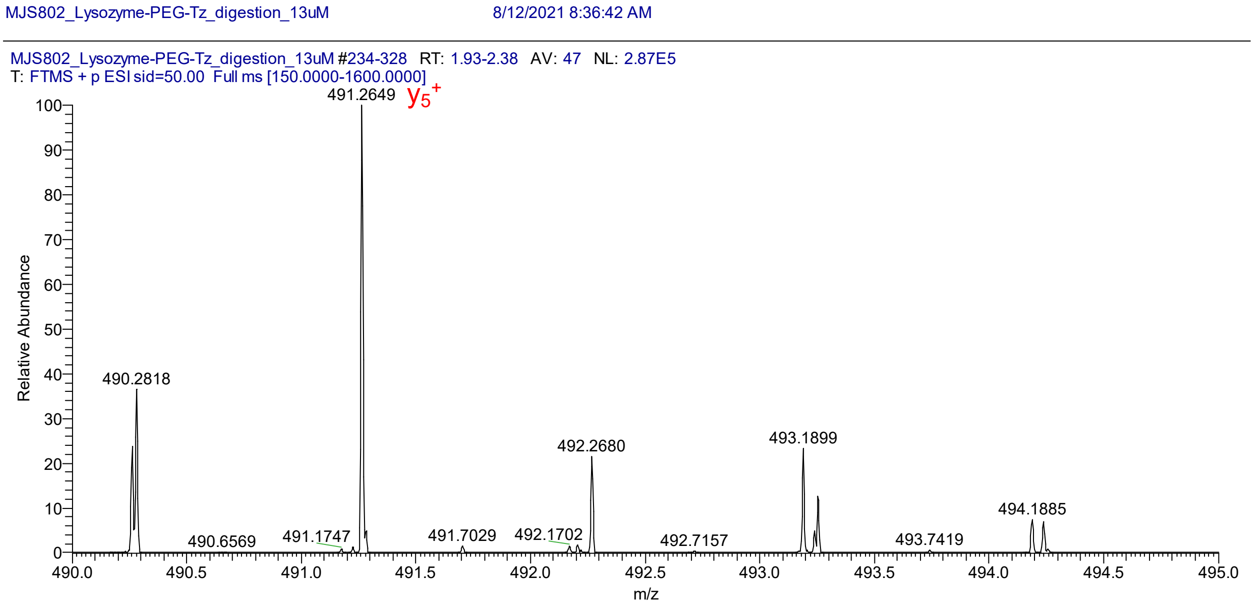

Fig. S62 AIF spectrum of peak at 2.0 min using SID 50 eV between 490-495 *m/z* range.

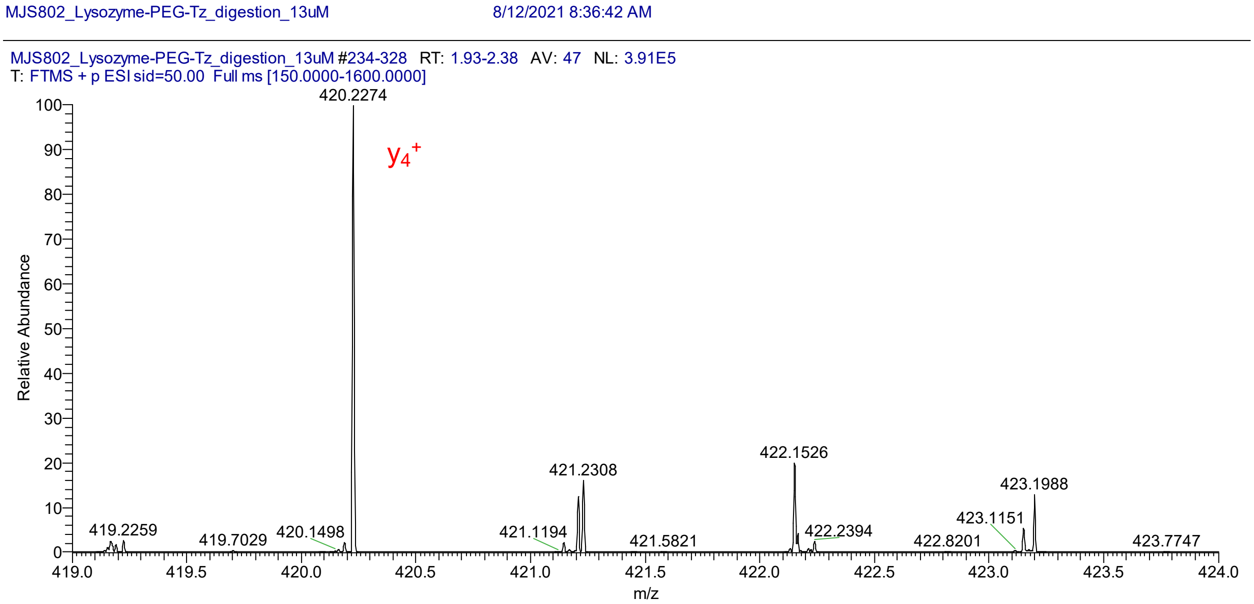

Fig. S63 AIF spectrum of peak at 2.0 min using SID 50 eV between 419-424 *m/z* range.

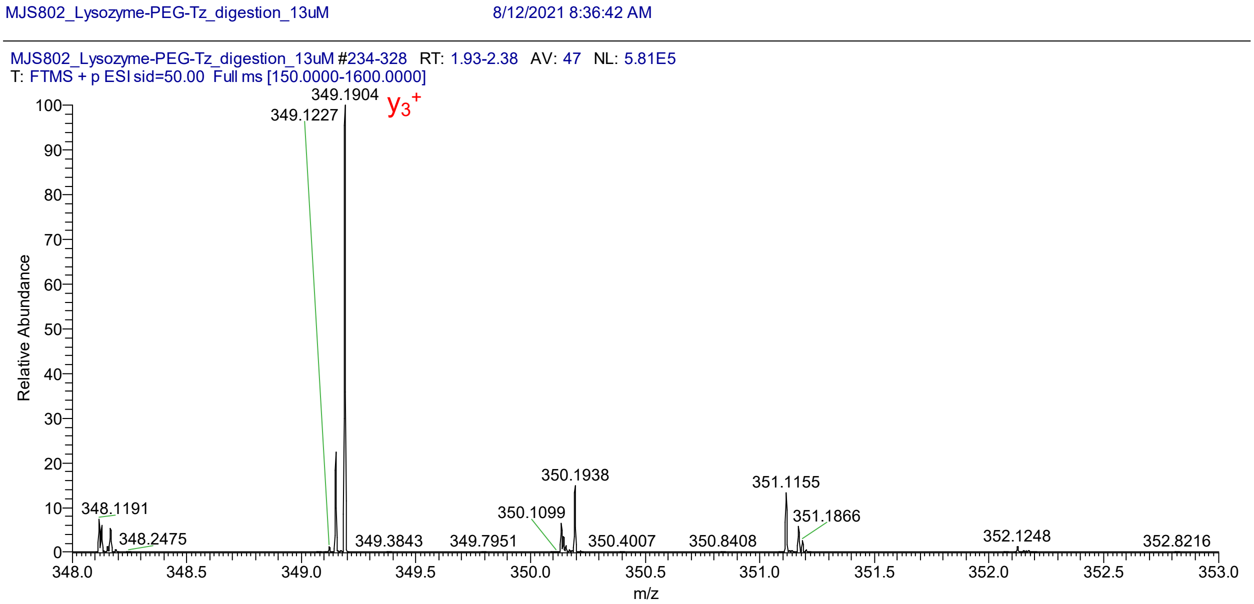

Fig. S64 AIF spectrum of peak at 2.0 min using SID 50 eV between 348-353 *m/z* range.

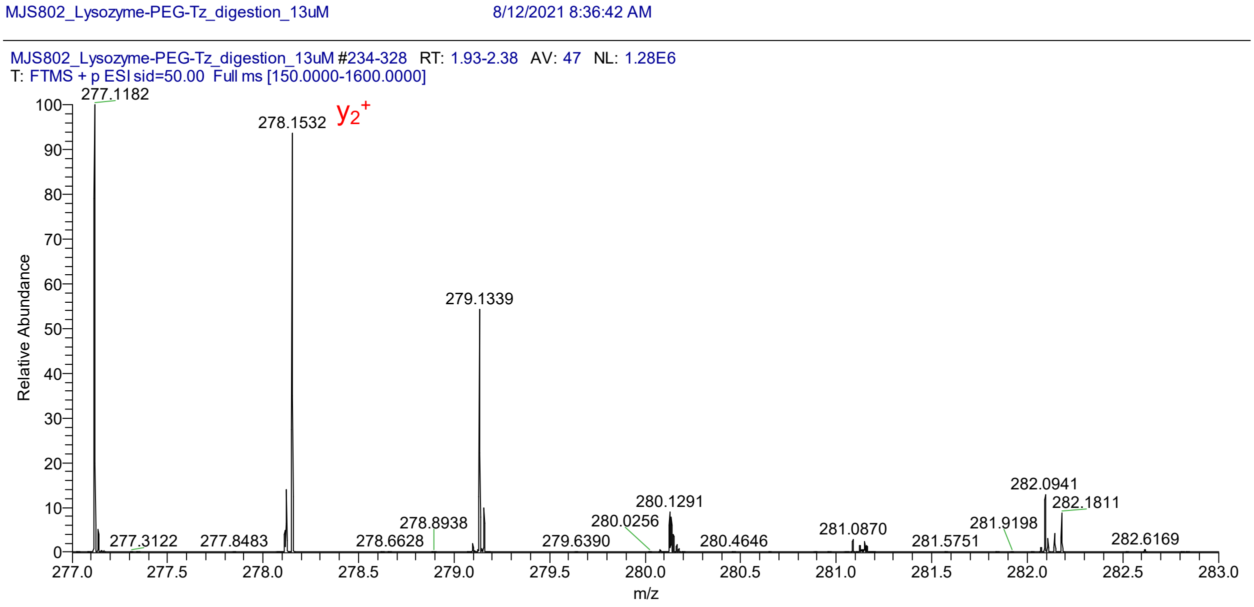

Fig. S65 AIF spectrum of peak at 2.0 min using SID 50 eV between 277-283 *m/z* range.

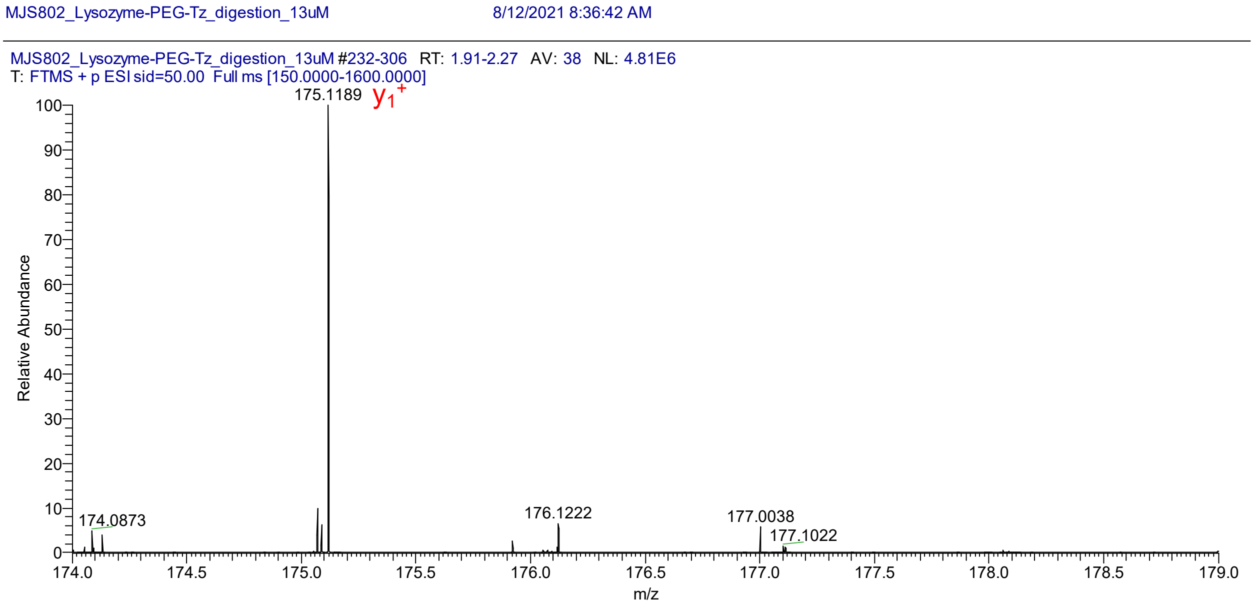

Fig. S66 AIF spectrum of peak at 2.0 min using SID 50 eV between 174-179 *m/z* range.

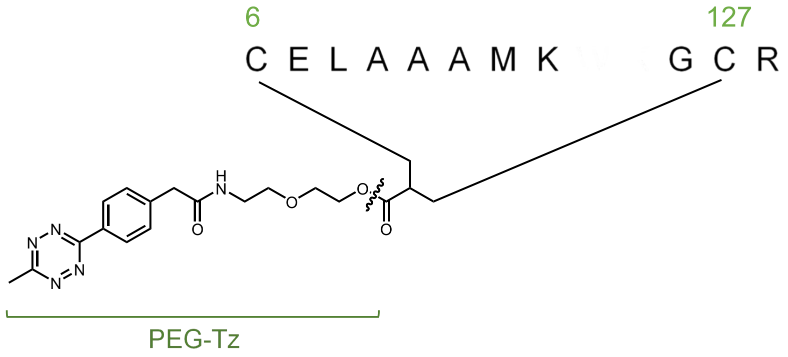

Fragment	Calculated *m/z*	Observed *m/z*
PEG-Tz	317.1482	NF
CELAAAMK + GCR + $[(CH_2)_2CHCO^{\cdot+}]$	1236.5534	1236.5533

Table S11 Expected ions after losing the PEG-Tz

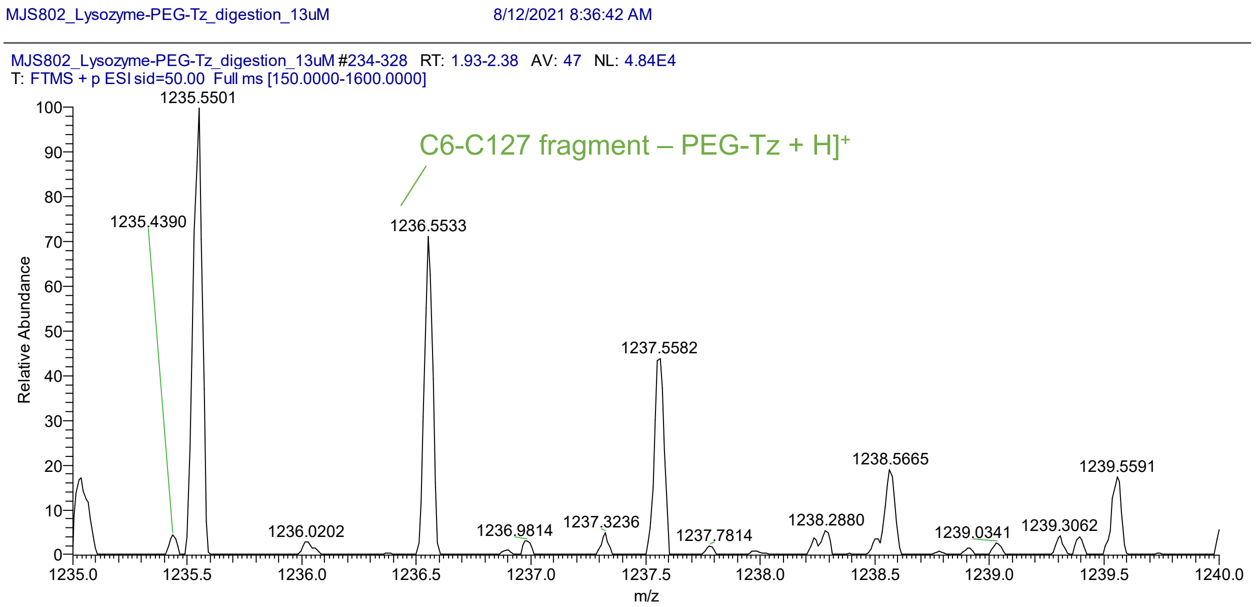

Fig. S67 AIF spectrum of peak at 2.0 min using SID 50 eV between 1235-1240 *m/z* range.

8.3 Statistical modification of lysozyme

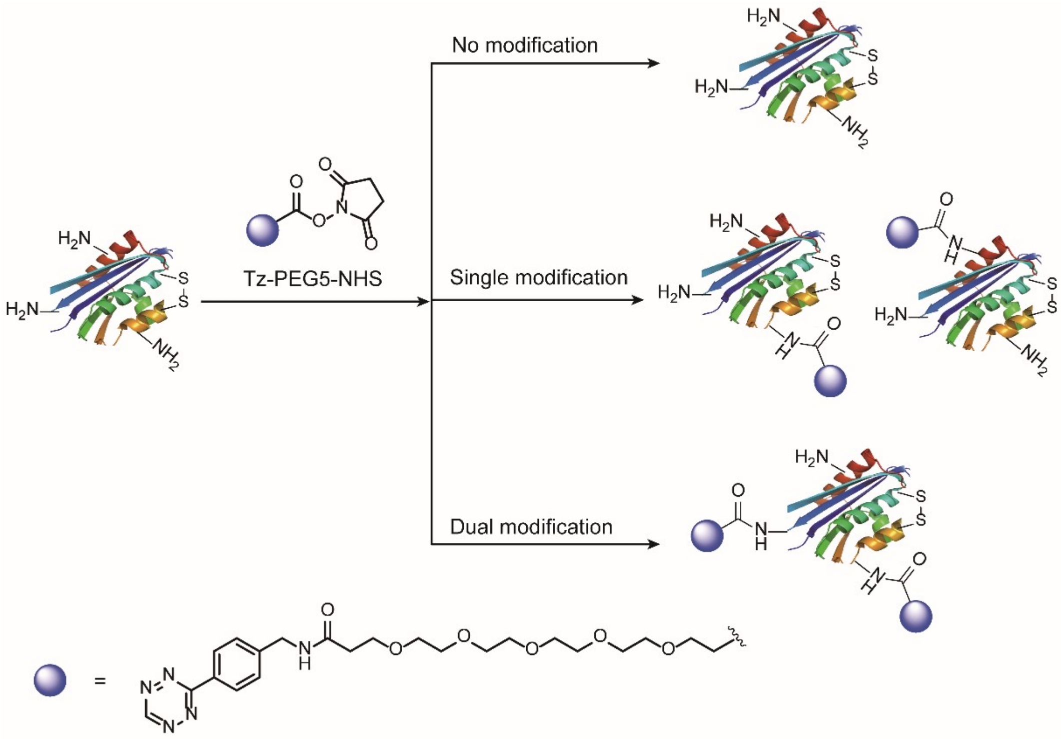

Scheme S13 Statistical modification of lysozyme with NHS ester chemistry.

Lysozyme (500 μg, 1 eq, 0.035 μmol) was dissolved in 500 μL PB buffer (50 mM, pH = 7.4). Next, Tz-PEG5-NHS (42 μg, 2 eq,.0.070 μmol) was added to the lysozyme solution and the mixture was stirred at room temperature for 4 h. Thereafter, the mixture was purified by ultrafiltration tube (cutoff: 5 kDa) to fully remove the DMSO and the excess organic molecules and change the solvent to water. The obtained solution was frozen with liquid nitrogen and water was removed with freeze dryer to afford a pink powder. The pink powder (Ly-PEG5-Tz) was characterized by MALDI-Tof-MS (Fig. S36). The MS data demonstrated that the statistical modification resulted in a mixture, which included the lysozyme with no modification, single and dual-modification.

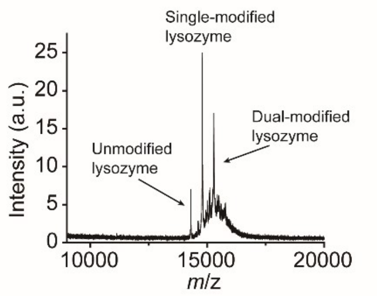

Fig. S67 MALDI-Tof-MS of Ly-PEG5-Tz after statistical modification based on the NHS ester chemistry.

8.4 Lysozyme enzymatic estimation

The lysozyme enzymatic assay was performed according to the protocol form Sigma Aldrich's technical bulletin. *Micrococcus lysodeikticus lyophilized cells* was suspended in 66 mM phosphate buffer with the pH at 6.2 with different concentrations, e.g. 0.5 mg/mL, 0.4 mg/mL, 0.3 mg/mL or 0.2 mg/mL. 250 μL of the cell suspensions was added to the 96-well plate and the absorbance was measured at 450 nm. According to the obtained absorbance, the concentration was carefully adjusted to ensure the absorbance at 450 nm between 0.6 and 0.7. In the experiment performed in this paper, the concentration was determined to be 0.32 mg/mL with the absorbance of around 0.65 at 450 nm. Ly-PEG-Tz was selected as a representative one to estimate its observed activity after modification. 250 μL of the cell suspensions was put in 3 wells of a 96-well plate. For the first well, 10 μL of phosphate buffer (66 mM, pH = 6.2) was added as a blank. To the second and third well, 10 μL of native lysozyme (0.01 mg/mL) and Ly-PEG-Tz (0.01 mg/mL) were added. The mixtures were pipetted up and down to fully mix the suspensions. Then the absorbance of each mixture was measured by Microplate reader (Tecan Spark 20 M) every minute for 15 minutes. The plotted graph was shown in Fig. 8b of the main text. The preserved activity of the modified lysozyme was estimated by comparing the slope of the plotted linear functions. The percent of the preserved activity of modified was calculated by dividing ($\frac{\Delta A_{450}}{min}$ (Ly-PEG-Tz) - $\frac{\Delta A_{450}}{min}$ (blank)) by ($\frac{\Delta A_{450}}{min}$ (native Lysozyme) - $\frac{\Delta A_{450}}{min}$ (blank)).

Samples	Blank	Native lysozyme	Ly-PEG-Tz
Slopes of the linear function	0.00046	-0.00985	-0.01148
$\frac{\Delta A_{450}}{min}$ (protein) - $\frac{\Delta A_{450}}{min}$ (blank)		0.01031	0.01194
Preserved enzymatic activity			86%

Similarly, the enzyme activity of the statistical modified lysozyme was also evaluated following the same protocol described above. The absorbance of each mixture was also measured by Microplate reader (Tecan Spark 20 M) every minute for 15 minutes. The preserved activity of the modified lysozyme was estimated by comparing the slope of the plotted linear functions. The plotted graph was shown in Fig. 8c. The percent of the preserved activity of modified was calculated by dividing ($\frac{\Delta A_{450}}{min}$ (Ly-PEG-Tz) - $\frac{\Delta A_{450}}{min}$ (blank)) by ($\frac{\Delta A_{450}}{min}$ (native Lysozyme) - $\frac{\Delta A_{450}}{min}$ (blank)).

Samples	Blank	Native lysozyme	Ly-PEG5-Tz
Slopes of the linear function	0.00047	-0.01027	0.00045
$\frac{\Delta A_{450}}{min}$ (protein) - $\frac{\Delta A_{450}}{min}$ (blank)		0.01074	-0.00002
Preserved enzymatic activity			~ 0%

8.5 Circular dichroism

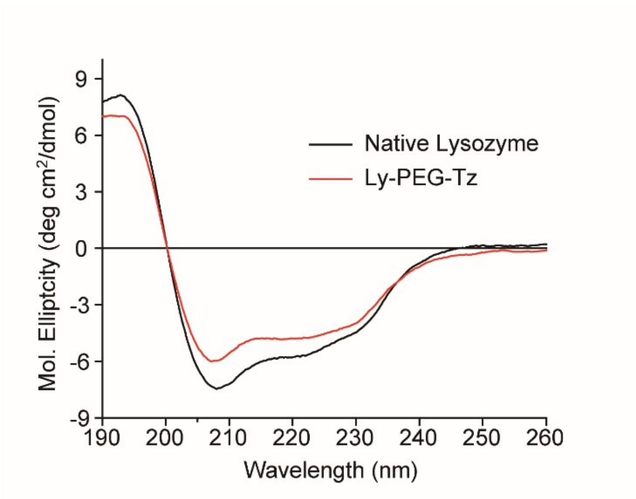

Fig. S68 CD spectra of native lysozyme and modified lysozyme from 190 nm to 260 nm.

9. Summary and comparison the disulfide modification strategy

Reagents	Synthetic steps	Conjugation Yield	Reaction time	log P	Reaction conditions
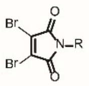 Dibromomaleimide	Two steps	>80 (Determined by SDS-PAGE and MS spectrum)	4 h	0.1	Borate buffered saline (50 mM, pH = 7) 2 eq conjutation reagent
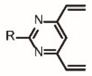 Divinylpyrimidine	Three steps	Good conversion (Determined by MS spectrum)	2 h	3.13	Tris buffer (25 mM, pH = 8) 15 eq conjutation reagent
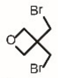 Oxetane	Commerical (No possibility for post-functionalization)	> 95% (Dertermined by MS spectrum)	24 h	1.63	Phosphate buffer (100 mM, pH = 9) 20 eq conjugation reagent
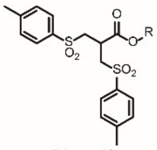 Bis-sulfone	Three steps	around 20% Isolated yield	Overnight	2.4	Phosphate buffer (100 mM, pH = 7.8) 2 eq conjugation reagent
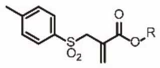 Allyl sulfone	Four steps	19% Isolated yield	Overnight	1.6	Phosphate buffer (50 mM, pH = 7.8) 2 eq conjugation reagent
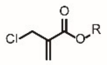 2-Chrlomethyl acrylate	One step	30% Isolated yield	Overnight	1.15	Phosphate buffer (50 mM, pH = 7) 1.2 eq conjugation reagent

Table S12 A brief summary of the current available disulfide modification strategies reported in the literature with respect to the synthetic steps, conjugation yield, reaction time, log P as well as reaction conditions.

As shown in Table S12, the disulfide strategy developed in this work bears the advantages of easy preparation (one-step synthesis), good water solubility (rather low log P) and relatively efficient conjugation with using a

little bit excess of rebridging reagent.

10. LC-MS of the synthesized compounds

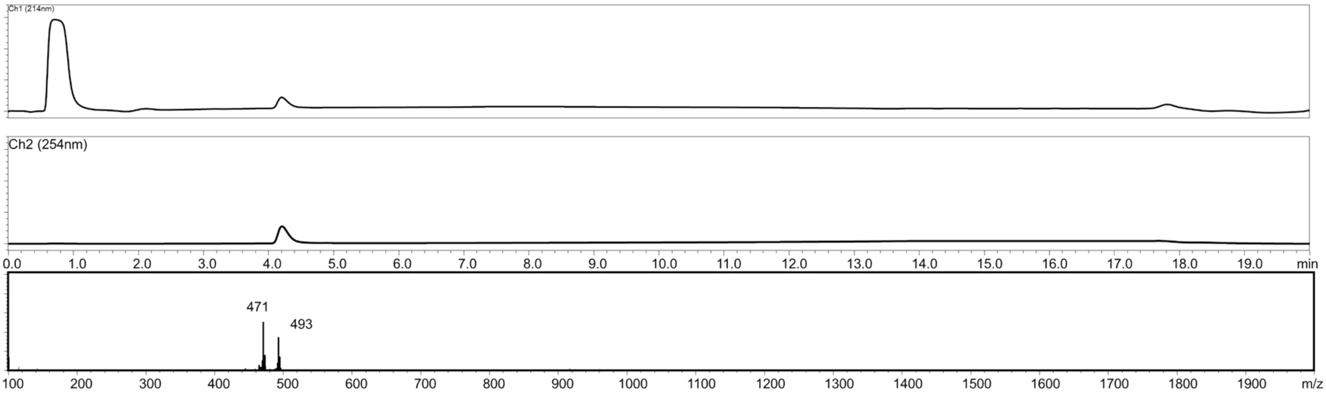

Fig. S69 LC-MS of compound **2** (calculated: 471[M+H]+, 493 [M+Na]+; found: 471 [M+H]+, 493 [M+Na]+)

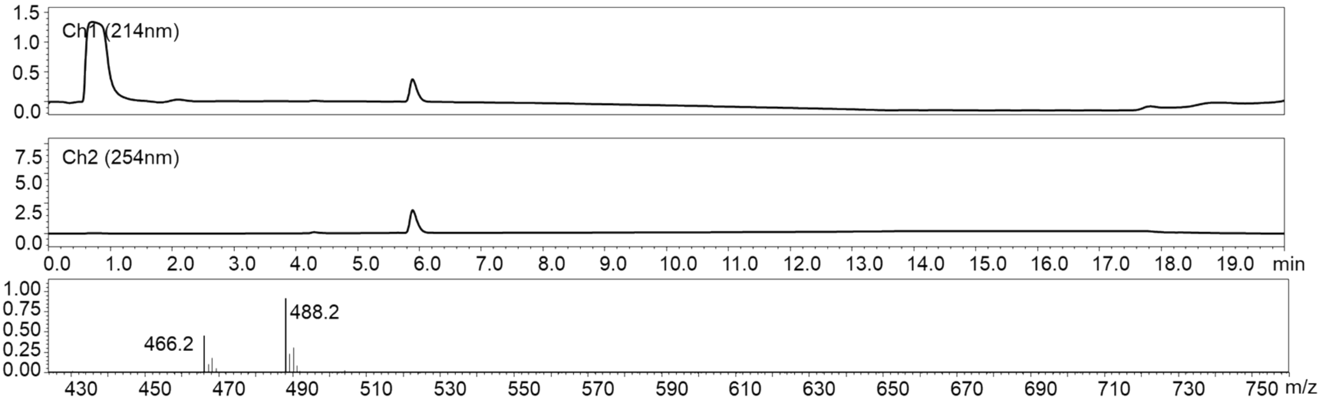

Fig. S70 LC-MS of compound **3** (calculated: 466 [M+H]+, 488 [M+Na]+; founded: 466 [M+H]+, 488 [M+Na]+)

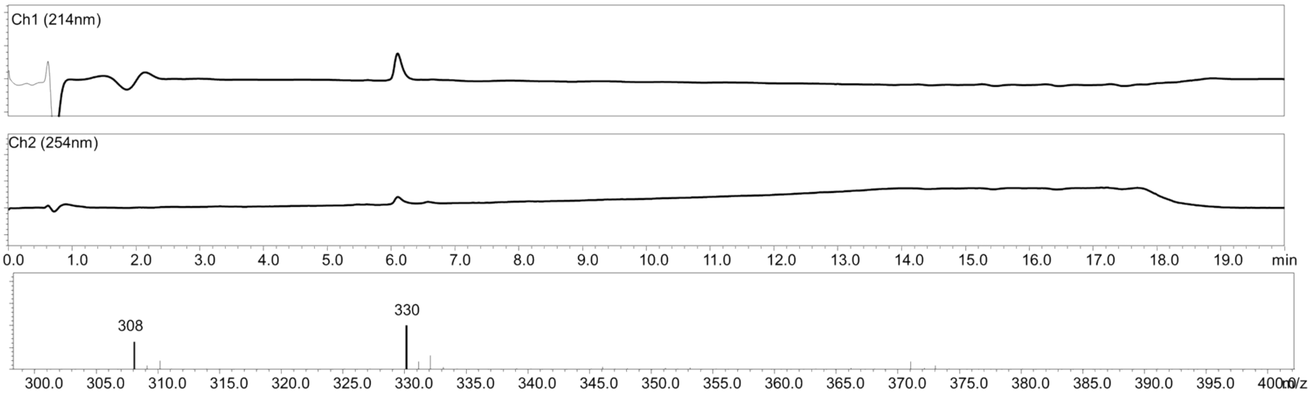

Fig. S71 LC-MS of compound **5** (calculated: 308 [M+H]$^+$, 330 [M+Na]$^+$; found: 308 [M+H]$^+$, 330 [M+Na]$^+$)

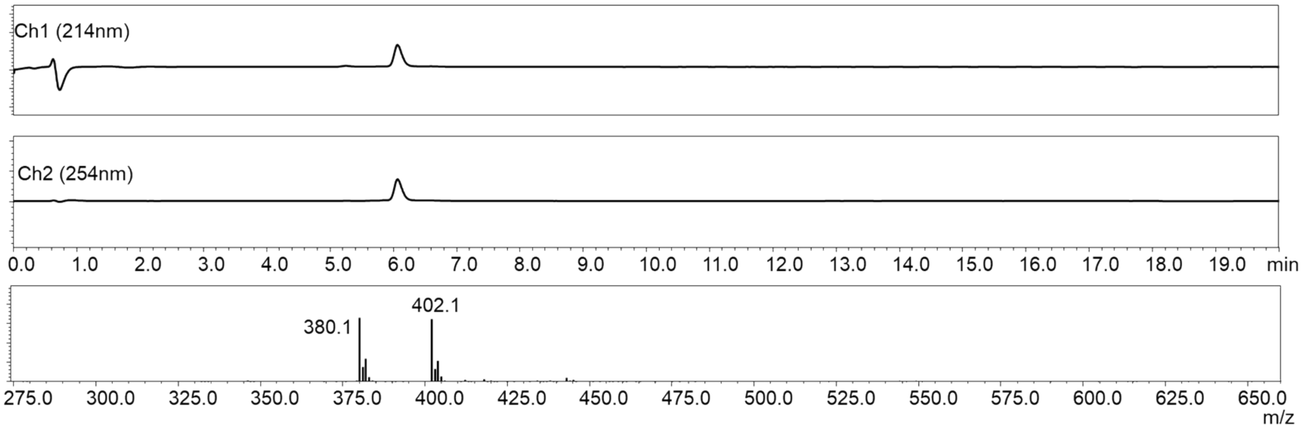

Fig. S72 LC-MS of compound **6** (calculated: 380 [M+H]$^+$, 402 [M+Na]$^+$; found: 380 [M+H]$^+$, 402 [M+Na]$^+$)

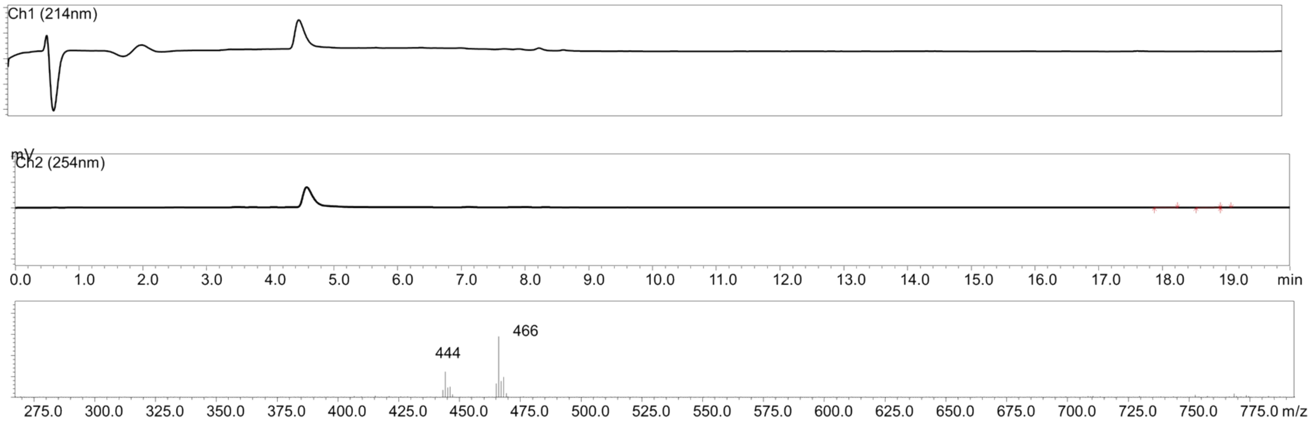

Fig. S73 LC-MS of compound **7** (calculated: 444 [M+H]$^+$, 466 [M+Na]$^+$; found: 444 [M+H]$^+$, 466 [M+Na]$^+$)

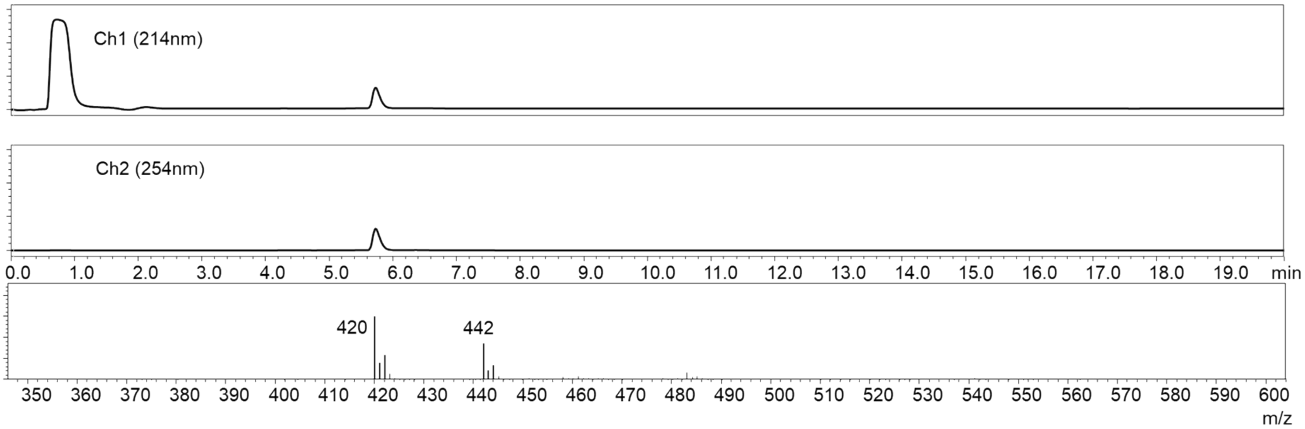

Fig. S74 LC-MS of compound **8** (calculated: 420 [M+H]$^+$, 442 [M+Na]$^+$; found: 420 [M+H]$^+$, 442 [M+Na]$^+$)

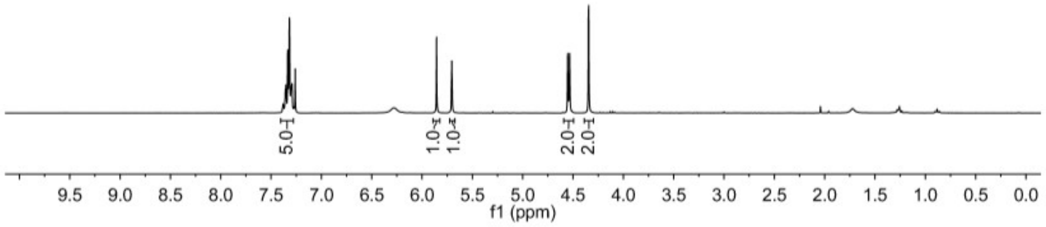

Fig. S75 ^{1}H NMR of compound **1** ($CDCl_3$, 298K, 300 MHz)

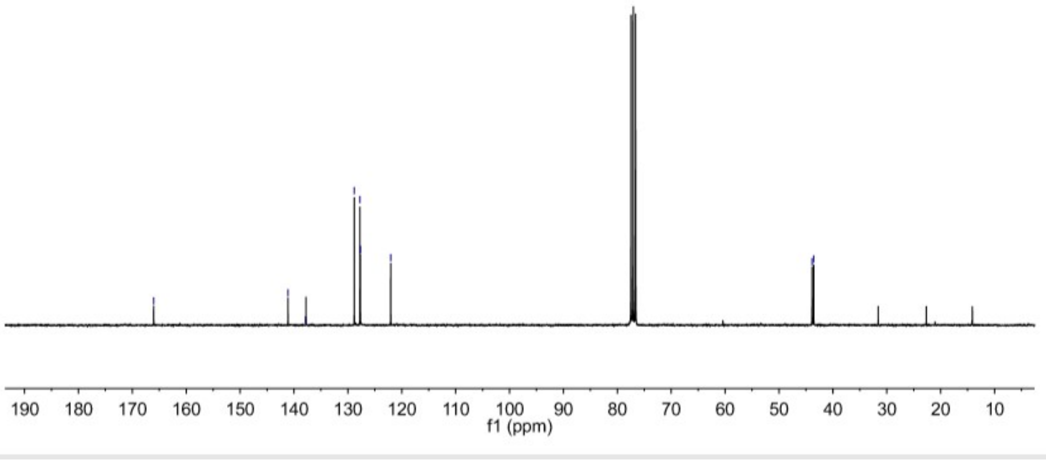

Fig. S76 ^{13}C NMR of Compound **1**[1] ($CDCl_3$, 298K, 75 MHz)

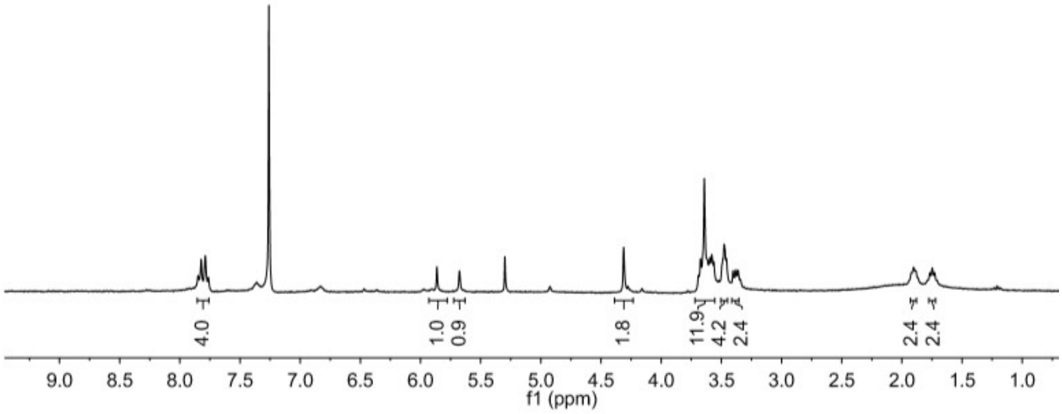

Fig. S77 ^{1}H NMR of compound **2** ($CDCl_3$, 298K, 300 MHz)

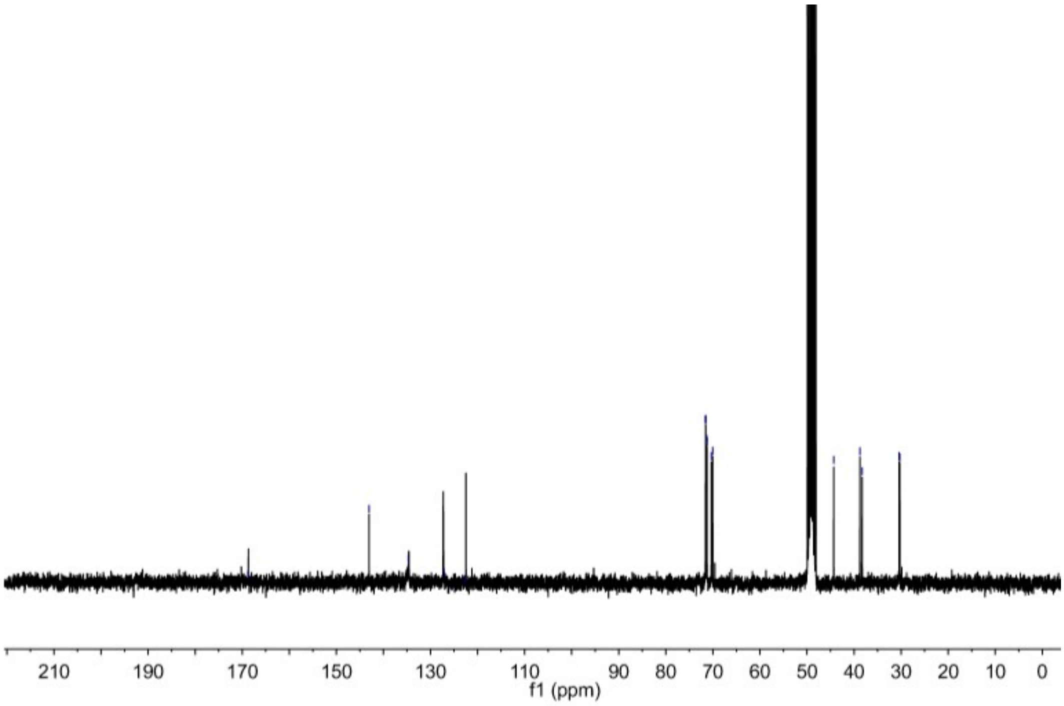

Fig. S78 ^{13}C NMR of compound **2** (CD_2Cl_2, 298K, 75 MHz)

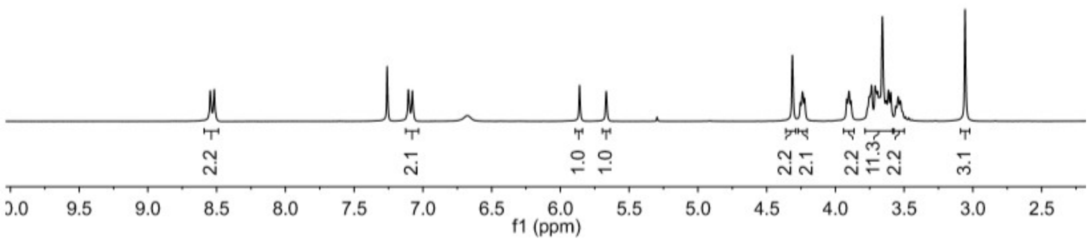

Fig. S79 ^{1}H NMR of compound **3** ($CDCl_3$, 298K, 300 MHz)

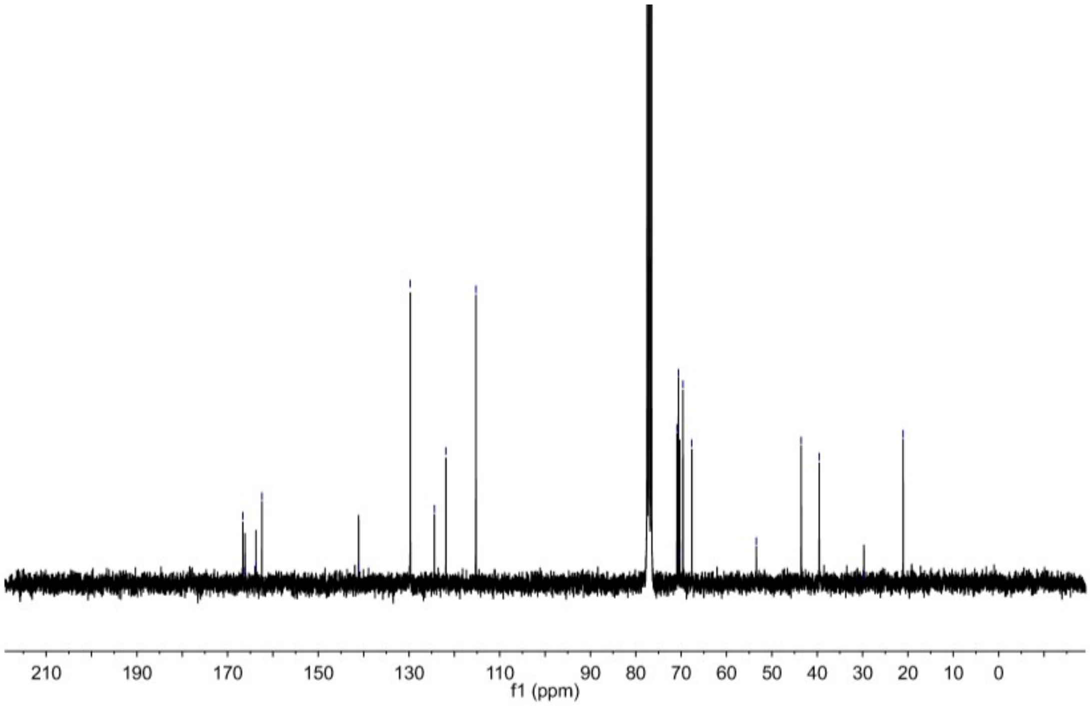

Fig. S80 ^{13}C NMR of compound **3** ($CDCl_3$, 298K, 75 MHz)

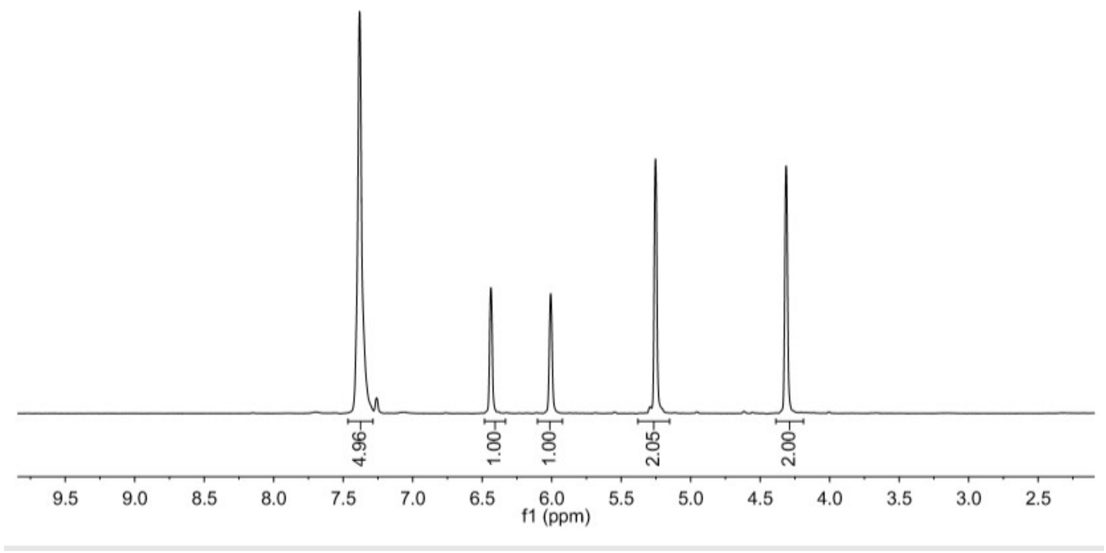

Fig. S81 ^{1}H NMR of compound **4** ($CDCl_3$, 298K, 300 MHz)

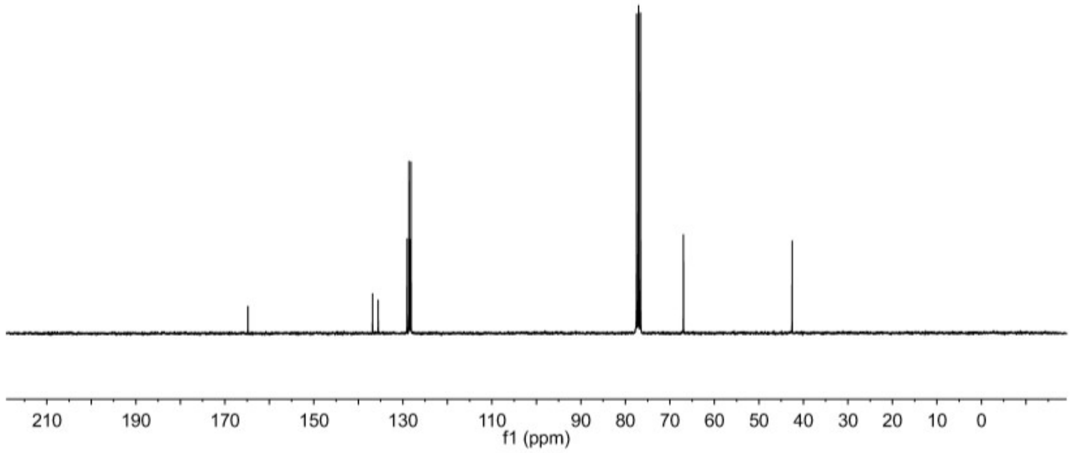

Fig. S82 ^{13}C NMR of compound **4** ($CDCl_3$, 298K, 75 MHz)

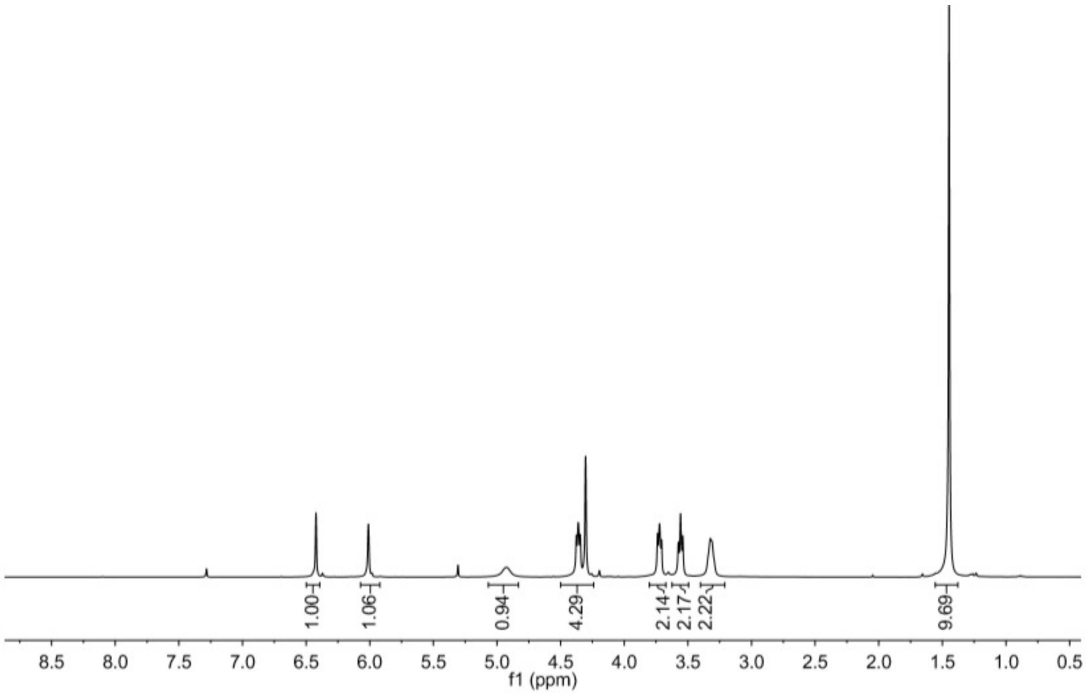

Fig. S83 ^{1}H NMR of compound **5** ($CDCl_3$, 298K, 300 MHz)

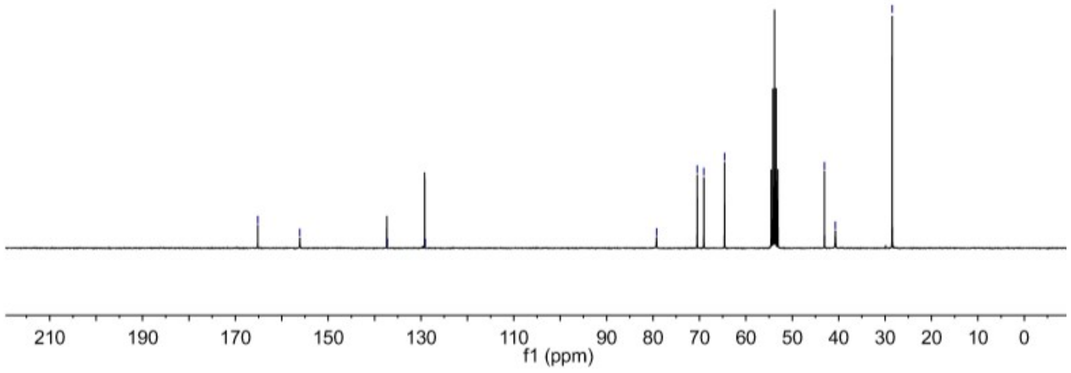

Fig. S84 ^{13}C NMR of compound **5** (CD_2Cl_2, 298K, 75 MHz)

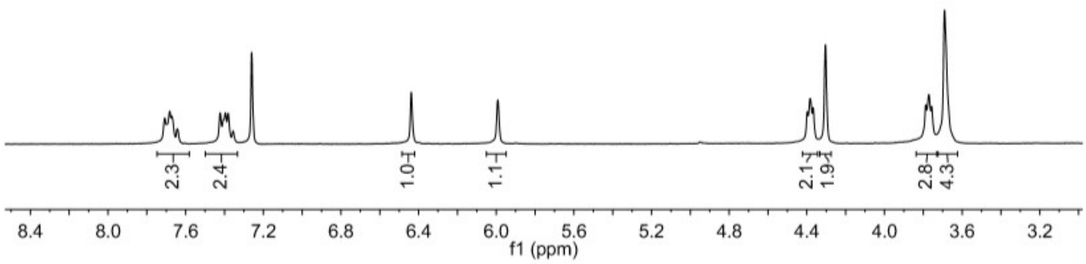

Fig. S85 ^{1}H NMR of compound **6** ($CDCl_3$, 298K, 300 MHz)

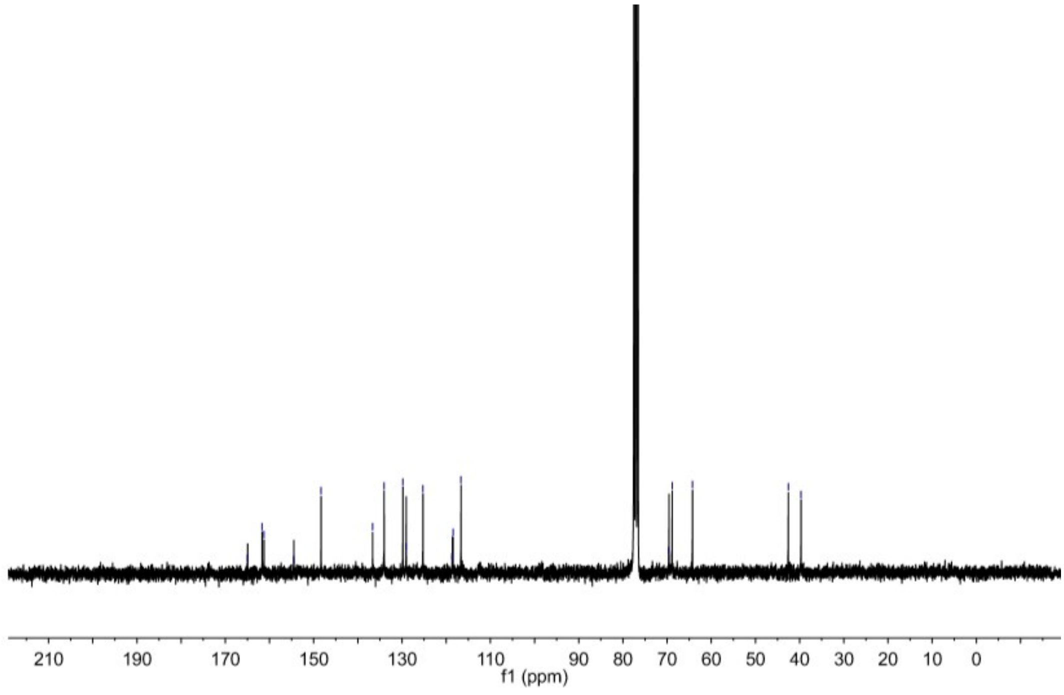

Fig. S86 ^{13}C NMR of compound **6** ($CDCl_3$, 298K, 75 MHz)

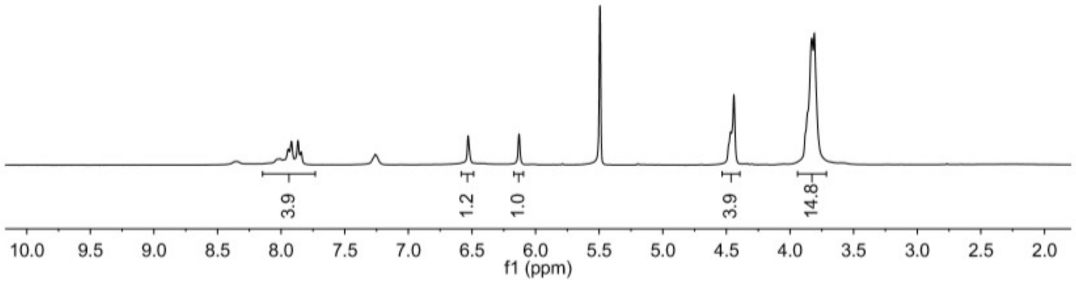

Fig. S87 ^{1}H NMR of compound **7** (CD_2Cl_2, 298K, 300 MHz)

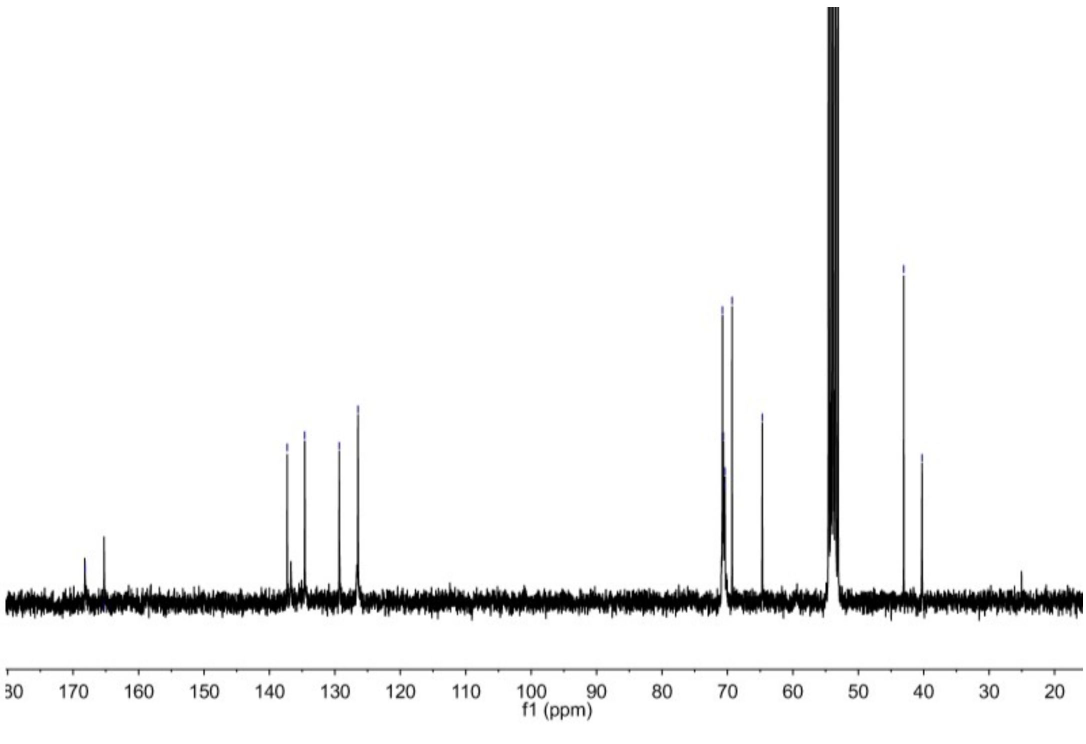

Fig. S88 ^{13}C NMR of compound **7** (CD_2Cl_2, 298K, 75 MHz)

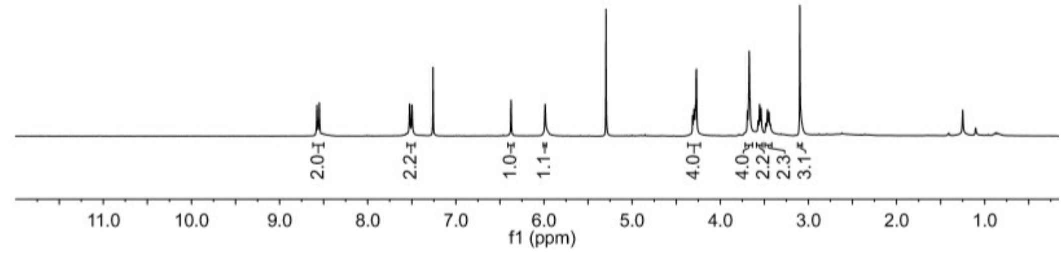

Fig. S89 ^{1}H NMR of compound **8** ($CDCl_3$, 298K, 300 MHz)

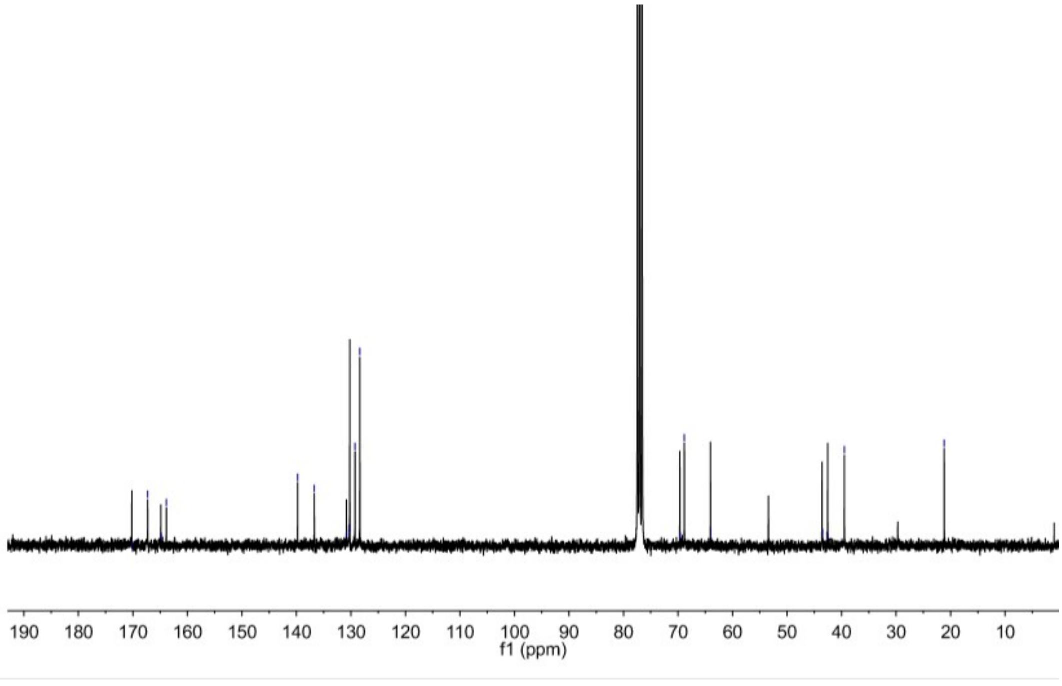

Fig. S90 ^{13}C NMR of compound **8** ($CDCl_3$, 298K, 75 MHz)

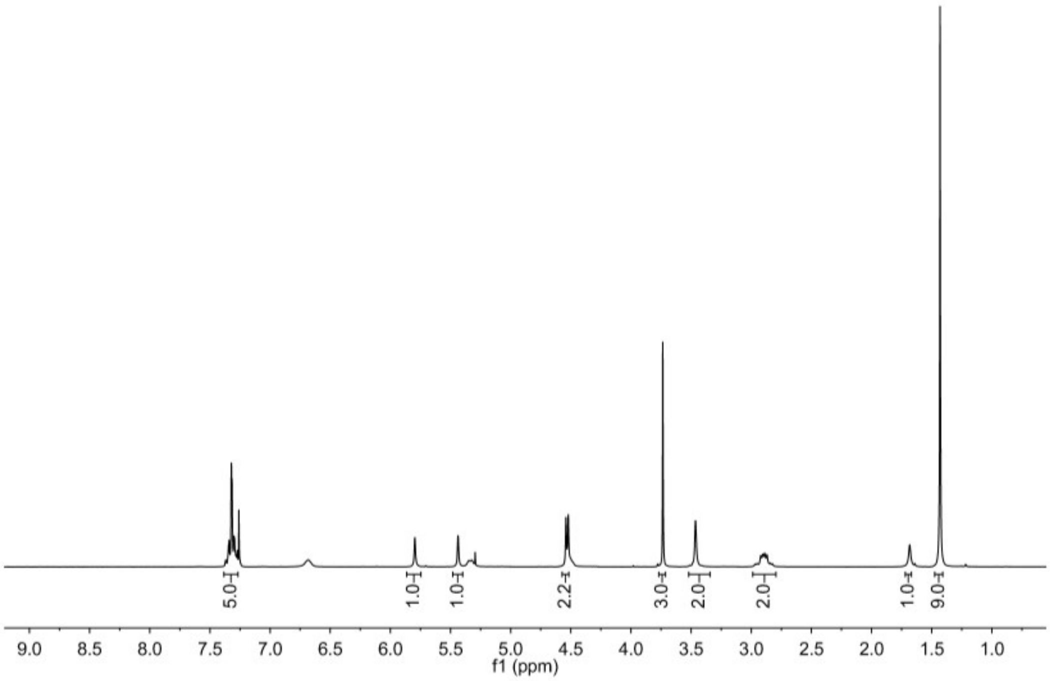

Fig. S91 ^{1}H NMR of compound **10** ($CDCl_3$, 298K, 300 MHz)

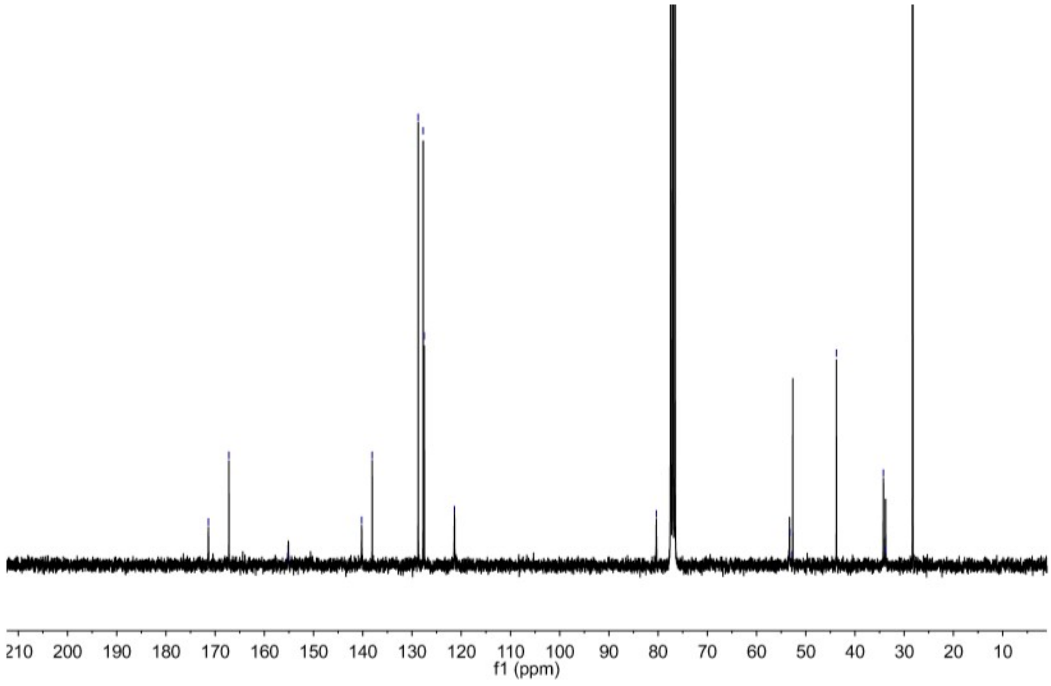

Fig. S92 ^{13}C NMR of compound **10** ($CDCl_3$, 298K, 75 MHz)

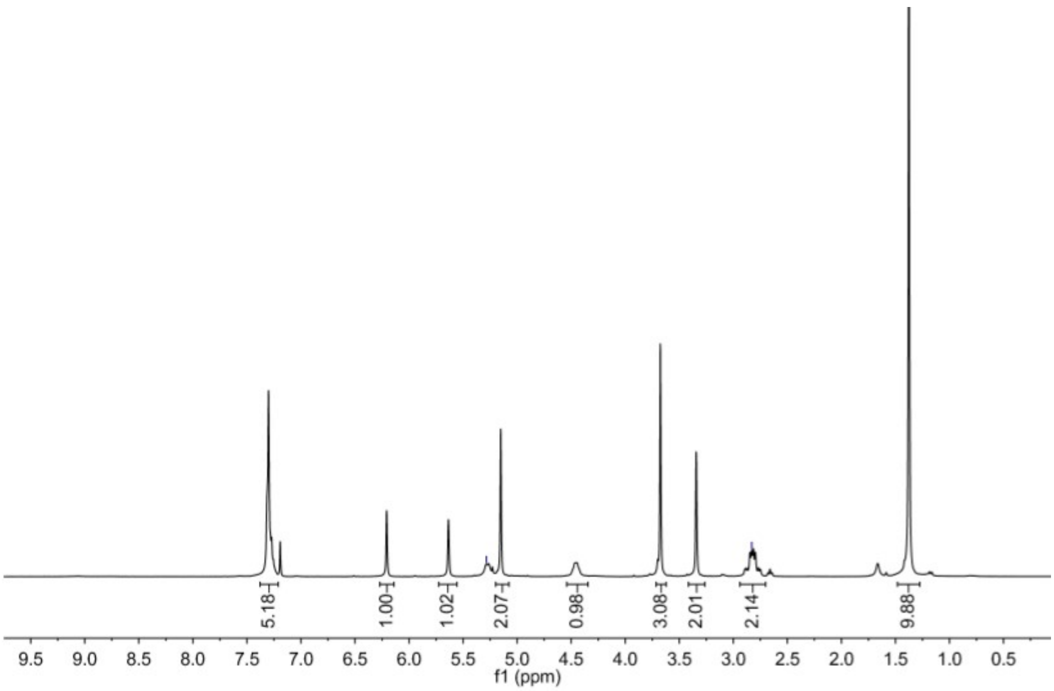

Fig. S93 ^{1}H NMR of compound **12** ($CDCl_3$, 298K, 300 MHz)

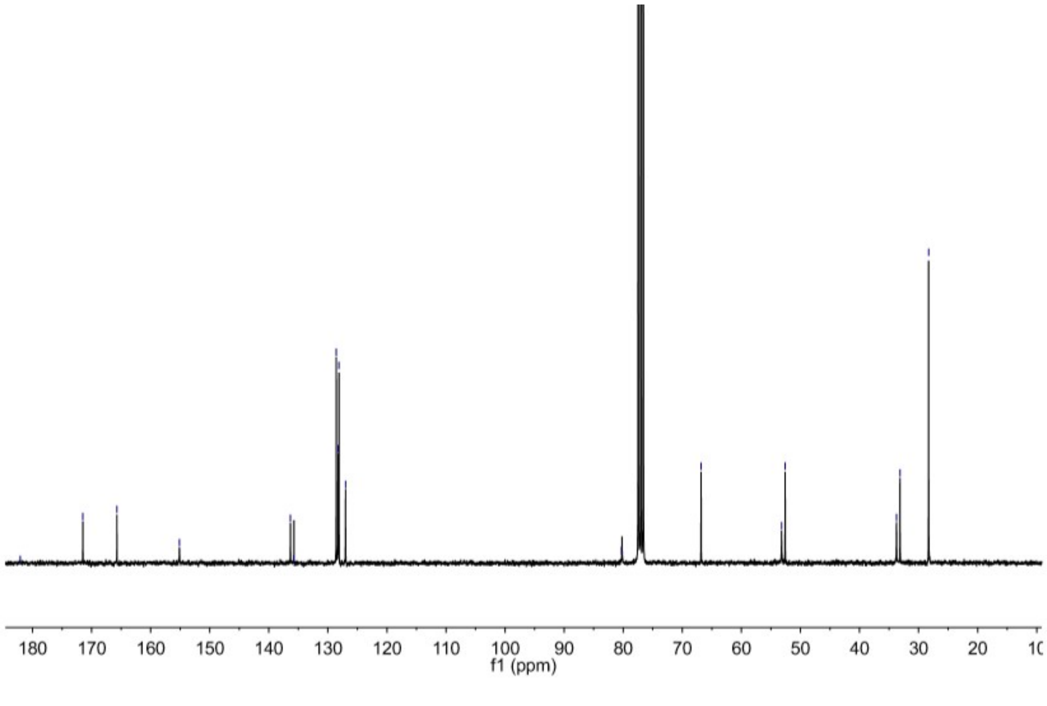

Fig. S94 ^{13}C NMR of compound **12** ($CDCl_3$, 298K, 75 MHz)

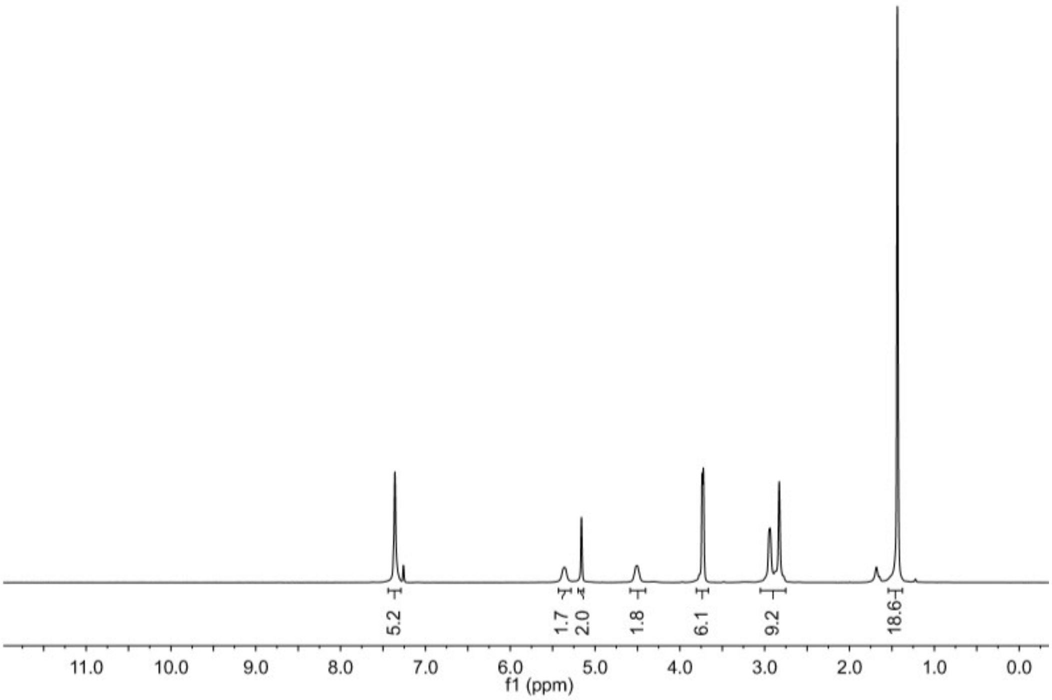

Fig. S95 ^{1}H NMR of compound **13** ($CDCl_3$, 298K, 300 MHz)

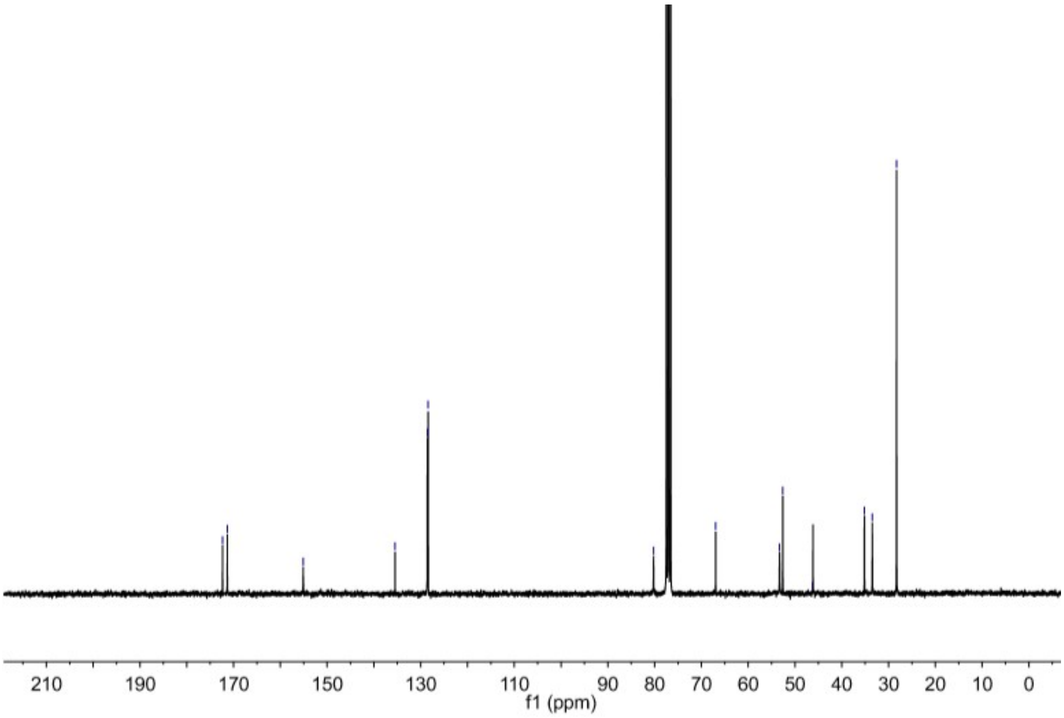

Fig. S96 ^{13}C NMR of compound **13** ($CDCl_3$, 298K, 75 MHz)

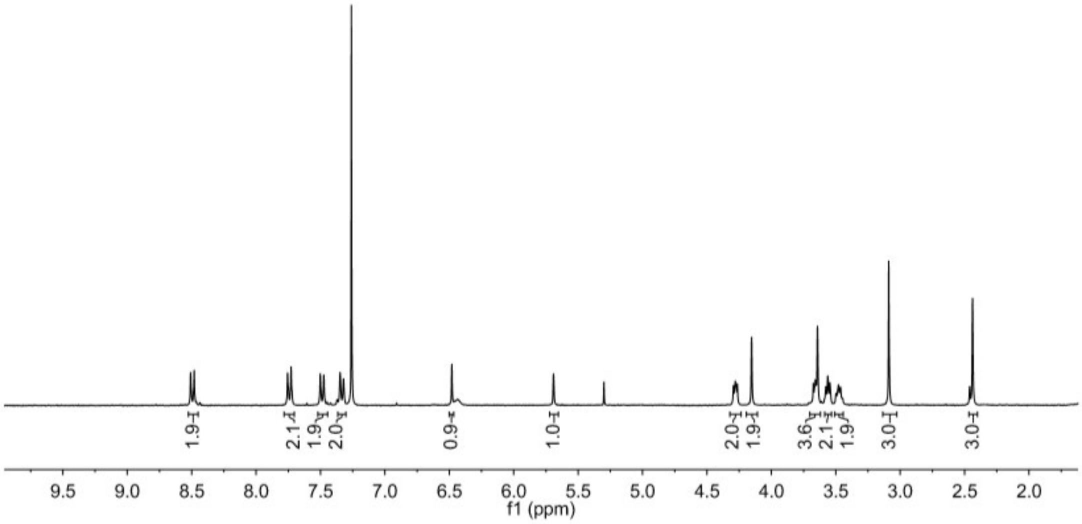

Fig. S97 ^{1}H NMR of compound **IC-Tz** ($CDCl_3$, 298K, 300 MHz)

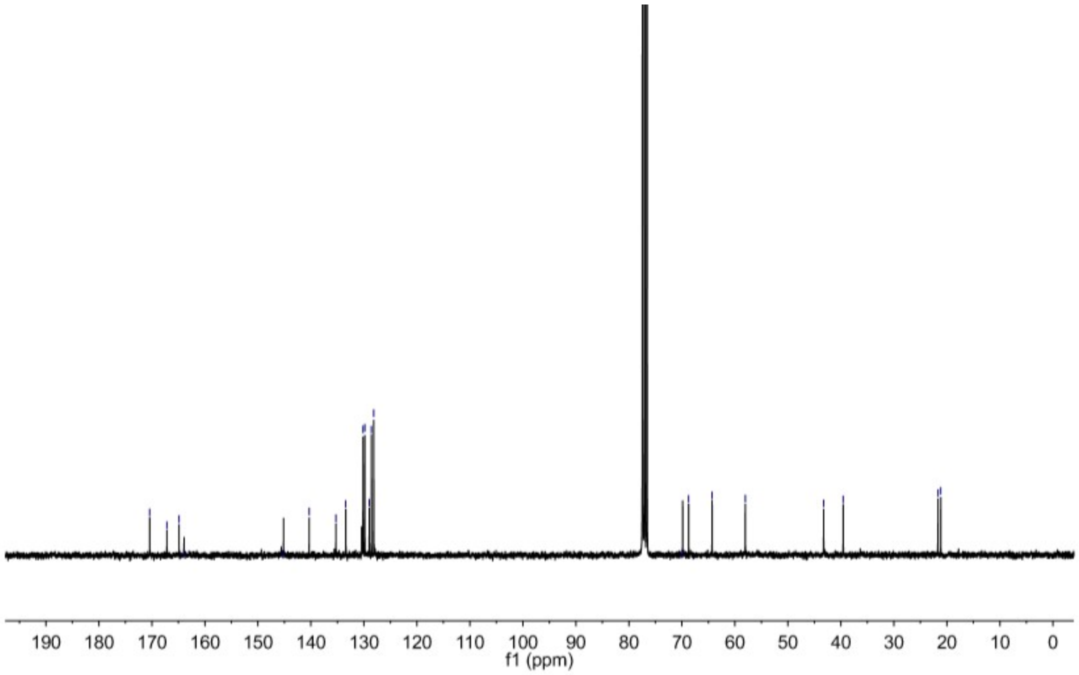

Fig. S98 ^{13}C NMR of compound **IC-Tz** ($CDCl_3$, 298K, 75 MHz)

References

1. Zegota, M., Wang, T., Seidler, C., Ng, D. Y. W., Kuan, S. L. and Weil, T., *Bioconjugate Chem.*, 2018, **29***,* 2665-2670.

2. Wang, T., Wu, Y., Kuan, S. L., Dumele, O., Lamla, M., Ng, D. Y., Arzt, M., Thomas, J., Mueller, J. O., Barner-Kowollik, C. and Weil, T., *Chem. Eur. J.*, 2015, **21***,* 228-238.

3. Ng, D. Y., Arzt, M., Wu, Y., Kuan, S. L., Lamla, M. and Weil, T., *Angew. Chem. Int. Ed.*, 2014, **53***,* 324-328.

4. Bernardim, B., Cal, P. M., Matos, M. J., Oliveira, B. L., Martinez-Saez, N., Albuquerque, I. S., Perkins, E., Corzana, F., Burtoloso, A. C., Jimenez-Oses, G. and Bernardes, G. J., *Nat. Commun.*, 2016, **7***,* 13128.

5. Wang, T., Riegger, A., Lamla, M., Wiese, S., Oeckl, P., Otto, M., Wu, Y., Fischer, S., Barth, H., Kuan, S. L. and Weil, T., *Chem. Sci.*, 2016, **7***,* 3234-3239.

6. Ravasco, J., Faustino, H., Trindade, A. and Gois, P. M. P., *Chem. Eur. J.*, 2019, **25***,* 43-59.

7. Hansen, R. E., Østergaard, H., Nørgaard, P. and Winther, J. R., *Anal. Biochem.*, 2007, **363***,* 77-82.

8. Fontaine, S. D., Reid, R., Robinson, L., Ashley, G. W. and Santi, D. V., *Bioconjugate Chem.*, 2015, **26***,* 145-152.

9. Kirchhofer, A., Helma, J., Schmidthals, K., Frauer, C., Cui, S., Karcher, A., Pellis, M., Muyldermans, S., Casas-Delucchi, C. S., Cardoso, M. C., Leonhardt, H., Hopfner, K. P. and Rothbauer, U., *Nat. Struct. Mol. Biol.*, 2010, **17***,* 133-139.

10. C. Hamers.Casterman, T. A., S. Muyldermans, G. Robinson, C. Hamers, E. Bajyana Songa, N. Bendahman and R. Hamerst, *Nature*. 1993, **363***,* 446-448.

11. Xu, L., Raabe, M., Zegota, M. M., Nogueira, J. C. F., Chudasama, V., Kuan, S. L. and Weil, T., *Org. Biomol. Chem.*, 2020, **18***,* 1140-1147.

12. MacLean, B., Tomazela, D. M., Shulman, N., Chambers, M., Finney, G. L., Frewen, B., Kern, R., Tabb, D. L., Liebler, D. C. and MacCoss, M. J., *Bioinformatics*. 2010, **26***,* 966-968.
